# Current Understanding of Probiotic Strains and Immune Function: From Gut Microbiota to Systemic Immunity

**DOI:** 10.3390/ijms27104527

**Published:** 2026-05-18

**Authors:** Maciej Piotr Szota, Katarzyna Napiórkowska-Baran, Aleksandra Wojtkiewicz, Lidia Wydeheft, Adam Wawrzeńczyk, Józef Sławatycki, Paweł Treichel, Ewa Alska, Barbara Zyśk, Krzysztof Pałgan

**Affiliations:** 1 Department of Allergology, Clinical Immunology and Internal Diseases, Collegium Medicum in Bydgoszcz, Nicolaus Copernicus University in Toruń, 85-067 Bydgoszcz, Poland; 503680@doktorant.umk.pl (M.P.S.); lidia.wydeheft@biziel.pl (L.W.); adam.wawrzenczyk@cm.umk.pl (A.W.); jozef.slawatycki@cm.umk.pl (J.S.); ewa.alska@cm.umk.pl (E.A.); palgank@cm.umk.pl (K.P.); 2 Department of Psychology, Kazimierz Wielki University, 85-867 Bydgoszcz, Poland; 3 Student Research Club of Clinical Immunology, Department of Allergology, Clinical Immunology and Internal Diseases, Collegium Medicum in Bydgoszcz, Nicolaus Copernicus University in Toruń, 85-067 Bydgoszcz, Poland; wojtkiewiczol@gmail.com (A.W.); basiaxzysk@gmail.com (B.Z.); 4 Katedra Pediatrii, Hematologii, Onkologii, Immunologii I Transplantologii Collegium Medicum in Bydgoszcz, Nicolaus Copernicus University in Toruń, 85-067 Bydgoszcz, Poland; pawel.treichel@doktorant.umk.pl

**Keywords:** probiotics, gut microbiota, immune modulation, strain specificity, systemic immunity, intestinal barrier, microbial metabolites

## Abstract

Growing evidence indicates that the gut microbiota is a central regulator of systemic immunity, acting through epithelial barrier integrity, microbial metabolites, and bidirectional signaling with innate and adaptive immune cells. Within this framework, probiotics have attracted substantial interest as tools for immune modulation; however, their effects are not uniform and should not be generalized across species or formulations. This review synthesizes current evidence on the gut microbiota–immune axis and examines how defined probiotic strains influence immune homeostasis, inflammation, and clinical outcomes. Particular emphasis is placed on strain-specific effects among lactic acid bacteria, bifidobacteria, yeast probiotics, and emerging nontraditional candidates, with attention to mechanisms involving cytokine signaling, regulatory T-cell induction, nuclear factor kappa B (NF-κB) modulation, toll-like receptor (TLR) pathways, short-chain fatty acids (SCFAs), tryptophan metabolites, and bile-acid-dependent signaling. The available literature indicates that the most meaningful immunological effects arise from precisely characterized strains acting in specific host contexts, whereas inconsistent trial design, small sample sizes, variable dosing, and poor strain resolution continue to limit translation. Overall, current data support a shift from generic probiotic use toward mechanism-based, strain-specific, and increasingly personalized strategies for immune modulation.

## 1. Introduction

The gut microbiota is increasingly recognized as a central regulator of immune development and homeostasis, shaping both mucosal and systemic immunity from early life onward. During critical developmental windows, colonization by commensal microorganisms—particularly bifidobacteria—contributes to immune education, supports tolerance, and influences susceptibility to later inflammatory and allergic disorders [1,2,3]. Conversely, disruption of microbial ecology has been associated with immune dysregulation across a wide range of conditions. Dysbiosis, commonly characterized by reduced microbial diversity, depletion of beneficial taxa, and expansion of pro-inflammatory organisms, has been linked to chronic inflammation and altered immune responses in infant inflammatory disorders, rheumatoid arthritis, inflammatory bowel disease, obesity, and impaired responses to cancer immunotherapy [4,5,6,7]. Collectively, these observations support the concept that the intestine is not merely a digestive organ, but a dynamic immunological interface through which microbial communities shape systemic inflammatory tone and host defense [8].

Mechanistically, the microbiota exerts its immunological effects through several interconnected pathways. Microbial metabolites such as short-chain fatty acids, indole derivatives, and tryptophan metabolites act as signaling molecules that promote regulatory T-cell expansion, restrain Th17-associated inflammation, and modulate cytokine networks [1,9,10]. In parallel, commensal microbes and probiotics interact with epithelial and immune cells via pattern-recognition receptors, including Toll-like receptors, thereby influencing NF-κB-dependent signaling, dendritic-cell maturation, macrophage activation, and downstream adaptive immune responses [9,11]. The integrity of the intestinal barrier represents another critical layer of this crosstalk, as disruption of epithelial integrity allows translocation of microbial components and contributes to systemic immune activation and low-grade inflammation [8,12]. Thus, the link between gut microbiota and systemic immunity is biologically plausible, mechanistically complex, and clinically relevant [8].

Within this framework, probiotics have emerged as a potential strategy for modulating host immunity through targeted manipulation of the microbiota–immune axis. However, current evidence strongly suggests that immunomodulatory effects are not a universal property of “probiotics” as a class, but rather depend on the specific biological activity of defined strains [3,13]. Selected strains have demonstrated clinically relevant immune effects; for example, *Bifidobacterium infantis* EVC001 has been associated with suppression of Th2/Th17 responses and induction of IFNβ, while *Bifidobacterium breve* M-16V and *Lacticaseibacillus rhamnosus* GG have shown anti-inflammatory and regulatory T-cell–mediated effects in both experimental and clinical settings [1,3,9]. At the clinical level, probiotics have also been investigated for their ability to enhance vaccine responses and support antitumor immunity, although results remain inconsistent and highly dependent on strain, dose, timing, and host characteristics [5,14].

This heterogeneity represents one of the major challenges in probiotic research. Results obtained with one strain cannot be extrapolated to others, even within the same species, and multi-strain formulations often produce effects that are not predictable from their individual components [13]. Furthermore, the evidence base remains limited by methodological variability, including small sample sizes, heterogeneous populations, inconsistent formulations and dosing regimens, and incomplete mechanistic characterization [5,9,14]. As a result, despite strong biological plausibility, translation into clinical practice remains limited. In this context, the aim of the present review is to critically examine the current understanding of probiotic strains and immune function, with particular emphasis on how gut microbiota–dependent mechanisms contribute to systemic immunity and why strain-specific evidence is essential for the rational development of future probiotic interventions [13].

## 2. The Gut Microbiota–Immune Axis

### 2.1. Microbiota Composition and Immune Homeostasis

The gut microbiota is increasingly regarded as an active regulator of systemic immunity rather than a community confined to local digestive functions [15,16]. Its immunological influence depends not only on overall microbial abundance, but also on the presence of metabolically specialized taxa capable of shaping epithelial signaling, immune-cell differentiation, and inflammatory tone [16,17]. At homeostasis, communities enriched in short-chain fatty acid (SCFA)-producing anaerobes—particularly *Faecalibacterium*, *Roseburia*, and selected *Clostridium* clusters—support an anti-inflammatory immune set point by sustaining microbial metabolite production and restraining the expansion of pro-inflammatory organisms [15,17]. Across immune-mediated disorders, a frequently reported signature of dysbiosis is the depletion of these bacteria together with reduced α-diversity and enrichment of taxa associated with inflammatory activity [18,19]. Comparable ecological shifts have been described in inflammatory bowel disease, rheumatoid arthritis, type 1 diabetes, and multiple sclerosis, supporting the concept that disturbed microbial composition is linked to systemic immune disequilibrium rather than to organ-specific pathology alone [17,19,20].

Microbial diversity appears to have functional significance in its own right [18,21]. Disease-focused and meta-analytic data suggest that lower diversity is associated with impaired immune homeostasis and greater inflammatory burden in autoimmune settings [18,19]. Experimental screening of individual gut species further showed that immunomodulatory functions are distributed across complementary organisms, suggesting that community diversity provides a form of functional redundancy that stabilizes host immune responses during dietary, inflammatory, or environmental stress [21]. Human longitudinal work strengthens this concept, as changes in gut microbiota composition have been associated with day-to-day dynamics of circulating immune-cell populations, thereby linking microbial ecology directly to systemic immune behavior [22]. In parallel, immune pathways also shape the microbiota itself, since T cell–dependent regulation of intestinal communities can protect against metabolic inflammation and obesity, highlighting the bidirectional nature of microbiota–immune homeostasis [23].

### 2.2. Intestinal Barrier as an Immune Interface

The intestinal epithelium is a key anatomical interface through which microbial signals may be translated into systemic immune consequences [24,25]. Barrier integrity depends on coordinated control of tight junctions, mucus production, epithelial metabolism, and antimicrobial defense, all of which are influenced by the microbiota and its metabolites [15,24,25]. Among these metabolites, butyrate is particularly important because it supports colonocyte energy metabolism, enhances mucus production, and reinforces tight-junction architecture, thereby limiting the translocation of inflammatory microbial products into the circulation [15,24,25]. When this barrier fails, dysbiosis can contribute to systemic immune activation through leakage of lipopolysaccharide and other microbial molecules that engage TLR- and NF-κB-dependent inflammatory pathways [17,25].

Evidence from Behçet’s disease provides experimental support for this mechanism [26]. Transfer of fecal microbiota from affected patients to mice lowered butyrate, propionate, and valerate levels, increased intestinal permeability, promoted circulating LPS release, and drove splenic and mesenteric Th1/Th17-skewed responses that worsened extraintestinal inflammation [26]. A complementary model in immunodeficient hosts showed that loss of B-cell- and IgA-dependent control over the gut ecosystem shifted epithelial programs away from metabolic homeostasis and toward interferon-inducible inflammatory responses, further linking barrier dysfunction to systemic immune disequilibrium [27]. These findings indicate that the epithelial barrier is not simply a physical separator, but a mechanistic checkpoint that determines whether luminal microbial signals remain compartmentalized or spill over into systemic inflammation [25,26,27].

Intestinal epithelial cells also function as immunological instructors rather than passive structural elements [28]. They deliver polarized cytokine signals to lamina propria immune cells and respond to microbial metabolites in ways that limit excessive inflammation [28]. Acetate, for example, suppresses TLR5-induced epithelial cytokine production by altering tubulin-α acetylation and downstream kinase signaling, providing a defined experimental example of how commensal-derived metabolites may prevent exaggerated responses to bacterial stimulation [28]. In parallel, microbiota-dependent crosstalk between macrophages and ILC3s promotes GM-CSF, retinoic acid, and IL-10 production, which sustains Treg homeostasis in the colon and mesenteric lymph nodes [29]. Together, these data position the intestinal barrier as a dynamic immune interface that integrates microbial composition, metabolite signaling, and host immune programming [24,28,29].

### 2.3. Microbial Metabolites in Immune Regulation

Microbial metabolites provide one of the best-characterized mechanistic links between gut microbes and systemic immunity, although the strength of evidence differs substantially between experimental models and human studies [15,16]. Among them, SCFAs are the best characterized because they reach substantial concentrations in portal venous blood—approximately 3.8 mM for acetate, 1.1 mM for propionate, and 0.29 mM for butyrate in vessels draining the colon—making direct effects on circulating immune cells biologically plausible [30]. SCFAs signal mainly through GPR41, GPR43, and GPR109A and also inhibit histone deacetylases, thereby coupling microbial fermentation to both receptor-mediated and epigenetic immune regulation [15,24,25]. At the T-cell level, propionate and butyrate promote Treg differentiation, stabilize *Foxp3* expression, and suppress pathogenic Th17 polarization through effects on cAMP/PKA/CREB, IL-6/STAT3, and RORγt-associated pathways [24,31,32]. In rheumatoid arthritis models and translational analyses, this metabolic rewiring has been associated with lower IL-1β, higher IL-10, and restoration of Th17/Treg balance; however, attribution of clinical benefit specifically to butyrate-dependent mechanisms remains partly inferential [31,32].

In addition to acetate, propionate, and butyrate, emerging evidence has drawn attention to less abundant SCFAs such as valeric acid. Valeric acid has been proposed as a gut-derived epigenetic modulator with potential relevance for inflammatory signaling and low-grade chronic inflammation, although its translational evidence base remains less developed than that of the canonical SCFAs [33].

SCFAs also regulate humoral and myeloid immunity [24,34]. In B cells, they increase acetyl-CoA availability, enhance oxidative phosphorylation and lipid metabolism, and support plasma-cell differentiation together with IgA and IgG production [34]. Accordingly, low-fiber intake or antibiotic-induced depletion of SCFA production impairs both homeostatic and pathogen-specific antibody responses, whereas restoration of microbial fermentation rescues this defect [34]. In dendritic cells and macrophages, SCFAs favor tolerogenic programs, including IL-10 production, and help dampen inflammatory signaling, thereby extending the influence of microbial metabolism well beyond the intestinal lumen [24,25].

Bile acids constitute a second major metabolic layer of immune control [16,35]. After microbial deconjugation and 7α-dehydroxylation, primary bile acids are converted into secondary metabolites with distinct immunological activities, and bile-acid signaling through receptors such as FXR and TGR5 contributes to the regulation of T-cell and myeloid-cell programs [16,35,36]. Particularly important are 3-oxoLCA and isoLCA, which can directly bind RORγt and suppress Th17 differentiation, linking microbial bile-acid metabolism to adaptive immune polarization [35]. Beyond the intestine, gut microbiome–dependent bile-acid remodeling influences CXCL16 expression on liver sinusoidal endothelial cells and controls CXCR6+ NKT-cell accumulation, with direct consequences for hepatic tumor surveillance [37]. These observations show that bile acids function not only as metabolic molecules, but also as structurally diverse immune messengers [35,37].

Tryptophan-derived metabolites add further complexity to this network [1,16]. Indole derivatives, especially indole-3-lactic acid, activate aryl hydrocarbon receptor pathways and are generally associated with reduced inflammation, restraint of inflammatory monocyte and macrophage programs, and support of mucosal tolerance [1,16]. In breastfed infants, *Bifidobacterium infantis* EVC001 was associated with indole-3-lactic acid production, suppression of Th2/Th17-skewed responses, galectin-1 induction, and upregulation of IFNβ, illustrating how a defined microbial metabolite can influence systemic immune imprinting during a critical developmental window [1]. Additional metabolites, including inosine, urolithin A, and trimethylamine N-oxide, have also been implicated in the modulation of CD8+ T-cell fitness, interferon signaling, macrophage polarization, and responsiveness to cancer immunotherapy, although these pathways remain more context-dependent and less mature translationally than SCFA signaling [36,38,39].

Metabolite signaling does not replace classical innate immune sensing; rather, both systems converge to determine whether host–microbial crosstalk remains homeostatic or becomes inflammatory [17,25]. Dysbiosis-related translocation of microbial products amplifies TLR- and NF-κB-dependent inflammatory circuits, whereas selected commensal ecosystems and microbial metabolites can restrain these same pathways and preserve tolerance [17,24,25]. Gut symbionts can also prime protective systemic immunity, as microbiota-induced IgG responses against conserved bacterial antigens enhance resistance to systemic infection [40]. Taken together, the gut microbiota–immune axis should be viewed as a multilayered network in which community composition, epithelial integrity, and metabolite signaling cooperate to regulate T cells, B cells, myeloid cells, and innate lymphoid populations, ultimately shaping the inflammatory tone of the entire organism [15,16,17,29].

**Figure 1:**
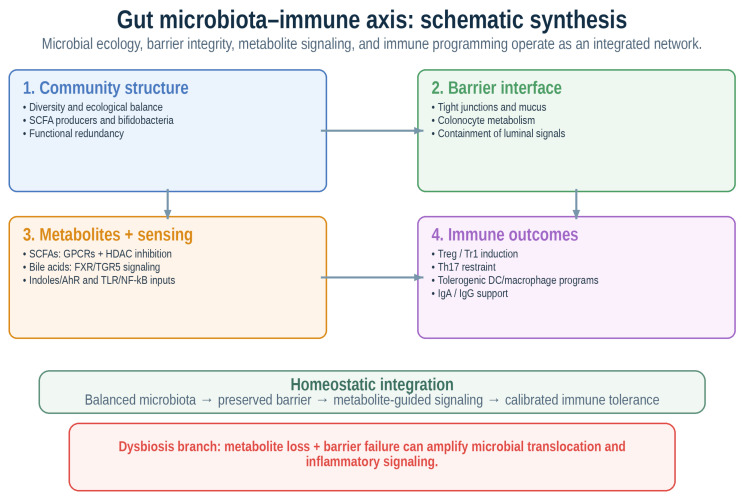
Schematic overview of the gut microbiota–immune axis discussed in this review. Homeostatic communities support barrier integrity and metabolite signaling, whereas dysbiosis can favor translocation-driven inflammation.

Overall, metabolite-centered mechanisms provide strong biological plausibility for microbiota-mediated immune regulation, but their clinical relevance should be interpreted according to the evidence type. Direct causal support is strongest in controlled experimental systems, whereas human data more often demonstrate associations between metabolite profiles, immune markers, and clinical phenotypes rather than definitive causality [15,16,17,24,25,29] (Figure 1).

## 3. Mechanisms of Immune Modulation by Probiotics

Across experimental systems, probiotic immunomodulation appears to be fundamentally strain-specific rather than species-defined. Even within a single species such as *Lactiplantibacillus plantarum*, strains can differ by 14-fold in IL-10 induction and 16-fold in IL-12 induction, while strains incorporated into the same formulation can drive distinct dendritic-cell maturation states and cytokine programs [41,42,43]. These differences arise from variation in bacterial surface structures, including lipoteichoic acids, peptidoglycan-derived muropeptides, and exopolysaccharides, as well as from secreted metabolites and epithelial intermediaries that shape innate sensing and downstream adaptive immunity [44,45,46,47,48]. Consequently, the immunological effects of probiotics should not be treated as class effects, but rather as mechanism-specific properties of defined strains.

The evidence summarized in this section derives from heterogeneous systems, including in vitro immune-cell assays, epithelial co-cultures, animal models, ex vivo human tissues, and a smaller number of clinical studies. Therefore, changes in cytokine secretion, immune-cell phenotype, or signaling-pathway activation should not automatically be interpreted as clinically validated immune modulation. Where appropriate, mechanistic observations are interpreted as preclinical or associative unless direct causal or clinical evidence is available.

### 3.1. Innate Immunity

At the level of innate immunity, several strains have been reported to alter macrophage and antigen-presenting-cell function. *Clostridium butyricum* MIYAIRI 588 selectively induced IL-10-producing intestinal macrophages through TLR2/MyD88 signaling, and this macrophage-derived IL-10 was required for protection in an experimental colitis model [49]. *L. paracasei* suppressed LPS-induced TNF-α and IL-1β in monocyte-macrophages by inducing A20, SOCS1, SOCS3, and IRAK3, suggesting a pathway by which innate recognition may contribute to inflammatory resolution [50]. A distinct macrophage-directed mechanism was reported for *L. reuteri* ATCC PTA 6475, which suppressed TNF-α production through inhibition of c-Jun/AP-1 signaling, including in macrophages from children with Crohn’s disease in remission [51]. At the level of dendritic cells, these innate effects are equally heterogeneous: bifidobacterial strains within VSL#3 promoted high IL-10/low IL-12 tolerogenic phenotypes, whereas several lactobacilli generated more stimulatory or semi-mature antigen-presenting profiles [42].

NK-cell responses appear more complex. In a comparative in vitro study, six strains—*L. casei* Shirota, *L. rhamnosus* GG, *L. plantarum* NCIMB 8826, *L. reuteri* NCIMB 11951, *B. longum* SP 07/3, and *B. bifidum* MF 20/5—all increased NK-cell activity and upregulated CD69/CD25, suggesting that some NK-activating effects may be shared despite strong heterogeneity at the cytokine level [43]. *L. casei* Shirota further preferentially activated cytotoxic CD8+ and CD56+ subsets [52]. In contrast, *L. rhamnosus* GG and *L. reuteri* DSM 17938 dampened *Staphylococcus aureus*-induced NK-cell activation, reducing IFN-γ expression and cytotoxic potential under inflammatory conditions [53]. This context dependence is mirrored in cytokine signatures. *L. plantarum* NCIMB 8826 emerged as a strong inducer of IL-12 and TNF-α, whereas *L. reuteri* DSM12246 suppressed IL-12, IL-6, and TNF-α induced by a highly stimulatory *L. casei* strain while preserving IL-10 [44,54]. Moreover, epithelial co-culture suppressed dendritic-cell activation otherwise seen with *Bifidobacterium bifidum* BGN4 and *L. casei* IBS041, emphasizing that innate immune readouts depend strongly on tissue context [55].

### 3.2. Adaptive Immunity

Adaptive immune effects are similarly strain-dependent, particularly in the induction of regulatory T-cell pathways. *B. infantis* 35624 is among the best-characterized strains in this regard, increasing CD4+CD25+Foxp3+ Tregs in mucosal and systemic compartments and transferring suppressive activity capable of inhibiting NF-κB activation and TNF-α secretion in experimental systems; in IBS, supplementation was associated with normalization of the abnormal IL-10/IL-12 ratio and clinical improvement, but these clinical changes cannot be attributed solely to the same cellular mechanism [56,57]. *L. plantarum* WCFS1 also expanded CD103+ dendritic cells and Foxp3+ Tregs, whereas *L. salivarius* UCC118 and *L. lactis* MG1363 attenuated Th2 frequencies without inducing the same Treg response, underscoring that superficially similar phenotypes may arise through different mechanisms [58]. A further example is *L. casei* Lbs2, which induced Foxp3+ Tregs through TLR2-dependent dendritic-cell signaling and ameliorated experimental colitis after adoptive transfer, with both live and heat-killed preparations remaining active [59]. By contrast, *B. breve* promoted IL-10-producing Tr1 cells via CD103+ dendritic cells and a TLR2/MyD88/IL-27 axis, demonstrating that different strains can converge on tolerance through distinct regulatory T-cell subsets [60].

Probiotic effects on Th1/Th2 balance are equally heterogeneous. Broadly, lactobacilli tend to favor more cellular, Th1-associated profiles, whereas bifidobacteria more often induce higher IL-10/IL-12 ratios and a regulatory bias, but this pattern breaks down rapidly at the strain level [43]. *L. casei* Shirota and *L. plantarum* NCIMB 8826 promoted IL-12-driven Th1 skewing, whereas *L. rhamnosus* GG did not stimulate IFN-γ to the same extent [43,52]. In allergy models, *L. paracasei* KW3110 shifted antigen-presenting cells toward a Th1-promoting phenotype with increased IL-12, reduced IL-4, and inhibition of serum IgE elevation [61]. However, not all beneficial strains act through Th1 deviation. *L. acidophilus* L-92 simultaneously suppressed IFN-γ, IL-4, and IL-10, increased TGF-β and Peyer’s patch IgA, and reduced ovalbumen-specific IgE, suggesting a broader regulatory mechanism rather than simple Th1/Th2 rebalancing [62]. Likewise, *L. salivarius* HMI001 attenuated peanut-driven Th2 responses through a high IL-10/IL-12 ratio, whereas *L. casei* Shirota achieved a similar anti-allergic effect through stronger IFN-γ/IL-12 signaling; in contrast, *L. plantarum* WCFS1 augmented IL-4 production and worsened allergic responses [63]. These ex vivo observations support biological relevance in human inflammatory tissue, but they do not by themselves establish clinical efficacy: *L. kefiri* CIDCA 8348 reduced TNF-α, IL-6, IFN-γ, and IL-13 in lamina propria T cells from patients with active IBD while increasing FOXP3+ Tregs and IL-10 [64].

### 3.3. Anti-Inflammatory Pathways

Among anti-inflammatory pathways, IL-10 occupies a central position, but its induction varies markedly across strains. Even among *L. plantarum* strains, IL-10 induction can vary over an order of magnitude [41], and bifidobacteria generally induce higher IL-10/IL-12 ratios than many lactobacilli [42,43]. Mechanistically, teichoic-acid-dependent TLR2-ERK signaling can shift macrophage responses from predominant IL-12 toward predominant IL-10, supporting the ERK/p38 balance as a context-dependent determinant of tolerogenic versus inflammatory output [65]. Exopolysaccharides from *B. adolescentis* IF1-03 activated TLR2-ERK/p38 signaling and promoted IL-10 production together with Treg differentiation, whereas the structurally distinct IF1-11 strain favored IL-6 production, Th17 polarization, and more severe inflammation [66]. Complementary mechanisms were described for *B. longum* 1714, whose exopolysaccharide selectively induced IL-10, while strain-derived indole lactic acid suppressed TLR-driven cytokine release and NF-κB activation [47].

NF-κB signaling represents a second major axis of divergence. *L. paracasei* attenuated LPS-induced inflammation by upregulating A20, SOCS1, SOCS3, and IRAK3, thereby reducing IκB phosphorylation and NF-κB nuclear translocation [50]. *L. rhamnosus* GG inhibited IκBα degradation and downregulated TLR4 expression in intestinal epithelial cells [67], whereas *L. acidophilus* inhibited TNF-α-induced tight-junction permeability through a TLR2- and PI3K-dependent inhibition of NF-κB activation [68]. Epigenetic control further refines this pathway, as *B. breve* and *L. rhamnosus* GG reduced IL-17, IL-23, and CD40 expression through decreased histone acetylation and altered accessibility of inflammatory gene promoters [48]. *B. infantis* 35624 likewise suppressed infection- and LPS-induced NF-κB activation in vivo, preceding reductions in cytokine secretion and sickness behavior, which is consistent with the hypothesis that some probiotic strains may act primarily as immune recalibrators rather than direct antimicrobials in specific contexts [56].

TLR signaling is not uniformly inhibitory, however, and its consequences depend on the molecular architecture of each strain. The lipoteichoic-acid-deficient mutant *L. acidophilus* NCK2025 lost conventional TLR2 activation yet became more anti-inflammatory, with enhanced IL-10, reduced IL-12/TNF-α, impaired dendritic-cell costimulation, and improved protection in DSS and T-cell transfer colitis [45]. By contrast, wild-type *L. plantarum* CRL1506 required lipoteichoic acid to restrain TLR3-driven intestinal inflammation and support antiviral protection [69]. Other strains amplify innate sensing in a protective manner: *L. acidophilus* NCFM induced IFN-β and antiviral defense genes in dendritic cells through TLR2-dependent endocytosis, whereas *L. rhamnosus* GG and *L. plantarum* BFE 1685 upregulated epithelial TLR2 and TLR9 expression [70,71]. Conversely, *B. longum* BB536, *B. breve* M-16V, and *L. mucosae* CRL2069 induced negative regulators such as A20, ABIN-3, IRAK-M, and MKP-1, thereby damping downstream TLR signaling [72,73]. *E. coli* Nissle 1917 provides a well-studied example of controlled pro-resolving activation rather than simple suppression: its anti-colitic effects required both TLR2 and TLR4, and its lysate induced more IL-10 and stronger downregulation of CCL24 than purified LPS alone [74,75]. Importantly, these pathways are disease-specific. In ulcerative colitis-derived immune cells, selected lactobacilli and bifidobacteria reduced IL-23/Th17-associated inflammation and AIEC persistence, whereas similar effects were largely absent in Crohn’s disease-derived cells [76]. Taken together, these data suggest that probiotic-mediated immune modulation is not a uniform anti-inflammatory effect, but a strain-defined alteration of innate sensing, cytokine balance, and T-cell differentiation, in which the same pathway can yield protective or detrimental consequences depending on host and disease context [44,45,76] (Table 1).

Taken together, the evidence for probiotic anti-inflammatory pathways is strongest when supported by direct pathway perturbation, such as receptor dependence, mutant-strain comparisons, or loss-of-function experiments in animal models. Evidence is intermediate when consistent cytokine or transcriptional changes are reproduced across cell systems or ex vivo tissues. It is weakest when pathway involvement is inferred only from changes in immune markers without causal validation. Thus, IL-10 induction, NF-κB attenuation, and TLR-dependent signaling should be interpreted as strain- and context-dependent mechanisms rather than universal anti-inflammatory properties of probiotics.

## 4. Strain-Specific Effects on Immune Function

### 4.1. Lactic Acid Bacteria

Lactic acid bacteria constitute the most densely represented probiotic group in the current evidence base, but the material summarized below shows that their biological activity is distributed across many distinct strains rather than across genera as a whole [77,78,79,80]. Reported effects span gastrointestinal and respiratory support, metabolic regulation, barrier protection, oral and vaginal health, and gut–brain signaling, with substantial variation in magnitude and consistency across indications [77,79,81,82,83,84,85,86]. Accordingly, the translational value of this group lies not in broad taxonomic labels, but in careful matching of defined strains to defined clinical contexts [77,78,79,80,87].

#### 4.1.1. *Lacticaseibacillus rhamnosus* GG (ATCC 53103; LGG), LR04, LR05, HN001, Pen, E/N, R0011, R0052; LGG Variants (GG-HF, GG-BB); IMC 501, IMC 502; MP108

Across diverse clinical settings, *Lacticaseibacillus rhamnosus* has emerged as one of the most extensively studied probiotic species, with strain-specific benefits spanning dermatologic, gastrointestinal and metabolic domains. Evidence shows that selected strains can reduce symptoms of atopic dermatitis in children, modulate the gut microbiota with improvement of irritable bowel syndrome, and may even lower the risk of gestational diabetes mellitus, while overall safety across randomized trials remains high, with serious adverse events reported only rarely [77]. As an adjunct to gastrointestinal pharmacotherapy, *L*. *rhamnosus* also enhances treatment outcomes: the combination product Lacidofil^®^ STRONG, containing *L. rhamnosus* R0011 and *Lactobacillus helveticus* R0052, increases Helicobacter pylori eradication rates when added to standard triple therapy and simultaneously reduces gastrointestinal side effects and improves health-related quality of life [88]. Among individual strains, *L. rhamnosus* GG (LGG) consistently lowers both the incidence and duration of diarrhea and improves composite gastrointestinal and respiratory outcomes—particularly in children—although effects on other isolated symptoms remain smaller or inconsistent, supporting its primary role in the prevention and management of pediatric diarrhea [89,90]. Emerging data also point to potential psychobiological effects: in a randomized, placebo-controlled trial, four weeks of *L. rhamnosus* HN001 supplementation produced trends toward improved happiness and lower perceived stress, with post hoc analyses suggesting possible sex-specific differences that require confirmation in larger, longer-term studies [91]. Together, these findings highlight the broad yet strain-dependent clinical relevance of *L. rhamnosus,* while underscoring the need for more targeted mechanistic and population-specific research.

#### 4.1.2. *Lacticaseibacillus casei* (Shirota, DN-114 001, LC1, LC03, CRL 431, Zhang, ATCC 334, ATCC 393, BL23)

Across metabolic, hepatic and neurobehavioral domains, *Lacticaseibacillus casei/paracasei* Shirota (LcS) emerges as a multifunctional probiotic capable of modulating cardiometabolic risk, protecting the gut–liver axis and influencing gut–brain pathways in both experimental and clinical settings [81,92,93,94,95,96,97,98]. In adults with elevated cardiometabolic risk, an 8-week LcS-enriched functional beverage produced modest but consistent improvements in weight, insulin sensitivity, lipid profiles and peripheral blood pressure, supporting its translational potential as a dietary adjunct for metabolic risk reduction [81]. Mechanistic studies in acute liver injury models further demonstrate that LcS attenuates hepatic and intestinal damage, restores bile acid and PPAR-related pathways, strengthens epithelial barrier integrity and reshapes the gut microbiota and metabolome, collectively indicating coordinated protection along the gut–liver axis [92,93]. Complementary evidence from alcohol-related liver injury shows improvements in dyslipidemia, normalization of iron-handling pathways and microbiota shifts toward Lactobacillus-dominant communities, linking its hepatoprotective effects to correction of dysbiosis and metabolic disturbances [94,95]. Beyond hepatic and metabolic outcomes, LcS also exerts clinically relevant effects on gastrointestinal and neurobehavioral health, reducing constipation and respiratory infections in nutrient-deprived children [96], enhancing daytime alertness and autonomic balance in adults with sleep complaints [97], and alleviating depressive symptoms in mood-disorder patients in a microbiota-dependent manner [98]. Together, these findings position LcS as a versatile probiotic with broad translational promise across metabolic, hepatic and gut–brain axes, while underscoring the importance of strain specificity and host context in determining therapeutic efficacy.

#### 4.1.3. *Lacticaseibacillus paracasei* (Lpc-37, 8700:2, F19, ATCC 55544, K71, SD1)

Across ecological, preclinical and clinical studies, *Lacticaseibacillus paracasei* is emerging as a versatile species with relevance across gut, metabolic and neurobehavioral domains. Kefir and water-kefir grains provide rich reservoirs of strains—including extracellular-polysaccharide (EPS) producers—that enhance gastrointestinal persistence and exert direct health-promoting effects, highlighting the need for continued isolation and detailed characterization to guide their use in functional foods and probiotic formulations [82]. Preclinical inflammatory bowel disease models further show that *L. paracasei*-based probiotics can improve colon length, cytokine balance and disease activity indices, with EPS-producing strains appearing particularly promising for strategies aimed at maintaining or restoring gut health [99]. Clinical findings extend this potential to neurogastroenterological and metabolic settings: synbiotic supplementation with *L. paracasei* DG and inulin improves constipation, non-motor symptoms and gut microbiota composition in patients with stable Parkinson’s disease [100], while moderate consumption of sour beer fermented with *L. paracasei* Lpc-37 increases HDL-cholesterol and stabilizes gut microbiome structure relative to a control sour beer, suggesting attenuation of alcohol-related microbial disturbances with possible cardiometabolic benefit [101]. Lpc-37 has also been explored as a psychobiotic; in healthy adults exposed to acute experimental stress, several weeks of supplementation reduce perceived stress and show trends toward improved physiological stress markers [102], whereas during prolonged academic stress, extended intake does not significantly alter perceived stress or mood despite excellent tolerability, indicating that its psychobiological effects may depend on the type and duration of the stressor [103]. Together, these findings illustrate the broad functional reach of *L. paracasei* across gut, metabolic and stress-related pathways, while underscoring the need for strain-specific mechanistic and clinical studies to define durability and context-specific efficacy.

#### 4.1.4. *Lactobacillus gasseri* (SBT2055, OLL2809, HN001, CP2305, LG08, KS-13)

Across diverse clinical contexts, *Lacticaseibacillus gasseri* demonstrates a broad but distinctly strain-specific range of effects spanning vaginal, gastrointestinal and women’s health outcomes. Oral intake of *L. gasseri* CECT 30648 enables detectable vaginal colonization and shifts the vaginal microbiota toward a Lactobacillus-dominated profile, supporting its antimicrobial activity and its use as an oral probiotic to promote vaginal health [83]. Within the gut, *L. gasseri* 345A enhances intestinal motility, counteracts opioid-induced slowing of the bowel and reduces TRPV1-mediated pain signalling; clinical observations further show improvements in abdominal pain and bowel emptying in patients with functional constipation, highlighting its therapeutic potential in this setting [104]. In women recovering from bacterial vaginosis, oral administration of *L. gasseri* TM13 and *Lacticaseibacillus crispatus* LG55 alongside metronidazole does not increase cure rates but helps restore vaginal health through gut-mediated mechanisms rather than stable vaginal colonization, underscoring the importance of individualized probiotic strategies [105]. Additional strains extend these effects to upper gastrointestinal and menopausal health: continuous intake of *L. gasseri* OLL2716 (LG21) provides preliminary evidence of improved gastric motility and autonomic regulation in individuals with delayed gastric emptying [106], while daily supplementation with *L. gasseri* CP2305 significantly reduces mild menopausal symptoms—particularly psychological and vasomotor complaints—without altering reproductive hormone levels, supporting its role as a safe, non-hormonal option for improving quality of life in middle-aged women [107]. Together, these findings highlight the functional diversity of *L. gasseri* and the importance of strain-resolved approaches to guide targeted clinical applications.

#### 4.1.5. *Lactobacillus crispatus* (CTV-05 [Lactin-V], LbV88, LBV01, LBV02, LBV03, LBV05)

Selected *Lactobacillus crispatus* strains have shown effects across genital, gastrointestinal, and immune contexts, but the reported outcomes remain strain- and indication-specific. Oral and vaginal preparations containing *L. crispatus* improve symptoms of bacterial vaginosis and vulvovaginal candidiasis—including discharge, odour, itching and irritation—while increasing vaginal lactobacilli, supporting their use as adjuncts to restore vaginal health and reduce recurrence risk [108]. In women with high-risk human papillomavirus infection, intravaginal *L. crispatus* chen-01 lowers viral load, increases clearance rates and reduces vaginal inflammation, highlighting its role in rebuilding a protective vaginal microbiota and supporting viral clearance [109]. Similarly, vaginal administration of *L. crispatus* CTV-05 (LACTIN-V) after metronidazole enhances L. crispatus colonization and prevents the rise in activated endocervical HIV target cells observed with placebo, suggesting that optimized formulations may help reduce genital inflammation and potentially lower HIV acquisition risk [110]. Oral *L. crispatus* M247 further extends these benefits by increasing high-risk human papillomavirus clearance, improving cervical cytology and promoting a lactobacillus-dominated vaginal microbiota, supporting its potential as a probiotic strategy to enhance gynaecological health [111]. Beyond the genital tract, selected strains show activity in the stomach and immune system: *L. crispatus* FSCDJY67L3 reduces Helicobacter pylori load and improves gastrointestinal symptoms without disturbing overall gut microbiota or organ function markers [112], while heat-sterilized *L. crispatus* KT-11 increases CD3^+^ T-cell counts and reduces common-cold-like symptoms—particularly fatigue—in healthy adults, suggesting immune-modulating potential that warrants larger confirmatory trials [113]. Together, these findings support a strain-resolved interpretation of *L. crispatus* effects and highlight the need for targeted clinical applications rather than broad extrapolation.

#### 4.1.6. *Lactobacillus delbrueckii* (LB-87, Lb1466, subsp. bulgaricus [GLB44, LDS1])

Selected *Lactobacillus delbrueckii* subsp. *lactis* strains have been studied across clinical, experimental, and safety contexts, with reported relevance to women’s health, neurobiology, and the gut–brain axis. Intravaginal administration during radiotherapy helps counteract radiation-induced vaginal microbiota dysbiosis by maintaining Lactobacillus dominance and limiting the expansion of opportunistic pathogens such as *Brevundimonas*, *Streptococcus* and *Prevotella*, thereby potentially reducing radiation-associated vaginal injury and supporting microbiome stability in women with gynecologic malignancies [114]. Experimental work further shows that *L. delbrueckii* exerts neuromodulatory effects: in zebrafish it reduces anxiety-like behaviour, increases *gad* gene expression in the brain and gut, and reshapes the intestinal microbiota in ways consistent with psychobiotic activity and gut–brain axis modulation [115]. Clinical evidence extends these findings to cognitive health, with supplementation of *L. delbrueckii* subsp. *lactis* CKDB001 in older adults with mild cognitive impairment improving global cognition, memory and processing speed—effects likely mediated by favourable shifts in gut microbiota composition and enhanced production of neuroactive indole-derived metabolites [116]. At the same time, safety considerations remain essential: multiple *L. delbrueckii* strains show resistance to aminoglycosides, glycopeptides, fluoroquinolones and tetracyclines, and although these resistance genes are generally not associated with mobile genetic elements—suggesting a low risk of horizontal transfer—they underscore the importance of thorough genomic characterization when strains are intended for food or probiotic use [117]. Together, these findings support further strain-level evaluation of *L. delbrueckii* rather than broad species-level extrapolation.

#### 4.1.7. *Lactiplantibacillus plantarum* (299v, Lp01, Lp02, Lp03, HEAL9, CECT 7527, CECT 7528, CECT 7529, NCIMB 8826, WCFS1, P8)

Across a broad spectrum of clinical, neurological, microbiological, and agricultural contexts, *Lactobacillus plantarum* emerges as a remarkably versatile species whose functional effects are both strain-specific and mechanistically diverse. Evidence from a systematic review and meta-analysis demonstrates that supplementation with *L. plantarum* significantly lowers total cholesterol, triglycerides, and LDL cholesterol—particularly when administered as a single-strain intervention over periods exceeding eight weeks—highlighting its potential as an adjunctive strategy for managing hyperlipidemia [84]. Beyond metabolic regulation, *L. plantarum* exhibits potent antagonistic activity against *Staphylococcus aureus* through bacteriocin-mediated membrane disruption, interference with quorum sensing, degradation of biofilms, and modulation of host immune responses, positioning it as a promising candidate for preventing device-associated infections [118]. Its influence extends to neurogastrointestinal disorders: in Parkinson’s disease, *L. plantarum* demonstrates psychobiotic properties by reshaping gut microbiota composition, strengthening epithelial barrier integrity, attenuating neuroinflammation, and modulating bile acid homeostasis, suggesting therapeutic potential that warrants further clinical validation [119]. Selected strains also show promise in autism spectrum disorder, where modulation of the gut–brain axis has been associated with improvements in gastrointestinal function and behavioral symptoms, though larger, rigorously designed trials remain essential to substantiate these early findings [120]. In agricultural systems, *L. plantarum* contributes to improved broiler performance by enhancing gut microbial balance, suppressing pathogenic species, and elevating meat quality, underscoring its value as a sustainable alternative to antibiotic growth promoters, even as optimization of dosing and strain selection continues to be needed [121]. Collectively, these studies illustrate the expansive functional repertoire of *L. plantarum*, while emphasizing that its translational potential depends critically on strain identity, host context, and mechanistic precision.

#### 4.1.8. *Limosilactobacillus reuteri* (DSM 17938, ATCC 55730, RC-14, ATCC PTA 5289, Protectis Variants [MM2-3], JCM 1112, Fn041)

Across diverse clinical and mechanistic investigations, *Limosilactobacillus reuteri* emerges as a taxon whose effects are strikingly context-, strain-, and host-dependent. In neonates exposed to antibiotics, supplementation with DSM 17938 produced only modest clinical benefits, yielding slight reductions in functional gastrointestinal disturbances without altering crying duration, sleep patterns, or global microbiota structure, underscoring the limited and highly specific nature of its early-life activity [122]. Broader conceptual work further positions *L. reuteri* as an adaptable gut commensal capable of shaping mucosal and systemic immunity through metabolically diverse and host-interactive pathways, while also highlighting that its actions may range from protective to potentially pathogenic depending on host genetics, diet, and inflammatory state [79]. Yet, in certain clinical settings, the organism demonstrates robust therapeutic potential: in a large multicenter trial, DSM 17938 consistently reduced antibiotic-associated diarrhea in children treated with amoxicillin–clavulanate, with the strongest effects observed in younger patients and those with acute otitis media, suggesting a clinically meaningful adjunctive role during antibiotic exposure [123]. Mechanistic work with strain CCFM1388 extends this functional spectrum beyond classical gastrointestinal outcomes, revealing a microbiota-dependent metabolic axis in which modulation of bile acid profiles and intestinal cholesterol absorption enhances exercise endurance in mice, with parallel human data indicating improvements in muscle strength despite stable testosterone levels [124]. Complementing these findings, DSM 17648—administered as a postbiotic—significantly improved *Helicobacter pylori* eradication rates and reduced treatment-related digestive symptoms when added to standard triple therapy, supporting its promise as a safe and targeted adjunct in infection management [125]. Together, these studies illustrate the remarkable functional breadth of *L. reuteri*, while simultaneously emphasizing that its clinical utility is inseparable from strain identity, host context, and mechanistic specificity.

#### 4.1.9. *Limosilactobacillus fermentum* (PCC, CECT 5716, ME-3, Lf2, RC-14 [Older Classification])

Across clinical trials and mechanistic reviews, *Limosilactobacillus fermentum* emerges as a species with wide-ranging but strongly strain-specific probiotic and postbiotic potential. In a randomized, double-blind, placebo-controlled crossover trial, the heat-killed paraprobiotic HDB1098 significantly lowered serum alcohol and acetaldehyde levels and alleviated multiple hangover symptoms, demonstrating measurable physiological and subjective benefits and positioning it as a promising candidate for hangover mitigation pending larger confirmatory studies [126]. In gastrointestinal settings, supplementation with *L. fermentum* MN LF23 did not enhance *Helicobacter pylori* eradication when added to standard quadruple therapy, yet it meaningfully reduced gastrointestinal symptoms and supported the recovery of beneficial gut bacteria, indicating symptomatic and microbiota-supportive value despite unproven effects on eradication outcomes [127]. Beyond the gut, a topical formulation containing heat-treated *L. fermentum* LM1020 improved hair density and thickness and restored scalp microbiome balance in individuals with androgenetic alopecia, highlighting its promise as a postbiotic dermatologic intervention requiring further mechanistic and long-term evaluation [128]. Broader reviews reinforce the species’ mechanistic versatility: *L. fermentum* CECT5716, a human-milk–derived strain, exhibits antimicrobial activity, strengthens epithelial barrier integrity, modulates immune responses and promotes bifidogenic effects, with accumulating evidence supporting its safety and potential to reduce gastrointestinal and respiratory infections in infants, though larger, rigorously controlled studies remain essential [87]. Additional syntheses indicate that *L. fermentum* strains can modulate oxidative stress, inflammation and gut-derived metabolic pathways to improve glucose homeostasis and attenuate diabetes-related complications, while also possessing diverse antioxidant mechanisms capable of counteracting redox imbalance across metabolic, inflammatory, neurodegenerative and gastrointestinal disorders [129,130]. Together, these findings underscore the broad therapeutic promise of *L. fermentum* while emphasizing the need for strain-resolved, rigorously controlled clinical studies to define its true translational potential across metabolic, gastrointestinal, dermatologic and systemic applications.

#### 4.1.10. *Levilactobacillus brevis* (KB290, CD2, IOEB 9809, ATCC 14869)

Across diverse clinical and preclinical contexts, strains and derivatives of *Levilactobacillus brevis* demonstrate a spectrum of targeted, system-specific benefits that highlight their emerging therapeutic versatility. Daily intake of heat-killed *L. brevis* KB290 combined with β-carotene did not reduce overall influenza incidence in a large randomized, double-blind, placebo-controlled trial, yet markedly lowered infection rates among adults under 40, revealing an age-dependent protective effect and suggesting that immunomodulatory benefits may be concentrated in younger hosts [131]. Complementing these population-specific antiviral effects, high-dose EPS1 derived from *L. brevis* M-10 robustly restored physiological, biochemical and histological parameters in a DSS-induced colitis model, attenuating inflammation, repairing epithelial barrier integrity and rebalancing gut microbiota composition, thereby positioning this exopolysaccharide as a promising microbiota-derived candidate for ulcerative colitis intervention [132]. Extending the organism’s functional breadth to the oral cavity, four-week supplementation with *L. brevis* CD2 in a randomized, double-blind, placebo-controlled trial significantly improved gingival bleeding, plaque accumulation, salivary flow, buffering capacity and multiple metabolic biomarkers, with several benefits persisting beyond washout, underscoring its potential as a multifunctional probiotic for maintaining oral health [133]. Together, these findings illustrate the capacity of *L. brevis* strains and their bioactive components to modulate host physiology across respiratory, intestinal and oral ecosystems, while emphasizing the need for mechanistic and translational studies to define their context-specific modes of action and clinical relevance across diverse populations.

#### 4.1.11. *Ligilactobacillus salivarius* (UCC118, Ls-33, EF204, CICC 23174, CETC 5434, PS7)

Across mechanistic, industrial and clinical studies, *Ligilactobacillus salivarius* is emerging as a host-adapted probiotic with broad abilities, including tolerance to acid and bile, antimicrobial and antioxidant activity, and the capacity to shape microbial communities, as shown increasingly through multi-omics work that clarifies how these functions arise [85]. At the same time, many promising strains remain underused because they are sensitive to production and stabilization steps, highlighting the need for better understanding and improved preservation methods to support reliable large-scale manufacturing [134]. Clinical trials further expand its relevance: oral *L. salivarius* CECT5713 increases term pregnancy rates in couples with unexplained infertility, accompanied by coordinated immune changes in both partners [135]; *L. plantarum* CCFM1214 and *L. salivarius* CCFM1215 reduce halitosis by lowering volatile sulfur compounds, suppressing *Fusobacterium nucleatum*, and stabilizing the oral microbiota [136]; and *L. salivarius* V4II-90 enhances eradication of Group B *Streptococcus* colonisation during pregnancy, reducing the need for intrapartum antibiotics [137]. Together, these findings show that *L. salivarius* strains can influence host biology across reproductive, oral and perinatal settings, while emphasizing the need for deeper mechanistic insight and better bioprocessing strategies to fully realize their clinical and industrial potential.

#### 4.1.12. *Lactobacillus helveticus* (R0052, MTCC 5463, ATCC 15009, LAFTI L10, Rosell-52)

Across metabolic, immune, gastrointestinal and neurocognitive domains, converging evidence shows that *Lactobacillus helveticus*–*Bifidobacterium longum* interventions exert targeted yet diverse physiological effects in healthy and clinical populations. In women with PCOS, eight-week supplementation consistently improved hormonal balance, oxidative stress and low-grade inflammation despite limited changes in outward symptoms, suggesting that probiotic modulation of core biochemical pathways may precede visible clinical benefit [86]. Complementing these findings, twelve-week intake of *L. helveticus* R0052 and *B. longum* R0175 in female dancers produced clear shifts in fecal metabolic pathways—particularly those linked to polyphenol metabolism—highlighting strain-specific metabolic activity even in the absence of symptom improvement [138]. Beyond metabolic effects, heat-sterilized *L. helveticus* GCL1815 reduced common-cold symptom days and activated key dendritic-cell subsets, indicating enhanced antiviral readiness in healthy adults [139]. Additional postbiotic formulations demonstrated benefits across gut and mood outcomes: *L. helveticus* CP790-fermented milk reduced Desulfobacterota abundance, improved stool consistency and modestly enhanced emotional well-being in adults with constipation tendencies [140], while heat-killed *L. helveticus* MCC1848 selectively improved positive mood states without affecting sleep, fatigue or negative affect [141]. Extending these observations to neural function, a proof-of-concept crossover trial showed that a multi-strain probiotic subtly modulated brain activity and connectivity during acute psychosocial stress, particularly in early stress-response phases, without altering cortisol or cognitive performance [142]. Together, these studies illustrate the capacity of *L. helveticus*–*B. longum* strains and postbiotics to influence interconnected metabolic, immune, microbial and neural pathways, underscoring the need for larger mechanistic trials to define durability, dose–response relationships and population-specific responsiveness.

#### 4.1.13. *Pediococcus* (*P. acidilactici* [MA 18/5M, K15]; *P. pentosaceus* [5K, LP28, ATCC 25745])

Across reviews and clinical trials, *Pediococcus* species are emerging as flexible candidates for both food biotechnology and targeted probiotic use. Broad evaluations of *P. pentosaceus* describe antioxidant, cholesterol-lowering, antimicrobial and immune-modulating activities, together with wide applicability in fermented foods, highlighting the need for deeper genomic, functional and process-level work to support reliable, strain-specific applications [80]. A second review reinforces this view, emphasizing the species’ metabolic range and growing relevance across industrial and health-focused settings, and calling for continued mechanistic and process-optimization studies to enable scalable, high-quality use [143]. Clinical findings extend this potential to *P. acidilactici* strains: although year-long supplementation with heat-killed K15 did not reduce respiratory infections in preterm infants, exploratory signals in infants with older siblings suggest that certain subgroups may benefit, underscoring the need for larger trials [144]. In contrast, *P. acidilactici* CCFM6432 improved anhedonia and overall depressive symptoms, alongside changes in reward-anticipation brain activity, pointing to a promising pathway for mood-related interventions [145]. Finally, *P. acidilactici* GR-1 accelerated the reduction of copper and nickel levels in metal-exposed workers while improving antioxidant status, lowering inflammation and reshaping gut microbial and metabolic profiles, positioning GR-1–enriched yogurt as a practical, low-cost approach for reducing heavy-metal burden in at-risk populations [146]. Together, these findings show the broad functional reach of Pediococcus across metabolic, immune, gut and neurobehavioral systems, while emphasizing the need for larger mechanistic studies to define durability, target populations and translational potential.

### 4.2. Bifidobacterium spp.

Within the current evidence base, bifidobacteria stand out as early-life-associated commensals with particular relevance to immune education, barrier maintenance, and mucosal tolerance [147,148,149,150,151]. The strains summarized below have been linked to outcomes ranging from necrotizing enterocolitis prevention and infant colic to vaccine responsiveness, low-grade inflammation, metabolic regulation, and gut–brain signaling, but these effects vary markedly between taxa and between strains within the same species [147,148,149,150,151,152,153,154,155]. Overall, this section reinforces that bifidobacterial efficacy is biologically plausible and clinically important, yet fundamentally strain-specific and host-dependent [156,157,158].

#### 4.2.1. *Bifidobacterium animalis* subsp. *lactis* (BB-12, B420, NCC2818, B94)

Across diverse clinical contexts, *B. animalis* subsp. *lactis* has emerged as a versatile probiotic with gastrointestinal, immunological and metabolic benefits, albeit in a highly strain-specific manner. In neonatology, *B. lactis* strains significantly reduce the incidence of necrotizing enterocolitis in preterm infants (relative risk ~0.11, *p* < 0.001), with particularly strong efficacy in very low birth weight neonates [147]. In term infants, the well-studied strain BB-12 consistently alleviates functional gastrointestinal disorders: a 4-week trial in colicky exclusively breastfed infants showed that BB-12 supplementation led to ≥50% reductions in daily crying duration (vs. placebo, *p* < 0.05), alongside improved sleep and normalized bowel habits [152]. Mechanistically, these benefits were associated with increased gut Bifidobacterium abundance, higher butyrate production, and suppressed expansion of proteobacterial taxa [159]. By contrast, another *B. lactis* strain (NCC2818) showed no efficacy in adults with constipation, underscoring that not all strains of this species confer the same benefits [160]. Certain *B. lactis* also exhibit metabolic activity: strain B420, for example, significantly decreased body fat mass (~4%) and waist circumference (~2.4%) in overweight adults [153]. Immunologically, *B. lactis* can enhance mucosal defenses and vaccine responsiveness: BB-12 boosted antigen-specific IgG titers (*p* < 0.001) and secretory IgA levels in infants receiving oral vaccines [148], and also upregulated innate antimicrobials in the gut (e.g., human β-defensin-2, cathelicidin LL-37) [161]. Additional strains reinforce the intestinal barrier and deter pathogens—for instance, *B. lactis* HN019 competitively excludes enteropathogenic *E. coli*, while BB-12 increases tight-junction protein expression to strengthen epithelial integrity [162]. Together, these findings highlight the broad clinical potential of *B. animalis* subsp. *lactis* across age groups and conditions, while emphasizing that efficacy is highly strain-dependent and context-specific, underscoring the need for precise, evidence-based strain selection in practice [156,157].

#### 4.2.2. *Bifidobacterium longum* (BB536, NCC3001, NCC2705)

Spanning gut, immune, and even neurobehavioral domains, *B. longum* exhibits broad probiotic potential, yet different strains within this species demonstrate distinct therapeutic profiles. The immunostimulatory strain BB536 is notable for enhancing host resistance to infections: in preschool children, daily BB536 intake over 10 months reduced the duration of upper respiratory illnesses by ~46% (with significantly shorter sore throat episodes) [154,163]. BB536 consistently augments Th1-type immunity—increasing interferon-γ production and plasmacytoid dendritic cell activation in infants and adults [164,165]—and has even been shown to maintain natural killer cell activity in frail elderly patients [165,166]. These immune effects correlate with microbiome shifts: BB536 supplementation durably increased beneficial taxa (e.g., Faecalibacterium) while suppressing potential pathogens (Clostridium, coliforms), changes that persisted for weeks after supplementation stopped [167,168]. In contrast to BB536’s immune-boosting role, other *B. longum* strains target the gut–brain axis. Notably, strain NCC3001 has demonstrated psychotropic benefits in irritable bowel syndrome: in a pilot RCT, NCC3001 significantly reduced comorbid depression scores in IBS patients (64% of the probiotic group responded vs. 32% of placebo) and modulated brain activation patterns related to mood regulation [155,169]. *B. longum* strains also exhibit ancillary benefits such as promoting skin barrier function—for example, non-replicating *B. longum* cells were shown to induce keratinocyte differentiation and β-defensin production in vitro [170,171], suggesting possible dermatologic applications. Together, these findings position B. longum as a multifaceted probiotic species with strain-dependent effects ranging from enhanced mucosal immunity to improved psychosocial well-being. At the same time, the divergent outcomes of BB536 vs. NCC3001 underscore the importance of strain specificity and host context in determining clinical efficacy [157,158].

#### 4.2.3. *Bifidobacterium longum* subsp. *infantis* (35624)

*B. longum* subsp. *infantis* 35624 (commonly termed *B. infantis* 35624) exemplifies an anti-inflammatory probiotic strain with benefits across gastrointestinal and systemic inflammatory conditions. In multiple patient populations, 35624 supplementation has consistently attenuated pathological inflammation: this strain increases the IL-10/IL-12 ratio and significantly lowers pro-inflammatory biomarkers (including plasma C-reactive protein, TNF-α, and IL-6) [149]. These immune-modulatory effects translate into clinical improvement in disorders characterized by immune dysregulation. In adults with irritable bowel syndrome, daily *B. infantis* 35624 (10^9^–10^10^ CFU) led to significant symptom reductions over 8 weeks [57], accompanied by normalization of the peripheral cytokine profile. Similarly, adjunct use of 35624 has shown benefit in ulcerative colitis and even in extra-intestinal conditions like psoriasis and chronic fatigue syndrome, where systemic inflammation is a component [172]. Notably, *B. infantis* 35624 may achieve greater efficacy when used in combination with other synergistic strains or prebiotics, as one analysis suggests it is “more effective in combination therapies than alone” in the heterogeneous IBS population [173]. Mechanistic studies indicate that *B. infantis* also supports intestinal barrier integrity (via TLR-2/p38 MAPK signaling that upregulates tight junction proteins) and helps rebalance gut microbiota composition [157,174]. There are clear population- and disease-specific nuances to its effects: for example, certain immune pathways modulated by 35624 (such as the IL-23/Th17 axis) are relevant in ulcerative colitis but not in Crohn’s disease, aligning with clinical trial failures in the latter [76]. In summary, *B. infantis* 35624 stands out as a potent immunoregulatory probiotic strain that can ameliorate a range of inflammatory conditions. At the same time, its benefits are context-dependent, reinforcing the principle that careful strain selection and matching to the target patient population are essential for translational success [157,158].

#### 4.2.4. *Bifidobacterium breve* (M-16V, B-3, BR03)

*Bifidobacterium breve* is emerging as an important probiotic species with distinct strain-specific benefits in early-life nutrition, allergy prevention, and metabolic health. In neonatal care, *B. breve* has shown promise for preventing intestinal pathology, although its efficacy hinges on infant maturity: clinical data indicate that supplementation with *B. breve* (e.g., strain M-16V) significantly lowers necrotizing enterocolitis rates in premature infants of ≥28–34 weeks gestational age (RR ~0.43, *p* = 0.019), whereas in extremely preterm infants (<28 weeks) the same intervention is not clearly effective [147,150]. Beyond the NICU, B. breve M-16V has demonstrated immunomodulatory effects conducive to allergy prevention. This strain can suppress pro-allergy cytokines (IL-4, IL-5) while inducing IL-10 and promoting regulatory T-cell development, shifting the infant immune balance toward a tolerogenic (Th1/Th2-balanced) profile [175,176]. Consistent with these mechanisms, early-life use of M-16V was associated with improvements in allergic symptoms and a healthier gut microbiota in infants predisposed to atopy [175]. Other *B. breve* strains have shown diverse benefits in older populations: for instance, strain B-3 has been investigated for metabolic and dermatologic effects, with one study reporting reductions in serum cholesterol and eczema severity in adults receiving *B. breve* B-3 or the related strain BR03 [177]. Additionally, *B. breve* influences gut ecosystem function by producing acetate and other short-chain fatty acids that support intestinal barrier integrity. One strain uniquely modulated fecal butyrate levels in a manner dependent on the host’s baseline SCFA profile [178], hinting that *B. breve*’s metabolic impacts may vary with individual microbiome context. Collectively, these findings underscore *B. breve*’s potential as a multi-domain probiotic (from infant gut health to adult metabolic and immune support) while reinforcing that its benefits are strain-specific and often contingent on host factors such as age and baseline immunity. Accordingly, precision in selecting the appropriate *B. breve* strain for a given clinical indication is essential to achieve consistent therapeutic outcomes [150,157].

#### 4.2.5. *Bifidobacterium bifidum* (R0071, BGN4, PRL2010)

A prominent early colonizer of the infant gut, *Bifidobacterium bifidum,* contributes to mucosal homeostasis and pathogen defense, but clinical benefits again depend on the specific strain and context. Certain *B. bifidum* strains excel at inhibiting pathogens and reinforcing the gut barrier. For example, strain BGN4 produces bacteriocins and effectively competitively excludes enteric pathogens, including *E. coli* and *Listeria*, thereby helping to protect the intestinal niche [151]. *B. bifidum* also supports the physical integrity of the gut lining; in vitro studies and animal models show that *B. bifidum* can upregulate tight junction proteins and mucin utilization, improving barrier function and nutrient absorption [167,179]. Some strains have demonstrated immuno-stimulatory properties: supplementation with B. bifidum in healthy adults enhanced innate immune activity, as evidenced by significantly increased phagocytosis of *E. coli*, an effect that intriguingly persisted for up to six weeks after probiotic discontinuation [180]. In infants with a familial risk of atopy, early *B. bifidum* exposure has been associated with lower eczema incidence (e.g., 18% vs. 40% in one trial of BGN4, *p* = 0.048) [181,182], potentially via modulation of immune development and gut-skin axis signals. Moreover, *B. bifidum* influences metabolic outputs of the microbiota: a four-week course of *B. bifidum* was shown to reshape the gut microbial composition (increasing health-associated families like *Ruminococcaceae* while decreasing pro-inflammatory taxa) and to modulate fecal short-chain fatty acid levels, including butyrate [178,183]. Notably, the impact of *B. bifidum* on butyrate differed depending on individuals’ baseline SCFA concentrations, highlighting an element of personalized response [178]. Overall, while *B. bifidum* is less frequently singled out in clinical trials compared to other bifidobacterial species, existing evidence suggests it plays a supportive role in maintaining gut barrier integrity and immune equilibrium. These benefits, however, are realized only by specific strains under appropriate conditions, underlining the general need for careful strain-level identification and matching to clinical targets in probiotic therapy [157,158].

### 4.3. Yeast Probiotics

Compared with bacterial probiotics, yeast-based interventions remain a narrower but mechanistically distinct part of the field [184,185,186]. The evidence summarized below shows that the strongest and most mature support centers on *Saccharomyces boulardii* CNCM I-745 in acute infectious and antibiotic-associated gastrointestinal settings, whereas *Saccharomyces cerevisiae* CNCM I-3856 appears more phenotype-specific, particularly in constipation-predominant IBS, and other *Saccharomyces* strains currently provide only isolated or preliminary signals [184,185,186,187,188,189,190,191,192,193,194,195,196,197,198,199,200,201,202,203,204,205,206,207,208,209,210,211,212]. Overall, this subsection again underscores that efficacy and safety must be interpreted at the strain and indication level rather than generalized across probiotic yeasts as a category [187,199,201,207,212].

#### 4.3.1. *Saccharomyces boulardii* CNCM I-745

Across meta-analyses, randomized trials, and mechanistic studies, *Saccharomyces boulardii* CNCM I-745 emerges as the most extensively supported probiotic yeast in the current evidence set, with its strongest human evidence centered on antibiotic-associated diarrhea, pediatric acute gastroenteritis, and *Clostridioides difficile* prevention. Meta-analyses show roughly 48–57% reductions in antibiotic-associated diarrhea and a marked reduction in *C. difficile* infection in China-specific analyses, together with about a one-day shortening of pediatric acute gastroenteritis duration [187,188,189]. Additional randomized data suggest benefit in direct comparison with *Bacillus clausii* in pediatric gastroenteritis and improved growth and feeding tolerance in formula-fed preterm infants [190,191]. Mechanistically, CNCM I-745 combines yeast-specific protease-mediated toxin neutralization with microbiota remodeling toward short-chain-fatty-acid-producing taxa, preservation of fecal secondary bile acids during antibiotic exposure, support of epithelial barrier integrity, trophic enhancement of brush-border enzyme activity, and attenuation of inflammatory signaling through NF-κB inhibition and Nrf2 activation [184,192,193,194,195,196]. These local effects also extend across broader gut–immune and gut–metabolic axes, with preclinical data suggesting reduced endotoxemia, hepatic steatosis, and low-grade inflammation [197]. Distinct from bacterial probiotics, the strain is intrinsically resistant to antibacterial drugs, can therefore be co-administered during antibiotic therapy, and shows rapid yet transient gastrointestinal passage rather than durable colonization [185,198]. Safety is generally excellent in children and adults, but rare *Saccharomyces* fungemia has been documented, mainly in critically ill, catheterized, or severely immunocompromised patients, making such populations inappropriate for routine use [199]. At the same time, its effects do not appear to extend reliably to chronic inflammatory conditions, as relapse prevention in Crohn’s disease remains negative [200]. Together, these findings position CNCM I-745 as the most clinically mature probiotic yeast for acute infectious and antibiotic-associated gastrointestinal disorders, while also underscoring that its strongest value lies in short-term interruption of diarrheal and dysbiosis-related cascades rather than in disease modification of established inflammatory bowel disease.

#### 4.3.2. *Saccharomyces cerevisiae* CNCM I-3856

Across randomized controlled trials, an individual-patient-data meta-analysis, and supporting mechanistic studies, *Saccharomyces cerevisiae* CNCM I-3856 emerges as a phenotype-specific probiotic yeast with its clearest value in irritable bowel syndrome with constipation rather than in IBS as a whole. In the largest trial, 8 weeks of supplementation significantly increased abdominal pain responder rates and improved quality of life in IBS-C [201], while the IPD meta-analysis confirmed clinically relevant reductions in abdominal pain/discomfort and bloating that exceeded the 10% threshold generally considered meaningful [202]. Additional trials support improvement in pain, bloating, and stool consistency, but also show that efficacy attenuates or disappears when unselected IBS populations are analyzed without regard to constipation phenotype [203,204]. Mechanistically, CNCM I-3856 appears to act through local PPARα-mediated visceral analgesia with a delayed onset, alongside saccharolytic activity that generates short-chain fatty acids and other metabolites with potential prokinetic effects on intestinal transit [186]. Experimental work also suggests anti-infective activity against enterotoxigenic *Escherichia coli*, and human recovery studies show that the strain survives gastrointestinal passage and can be detected in vaginal samples after oral administration [205,206]. Safety across IBS trials has been generally favorable, with mostly mild gastrointestinal adverse events and no consistent signal of serious toxicity [201,203,204]. Together, these findings support CNCM I-3856 as a strain-specific option for constipation-predominant IBS, while underscoring that its benefits should not be generalized to diarrhea-predominant or unselected IBS populations.

#### 4.3.3. Other *Saccharomyces* Strains

In the current evidence set, several additional *Saccharomyces* strains showed isolated strain-specific signals but not enough data for a synthesis comparable to CNCM I-745 or CNCM I-3856. *S. cerevisiae* var. *boulardii* CNCM I-3799 reduced recovery time in children with acute diarrhea in one multicenter randomized trial [207]. By contrast, *S. boulardii* CNCM I-1079 did not improve academic stress outcomes in healthy medical students and was associated instead with increased pulse rate and lower salivary serotonin in secondary analysis [208,209]. *S. boulardii* strain Unique 28 showed apparent benefit in a small clinical report on acute diarrhea, with supportive toxicology data in rats, but the absence of a robust controlled evidence base limits clinical interpretation [210,211]. Finally, *S. cerevisiae* SC-2201 showed anti-inflammatory and tumor-attenuating effects only in a colorectal cancer mouse model [212]. Together, these findings suggest that several non-I-745/I-3856 yeast strains may possess biologically interesting properties, but the current human evidence remains too sparse, heterogeneous, or methodologically limited to support confident clinical positioning.

#### 4.3.4. *Saccharomyces pastorianus*, *Debaryomyces hansenii*, *Kluyveromyces marxianus*, and *Yarrowia lipolytica*

At present, comparable strain-specific syntheses cannot be provided for *Saccharomyces pastorianus*, *Debaryomyces hansenii*, *Kluyveromyces marxianus*, or *Yarrowia lipolytica*, because no eligible studies meeting the report’s inclusion criteria were identified for these organisms.

### 4.4. Additional Bacterial Candidates

#### 4.4.1. *Streptococcus salivarius* K12

Across randomized trials, mechanistic studies, and safety evaluations, *Streptococcus salivarius* K12 emerges as one of the best-characterized oral probiotics, with its strongest evidence centered on recurrent pharyngotonsillitis prevention and oral mucosal protection. K12 exerts strain-specific antimicrobial activity through the megaplasmid-encoded lantibiotics salivaricin A2 and salivaricin B, and through the recently characterized antibiotic salivabactin, which broadens activity against *Streptococcus pyogenes* and other upper-airway pathogens [213,214]. These direct antagonistic effects are complemented by host-directed actions, including NF-κB inhibition, suppression of epithelial IL-8 signaling, attenuation of IL-6 and IL-8 release in pathogen-stimulated gingival fibroblasts, and induction of systemic and mucosal immune responses involving IL-10, regulatory T cells, salivary IFN-γ, and post-exercise sIgA [215,216,217,218,219]. Clinically, open-label, retrospective, and controlled pediatric studies—particularly in children with recurrent streptococcal disease—reported large reductions in pharyngotonsillitis and, in some studies, acute otitis media during continuous daily administration, often in the range of 70–90% for selected infectious endpoints, with some persistence after treatment cessation [220,221,222]. However, the largest placebo-controlled school-based trial found only a nonsignificant reduction in group A streptococcal pharyngeal carriage, and systematic-review-level appraisal suggests that much of the positive respiratory literature is limited by open-label designs, no-treatment comparators, and heterogeneous dosing schedules [223,224]. Beyond the upper respiratory tract, K12 reduced severe radiation-induced oral mucositis in patients with head and neck tumors, improved mycological cure rates as adjunctive therapy in oral candidiasis, and reduced clinical signs of denture stomatitis [225,226,227]. Colonization appears transient rather than permanent, with persistence for days to weeks and increased oral K12 abundance during supplementation without major restructuring of the overall salivary microbiome [228,229]. Safety has been consistently favorable in both human and preclinical studies, with no meaningful toxicological signal identified in the available evidence base [230,231]. Together, these findings position K12 as a clinically useful oral probiotic whose benefits seem strongest in high-risk recurrent upper-airway and oral-disease settings, while also underscoring that efficacy is highly dependent on study design, population selection, and dosing continuity.

#### 4.4.2. *Streptococcus salivarius* M18

Across mechanistic and clinical studies, *Streptococcus salivarius* M18 emerges as a more specifically anti-cariogenic and periodontal-oriented oral probiotic than K12. Its strain-specific profile is driven by a distinct megaplasmid-associated bacteriocin repertoire together with dextranase and urease activities that directly target plaque architecture and local acidification [232]. In vitro comparisons indicate that M18 is more potent than K12 against *Streptococcus mutans* and shows consistent inhibitory activity across several periodontitis-associated organisms [233,234]. Like K12, M18 can also attenuate inflammatory mediator release from pathogen-stimulated gingival fibroblasts, suggesting that its effects extend beyond direct antimicrobial competition [216]. Clinically, M18 reduced plaque scores in caries-active children over 3 months, lowered salivary *S. mutans* counts and improved buffering capacity in preschool children, and improved probing depth, bleeding on probing, and plaque index when used as an adjunct after non-surgical periodontal therapy [232,235,236]. Colonization is dose-dependent, and in some individuals M18 can transiently replace much of the indigenous *S. salivarius* population without substantially altering overall oral microbiome structure [237]. Available safety data are limited but consistently favorable, with only minor non-serious adverse events reported in human studies [232,235,236]. Together, these findings identify M18 as a targeted oral probiotic with its clearest translational value in caries-prone and periodontal populations, particularly where enzymatic plaque disruption and pH modulation can complement bacteriocin-mediated pathogen control.

These human data should be interpreted cautiously because the available studies differ in population, baseline oral health status, formulation, dose, duration, and adjunctive periodontal or dental interventions. Therefore, the apparent consistency of M18 effects should not be generalized to all *S. salivarius* strains or to broader systemic immune outcomes without larger, strain-specific trials using harmonized endpoints.

#### 4.4.3. *Streptococcus thermophilus*

Across preclinical and limited human studies, *Streptococcus thermophilus* emerges as a highly strain-dependent probiotic whose relevant effects span pathogen suppression, mucosal support, immunomodulation, and potentially tumor-related pathways. A core mechanism is lactic acid production, which not only suppresses *Clostridium difficile* growth but also reduces toxin A expression at sub-bactericidal concentrations, while selected isolates additionally produce bacteriocins such as the CHCC 3534 factor and thermopillin 110 with activity against enteric and skin-associated pathogens [238,239,240]. Other strain-specific functions include β-galactosidase-dependent suppression of colorectal tumorigenesis, lactate-driven enhancement of goblet-cell mucus programs via KLF4, and divergent immune signaling profiles ranging from monocyte TLR downregulation to macrophage activation through P38/ERK/NF-κB pathways [241,242,243,244]. Yet translation remains limited: the clearest human study showed that *S. thermophilus* BT01 survived gastrointestinal transit and reduced fecal urease activity in healthy adults, whereas most efficacy data—including protection in *C. difficile* infection and anti-tumor effects—derive from animal or in vitro systems [238,241,245]. Importantly, the colorectal cancer literature is internally inconsistent, with one study demonstrating β-galactosidase-dependent tumor suppression and another showing no protection together with immunosuppressive mucosal transcriptional changes, indicating that species-level generalization is inappropriate [241,246]. Available strain-level safety signals are reassuring, including the absence of transmissible virulence or antibiotic-resistance determinants in genomic analysis of MCC0200, although formal toxicological datasets for probiotic-designated isolates remain limited [247]. Together, these findings suggest that *S. thermophilus* is best understood not as a uniform probiotic entity, but as a collection of functionally diverse strains whose clinical potential remains promising yet insufficiently validated in humans.

#### 4.4.4. *Lactococcus lactis* W19 and W58

At present, comparable strain-specific syntheses for *Lactococcus lactis* W19 and *Lactococcus lactis* W58 cannot be provided because no eligible mechanistic or clinical studies were identified in the current evidence set.

#### 4.4.5. *Escherichia coli Nissle* 1917 (EcN)

Across randomized trials, meta-analyses, and mechanistic studies, *Escherichia coli* Nissle 1917 emerges as one of the best-characterized non-lactic probiotic strains, with its strongest human evidence concentrated in ulcerative colitis maintenance rather than remission induction. In patients with quiescent ulcerative colitis, EcN has shown efficacy comparable to mesalazine for maintaining remission, supporting its role as a strain-specific therapeutic alternative in selected settings [248,249]. Mechanistically, EcN reinforces epithelial barrier integrity through redistribution of tight-junction proteins, limits intestinal hyperpermeability, modulates T-cell cycling through Toll-like receptor 2-dependent signaling, induces human beta-defensin-2, and suppresses enteric pathogens through microcin production and interference with bacterial invasion [250,251,252,253,254,255]. Preclinical work further links EcN to modulation of inflammatory signaling, restoration of SCFA-related barrier function, and reduced susceptibility to neuroinflammatory disease [256,257,258]. However, these benefits are counterbalanced by an unresolved safety paradox: EcN harbors the *pks* island encoding colibactin, and experimental data suggest that its genotoxic activity may be mechanistically intertwined with at least part of its probiotic effect [259,260]. Additional studies indicate dose dependence and strong host-context dependence, with protection at lower exposures but possible harm at higher doses and impaired safety in microbiota-depleted or immunocompromised hosts [261,262]. Together, these findings position EcN as a clinically relevant and mechanistically sophisticated probiotic for ulcerative colitis maintenance, while underscoring that long-term use should be interpreted through a strain-specific risk-benefit lens rather than generalized probiotic assumptions.

#### 4.4.6. *Akkermansia muciniphila*

Across metabolic, inflammatory, and mechanistic studies, *Akkermansia muciniphila* emerges as a high-potential next-generation probiotic whose effects are strongly shaped by strain identity, host context, and formulation. The most compelling human signal comes from a proof-of-concept randomized trial in overweight and obese insulin-resistant adults, in which pasteurized *A. muciniphila* improved insulin sensitivity, lowered insulinemia, and reduced total cholesterol, with additional trends toward lower body weight and fat mass [263]. Preclinical studies extend this promise to colitis and barrier dysfunction but also expose marked strain heterogeneity: some strains attenuate DSS-induced inflammation, whereas others worsen disease, emphasizing that therapeutic inference at the species level is unreliable [264]. Mechanistically, *A. muciniphila* acts at the mucus-epithelial interface by strengthening intestinal barrier function, altering SCFA-linked mucosal signaling, activating NLRP3-dependent pathways, and promoting dendritic-cell retinoic acid synthesis that enhances IL-22-mediated mucosal protection [265,266,267]. At the same time, emerging literature suggests that the same organism can behave as a pathobiont in specific inflammatory or tumor-promoting contexts, reinforcing the need for genome-level strain selection and formulation-specific validation [268]. Together, these data position *A. muciniphila* as a promising but high-precision candidate for metabolic and inflammatory disorders, whose translation depends on careful distinction between beneficial, neutral, and potentially harmful strains.

#### 4.4.7. *Clostridium butyricum* MIYAIRI 588 (CBM588)

Across a smaller but mechanistically coherent evidence base, *Clostridium butyricum* MIYAIRI 588 emerges as a spore-forming probiotic with particular relevance to post-surgical inflammatory disease and microbiota-metabolic recovery. Its clearest clinical signal derives from a small randomized placebo-controlled study in patients with ulcerative colitis after ileal pouch-anal anastomosis, where CBM588 reduced the incidence of pouchitis and favorably altered microbiota composition over long-term follow-up [269]. Mechanistic work indicates that these effects are mediated, at least in part, through induction of intestinal IL-10-producing macrophages via TLR2/MyD88-dependent pathways, rather than through classical Treg-dominant immunoregulation [49]. Additional preclinical studies show that CBM588 reshapes the gut microbiome, supports colonic tissue homeostasis, protects intestinal barrier function during antibiotic-associated dysbiosis, and modulates broader gut metabolic outputs [270,271,272]. This functional profile may extend beyond the gut-immune axis, as experimental work in high-fat diet models suggests beneficial effects on hepatic lipid metabolism and non-alcoholic fatty liver disease [273]. Together, these findings suggest that CBM588 is a mechanistically distinctive and clinically promising strain, although its translational strength remains limited by the small size of the human evidence base and the relative absence of large confirmatory trials.

#### 4.4.8. *Enterococcus faecium* SF68

At present, a comparable strain-specific synthesis for *Enterococcus faecium* SF68 cannot be provided because robust eligible studies were not identified in the current evidence set.

**Figure 2:**
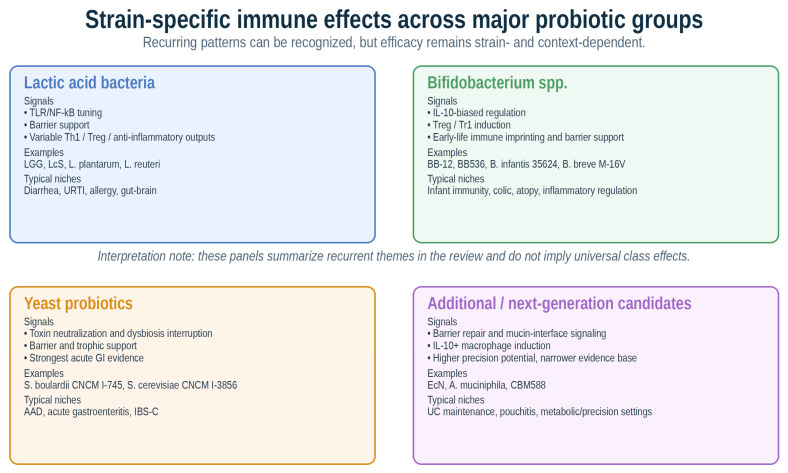
Strain-specific immune effects across major probiotic groups. Recurring patterns can be recognized, but efficacy remains strain-and context-dependent.

Viewed across the strain-level profiles, the evidence is not evenly distributed. The most consistent human signals cluster around narrow, clinically defined outcomes—such as prevention of antibiotic-associated or acute infectious diarrhea, selected oral/upper-airway endpoints, ulcerative colitis maintenance or pouchitis prevention, and constipation-predominant IBS—whereas broader claims of generalized immune enhancement or systemic anti-inflammatory benefit are supported mainly by preclinical, small, or heterogeneous studies [89,187,188,189,201,202,220,221,222,223,224,248,249,269,274]. This pattern suggests that immune effects should be interpreted as mechanism–endpoint pairings for defined strains rather than as transferable properties of a genus or species (Figure 2).

## 5. Clinical Translation: Probiotics in Immune-Related Diseases

### 5.1. Infectious Diseases

Clinical evidence provides relatively consistent support for the use of selected probiotic strains in the prevention of infectious diseases, particularly antibiotic-associated diarrhea (AAD) and upper respiratory tract infections (URTI), although effects are strongly strain-dependent. Meta-analyses of randomized controlled trials demonstrate that probiotics significantly reduce the incidence of AAD in both pediatric and adult populations, with relative risk reductions ranging from 0.46 to 0.58 and numbers needed to treat between 10 and 13 [275,276]. Importantly, these effects are not uniform across strains. For example, *Saccharomyces boulardii* CNCM I-745 showed consistent efficacy across multiple settings (RR 0.43, 95% CI 0.31–0.58), whereas only selected *Lactobacillus* strains, such as *L. casei* DN114001 (RR 0.32) and *L. reuteri* ATCC 55730 (RR 0.35), demonstrated comparable benefits, while others failed to show significant effects [158,277].

In Clostridioides difficile-associated diarrhea (CDAD), strain specificity is even more pronounced. *S. boulardii* remains the only consistently effective strain for prevention (RR ~0.40–0.59), whereas Lactobacillus strains that are effective in AAD prevention do not confer protection against CDAD, highlighting distinct mechanisms of action depending on pathogen context [278,279]. However, evidence for secondary prevention of CDAD recurrence remains inconclusive, with no probiotic strain consistently demonstrating benefit [280].

For URTI, probiotics demonstrate modest but reproducible effects, including reduced incidence (OR 0.53) and shortened duration of infection by approximately 1.9 days [281]. These effects are particularly evident in children and high-risk populations. For instance, a randomized trial using *Bifidobacterium animalis* subsp. *lactis* and *Lactiplantibacillus plantarum* showed a 54.6% reduction in recurrent respiratory infections, along with decreased risk of pneumonia and bronchitis [282]. Similarly, multi-strain formulations containing *L. helveticus*, *B. infantis*, and *B. bifidum* significantly reduced infection frequency, duration, and antibiotic use in pediatric cohorts [283]. In contrast, evidence in older adults is less consistent, with variability in strain efficacy and overall lower-quality data [284].

Additional infectious indications show mixed results. Probiotics improve *Helicobacter pylori* eradication rates when used as adjunct therapy (RR 1.09), with *Bifidobacterium* strains showing the highest efficacy (RR 1.23), followed by *Lactobacillus* (RR 1.18) and *Saccharomyces* (RR 1.07) [285]. In urinary tract infections, overall effects are not significant, although strain selection and study design appear critical determinants of outcome [286]. In critically ill patients, high-dose multi-strain formulations administered for at least 14 days significantly reduce ICU-acquired infections, whereas single-strain interventions such as *L. rhamnosus* GG show no benefit, further emphasizing dose- and formulation-dependence [287].

### 5.2. Allergic Diseases

Probiotic trials show comparatively consistent clinical efficacy signals in allergic diseases in the context of atopic dermatitis (AD), although effects remain strongly dependent on strain, disease severity, and treatment duration. Meta-analyses show significant reductions in disease severity, with SCORAD decreases ranging from −5.7 in children to −12.3 in adults [288,289,290]. However, these effects are not uniform across strains. *Limosilactobacillus fermentum*, *Lactobacillus salivarius*, and *L. acidophilus* show the strongest therapeutic effects, whereas other strains within the same genera may be ineffective [289,291].

Notably, the magnitude of clinical improvement varies with disease phenotype and duration of therapy. Longer interventions (>3 months) and younger patient age are associated with greater benefit, while effects on immunological markers such as IgE and eosinophils are inconsistent, suggesting that probiotic effects in AD may involve alternative immunomodulatory pathways beyond classical IgE-mediated responses [289,292].

For the prevention of atopic dermatitis, evidence is comparatively more consistent. Prenatal and early-life probiotic supplementation reduces disease incidence (RR ~0.78–0.79), with particularly strong effects observed for *Lactobacillus rhamnosus* GG, which reduces eczema risk by up to 50% in high-risk infants [293,294,295]. However, mixed-strain formulations do not consistently outperform single strains, indicating that increasing microbial diversity does not necessarily translate into improved clinical efficacy [296].

In contrast, evidence for asthma is inconsistent. Meta-analyses show no significant overall reduction in asthma risk (RR 0.86), although subgroup analysis indicates that *L. rhamnosus* GG may confer modest protection (RR 0.75) [297]. Similarly, while probiotics reduce IgE levels and atopic sensitization in some studies, effects on clinical asthma outcomes remain limited and heterogeneous [298].

For allergic rhinitis, probiotics demonstrate moderate efficacy, with reductions in symptom scores and improvements in quality of life, particularly for *L. paracasei* strains [299]. However, comparative analyses indicate substantial variability between probiotic species, with Saccharomyces and mixed formulations sometimes outperforming Lactobacillus in specific endpoints, further supporting the principle of strain- and indication-specific efficacy [300].

### 5.3. Autoimmune and Inflammatory Diseases

In autoimmune and inflammatory conditions, probiotic efficacy is highly disease-specific, with clear benefits observed in ulcerative colitis (UC) but not in Crohn’s disease (CD). Meta-analyses consistently demonstrate that probiotics significantly increase remission rates in UC (OR ~2.00), particularly when used in combination with standard therapies such as 5-aminosalicylic acid [301]. Multi-strain formulations such as VSL#3 show the strongest effects, with remission rates significantly improved and relapse rates markedly reduced [302].

In pouchitis, reported efficacy appears comparatively strong, with markedly reduced recurrence rates (OR 0.03), although this conclusion remains indication-specific and dependent on the studied formulation [303]. Similarly, *Escherichia coli* Nissle 1917 demonstrates efficacy comparable to standard pharmacological therapy in maintaining UC remission, highlighting the potential for strain-specific probiotics to function as therapeutic alternatives [304].

In contrast, probiotics show no consistent benefit in Crohn’s disease. Multiple meta-analyses report no significant effect on remission induction or relapse prevention, even when using strains effective in UC, suggesting fundamental differences in disease pathophysiology and host–microbiota interactions [301,305].

Beyond IBD, probiotics demonstrate immunomodulatory effects in systemic inflammation, including reductions in TNF-α, CRP, and increases in IL-10, although heterogeneity across studies is high (I^2^ > 90%), limiting the strength of conclusions [274]. Similarly, in celiac disease, probiotics modulate immune parameters and microbiota composition but show limited impact on key clinical outcomes such as IgE levels or symptom resolution, indicating modest clinical relevance despite mechanistic plausibility [306].

### 5.4. Immunodeficiency and Immune Function Enhancement

Evidence for probiotic use in immunodeficiency is heterogeneous and depends strongly on the type of immune dysfunction. In primary immunodeficiencies, direct clinical evidence for microbiome-targeted interventions remains limited, and current discussion is centered largely on conceptual or early translational approaches, particularly in common variable immunodeficiency [307].

In contrast, specific probiotic combinations have shown reported benefits in secondary immunodeficiency states, particularly in children with recurrent infections. Randomized trials demonstrate that specific strain combinations can reduce infection recurrence by over 50%, along with improvements in microbiota stability and immune markers [282]. Similarly, multi-strain probiotics significantly reduce the frequency and severity of recurrent respiratory infections in pediatric populations, suggesting clinical benefit in the studied pediatric cohorts [283].

In older adults, probiotics improve vaccine responses, with meta-analyses demonstrating increased seroconversion rates for influenza vaccines (OR up to 2.74), suggesting improved adaptive immune responsiveness [308]. However, results vary depending on strain, timing, and vaccine type, and not all studies demonstrate significant benefit [14].

In immunocompromised clinical settings, such as ICU patients, probiotics reduce infection rates only when administered at sufficiently high doses (≥5 × 10^9^ CFU/day) and for adequate duration (≥14 days), highlighting a clear dose–response relationship [287]. In contrast, single-strain interventions are often ineffective in this population.

In patients with HIV, probiotics produce modest improvements in immune parameters such as CD4/CD8 ratio and inflammatory markers, but the clinical significance of these changes remains uncertain [309]. Similarly, in preterm infants, only selected strain combinations demonstrate benefits in reducing mortality, sepsis, and necrotizing enterocolitis, with limited overlap between effective strains across outcomes [310].

### 5.5. Synthesis

Across all disease domains, a consistent pattern emerges: probiotic efficacy is both strain-specific and disease-specific, and cannot be generalized across species or indications [158]. Strains effective in one condition often fail in another, even within related disease categories. For example, probiotics that are highly effective in ulcerative colitis show no benefit in Crohn’s disease, while strains effective in AAD prevention do not prevent CDAD recurrence [278,301].

Moreover, clinical outcomes depend not only on strain selection but also on host factors, disease severity, timing of administration, and treatment duration. Dose thresholds (e.g., ≥5 × 10^9^ CFU/day) and treatment duration (>3 months for atopic dermatitis) emerge as critical determinants of efficacy in several conditions [287,292].

Across clinical domains, interpretation is limited by heterogeneity in sample size, study design, strain identity, dosage, formulation, treatment duration, baseline microbiota, age, and disease severity. Consequently, apparent efficacy signals should be interpreted as indication- and strain-specific rather than as evidence for broad immune enhancement.

Accordingly, the clinical evidence should be read as a map of where reproducible strain–indication signals have emerged, not as proof that probiotics exert broadly reliable immune benefits across clinical populations; in high-heterogeneity domains, positive findings remain hypothesis-generating until replicated with standardized strain identity, dose, formulation, and endpoints [158,274,275,276,277,278,279,280,281,282,283,284,285,286,287,288,289,290,291,292,293,294,295,296,297,298,299,300,301,302,303,304,305,306,307,308,309,310] (Table 2).

## 6. Emerging Concepts in Immune-Modulating Probiotics

### 6.1. Next-Generation Probiotics

Among next-generation probiotics, *Akkermansia muciniphila* currently has the strongest translational profile, whereas *Faecalibacterium prausnitzii* offers equally compelling mechanistic rationale but remains more difficult to develop clinically because of major cultivation and formulation barriers [311,312,313,314,315,316]. *A. muciniphila* colonizes the mucus layer and exerts immunomodulatory effects through multiple complementary mechanisms, including acetate and propionate production, reinforcement of tight-junction proteins such as ZO-1, occludin, and claudins, increased mucin turnover, and signaling mediated by outer-membrane components including Amuc_1100 [311,312,317,318,319,320]. In addition, a distinctive membrane phospholipid from *A. muciniphila* has been shown to engage non-canonical TLR2-TLR1 signaling and induce homeostatic immune responses, providing a mechanistically attractive explanation for its ability to recalibrate inflammatory thresholds rather than simply suppress immunity [5,317,321]. Across review-level sources, these pathways are linked to expansion of regulatory T cells, reduced gut permeability, increased IL-10 signaling, and attenuation of pro-inflammatory mediators such as IL-6, IL-1β, and IL-17, although context-dependent increases in TNF-α and IFN-γ have also been described, indicating that *A. muciniphila* is not uniformly anti-inflammatory in every disease setting [313,314,317,318,320,322].

Human clinical evidence for *A. muciniphila* remains limited but is notably more developed than for most other next-generation candidates. The most frequently cited intervention is a randomized, double-blind, placebo-controlled trial in 32 overweight or obese adults, in which daily supplementation with 10^10^ CFU of live or pasteurized *A. muciniphila* for three months improved insulin sensitivity by approximately 30%, while also reducing insulinemia, total cholesterol, and selected anthropometric parameters [311,312,319]. Importantly, pasteurized preparations performed at least as well as live organisms in several outcomes, supporting the concept that viability is not always required for clinical benefit and helping explain why pasteurized *A. muciniphila* has progressed further along the regulatory pathway, including EFSA novel food acceptance [312,318,319,320,323]. Beyond metabolism, oncology-focused evidence is especially intriguing. A meta-analysis summarized in the uploaded report, encompassing 38 studies and 5642 patients, found that enrichment of *A. muciniphila* together with *F. prausnitzii* and *Bifidobacterium longum* was associated with improved overall survival (HR 0.62) and progression-free survival (HR 0.69) during immune checkpoint inhibitor therapy, while fecal microbiota transplantation from responders increased objective response rates [5,324]. At the same time, review-level evidence also highlights a more cautious interpretation in intestinal inflammation, because *A. muciniphila* may be beneficial in some IBD-associated contexts yet potentially pro-inflammatory in others, underscoring the need for phenotype-specific deployment rather than indiscriminate supplementation [313,314,315,318,322].

*Faecalibacterium prausnitzii* complements *A. muciniphila* by representing a highly abundant butyrate-producing commensal with particularly strong anti-inflammatory credentials [313,314,315,316]. Mechanistically, *F. prausnitzii* promotes regulatory T-cell expansion, suppresses NF-κB-driven inflammatory signaling, reduces IL-12, IFN-γ, IL-1β, and TNF-α, and increases IL-10, thereby supporting a more tolerogenic mucosal environment [313,314,315,316,325]. In contrast to many conventional probiotics, part of its activity has been attributed not only to butyrate itself but also to specific microbial anti-inflammatory molecules (MAM peptides), which appear to add a distinct layer of host signaling control beyond generic SCFA production [314,315,316,325]. These properties make *F. prausnitzii* particularly attractive for inflammatory bowel disease and autoimmune disorders, where disruption of barrier integrity and dysregulation of the Treg/Th17 axis are central to disease biology [314,315,316,324,325]. However, despite strong mechanistic support and early-phase clinical development in IBD, the human evidence base for *F. prausnitzii* remains far smaller than for *A. muciniphila*, and its extreme oxygen sensitivity continues to complicate cultivation, storage, delivery, and large-scale commercialization [314,315,316,324,325].

Taken together, the next-generation probiotic field already shows a clear hierarchy of maturity. *A. muciniphila* is the most clinically advanced and mechanistically diversified candidate, while *F. prausnitzii* remains one of the most biologically compelling but technically constrained organisms in the pipeline [311,312,313,314,315,316,323,325]. This asymmetry is important because it illustrates a broader principle: future immune-modulating probiotics will likely be selected not for taxonomic novelty alone, but for a combination of defined mechanism, manufacturability, safety, and disease-context specificity [313,316,323,325].

### 6.2. Yeast Probiotics

Within emerging immune-modulating strategies, *Saccharomyces boulardii* occupies a special position because it is a non-bacterial probiotic platform with a long clinical history in gastrointestinal disorders, yet comparatively limited evidence in dedicated immune-modulation frameworks [326,327]. In the immune-focused literature captured in the uploaded report, *S. boulardii* is characterized less by broad baseline immunostimulation and more by a relatively restrained interaction with the healthy mucosal immune system [326]. In particular, experimental work in healthy intestinal settings showed limited association with Peyer’s patches, minimal direct immune-cell engagement, and only modest systemic humoral responses, suggesting that *S. boulardii* may perturb immune homeostasis less aggressively than many bacterial probiotics [326]. This feature can be interpreted in two opposing ways: on the one hand, it may limit strong intrinsic immunomodulatory effects; on the other, it may make yeast-based systems attractive where controlled or low-noise immune interaction is desirable [326].

The most forward-looking application of *S. boulardii* in this literature is its use as an engineered therapeutic chassis rather than as a conventional probiotic in the narrow sense [327]. A yeast-based oral platform designed to secrete immune checkpoint inhibitor–related biologics reduced intestinal tumor burden in colorectal cancer models refractory to checkpoint blockade and altered both immune-cell composition and microbiome structure [327]. This positions *S. boulardii* less as a replacement for bacterial next-generation probiotics and more as a customizable delivery vehicle for targeted gastrointestinal immunotherapy [327]. Such a role is conceptually important for an article on immune function because it expands the definition of a probiotic strategy from “live organism conferring benefit” to “programmable microbial platform capable of delivering an immunologically active payload in situ” [327].

Nevertheless, yeast-based approaches bring distinct safety questions. Unlike bacterial probiotics, *S. boulardii* carries a theoretical and clinically relevant risk of fungemia, especially in critically ill or severely immunocompromised patients, and these concerns likely contribute to the relatively limited representation of yeast probiotics in the immune-focused emerging literature [326,327]. Accordingly, although *S. boulardii* remains indispensable in the broader probiotic field, its role in next-generation immune modulation appears likely to develop primarily through engineered or precision-delivery applications rather than through conventional broad-spectrum supplementation [326,327].

### 6.3. Postbiotics

Postbiotics have emerged as a compelling extension of probiotic science because they seek to preserve the beneficial biological signals of microbes while reducing the practical and safety liabilities associated with live organisms [328,329,330]. Across the reviewed literature, postbiotics are broadly conceptualized as inanimate microorganisms, structural components, or microbially derived metabolites capable of modulating host physiology and immunity without requiring sustained viability or colonization [328,329,330]. This framework is especially attractive in immune medicine, where translocation risk, loss of viability during storage, and inconsistent engraftment can all limit the performance of live biotherapeutics [328,329,330].

Among postbiotics, short-chain fatty acids remain the best characterized and the most mechanistically mature. Butyrate, propionate, and acetate act through dual signaling logic: they activate G protein-coupled receptors such as GPR41, GPR43, and GPR109A, and they inhibit histone deacetylases, thereby linking microbial metabolism directly to host transcriptional and immunological programs [16,331,332]. Through these pathways, SCFAs suppress NF-κB signaling, reduce IL-1β, TNF-α, and IL-6, increase IL-10, promote regulatory T-cell differentiation, suppress Th17 polarization, and reshape macrophage behavior toward less inflammatory phenotypes [16,331,332]. Their role is not confined to immune cells. SCFAs also reinforce epithelial barrier integrity by supporting colonocyte metabolism, stimulating mucus production, and increasing tight-junction expression, which helps explain why metabolite-centered approaches may simultaneously reduce mucosal leakiness and systemic inflammation [16,331,332,333].

Accordingly, the postbiotic landscape should not be restricted to acetate, propionate, and butyrate. Less abundant SCFAs, particularly valeric acid, may become relevant candidates for metabolite-centered interventions if their proposed epigenetic and immunological effects are confirmed in controlled human studies [33].

Translational evidence for postbiotics is promising but still uneven. Direct trials of purified SCFA supplementation remain limited, yet several clinically relevant human datasets suggest that microbially generated postbiotic exposure has measurable systemic effects. Fermented dairy interventions that increase exposure to beneficial microbial metabolites have produced 4–12 week improvements in fasting glucose, LDL cholesterol, and systemic CRP in cardiometabolic-risk populations, supporting the idea that diet–microbiome–metabolite interactions can be therapeutically harnessed even when isolated metabolite therapy is not yet standardized [333]. A 28-day clinical trial of a three-strain postbiotic mix also reported improvement in immune-system function after oral intake, although the trial base remains too small to support strong generalization [334]. In more complex immune settings, protective intestinal metabolite patterns including SCFAs and bile-acid derivatives have been associated with better outcomes after allogeneic stem cell transplantation, suggesting that postbiotic signatures may function both as biomarkers and as future therapeutic targets [335]. In inflammatory bowel disease, review-level evidence indicates that postbiotic and paraprobiotic strategies can reduce inflammatory signaling, improve epithelial homeostasis, and potentially complement live biotherapeutics, although most data remain preclinical or early translational [329,336,337].

The postbiotic repertoire extends well beyond SCFAs. Secondary bile acids influence immune function through FXR and TGR5 signaling, thereby modulating macrophage polarization, intestinal barrier function, and inflammatory tone [16,329,337]. Tryptophan-derived microbial metabolites activate AhR and support IL-22-associated mucosal defense, while also influencing T-cell differentiation and inflammatory balance [16,329]. Other molecules such as inosine and microbially modified purines have been implicated in antitumor immunity, especially through effects on T-cell activation and checkpoint inhibitor responsiveness [16,331,338]. Structural postbiotics are equally relevant. Surface proteins such as Amuc_1100 and defined bacterial polysaccharides can activate pattern-recognition pathways or induce regulatory T-cell responses without requiring administration of live bacteria, thereby offering a more standardized route to immunological intervention [317,320,322,330].

One of the strongest arguments in favor of postbiotics is their safety and stability profile. Because the administered product is non-viable, the risk of bloodstream dissemination, opportunistic infection, and viability loss is substantially reduced, which is especially attractive for immunocompromised populations and for applications requiring long shelf life or ambient storage [328,329,330,336,337]. However, this conceptual advantage should not obscure the current evidence gap. The field still lacks standardized nomenclature, harmonized manufacturing criteria, and adequately powered randomized trials directly comparing postbiotics with conventional live probiotics or placebo in immune-mediated diseases [328,329,330,333,337]. Thus, postbiotics are no longer purely speculative, but they remain translationally earlier than their mechanistic sophistication might suggest.

### 6.4. Precision Probiotics

Precision probiotics represent the logical next step once strain specificity, host heterogeneity, and context-dependent responses are taken seriously [4,339,340,341]. Rather than assuming that a single formulation can be broadly applied across patients, precision approaches integrate microbiome profiling, host characteristics, disease phenotype, and increasingly machine-learning tools to select the most appropriate microbial strain, consortium, or metabolite intervention for a given individual [4,339,340,341]. In practical terms, this means moving from empirical supplementation toward microbiome-based treatment strategies that are guided by metagenomics, metabolomics, metatranscriptomics, and other multi-omics platforms capable of defining both baseline ecological deficits and likely therapeutic response [4,339,340,341].

Host context is also shaped by diet, genetic background, and age. Diet determines the availability of fermentable substrates and therefore influences whether a strain can generate immunologically active metabolites such as SCFAs or indole derivatives [15,16]. Host genetic variation may modify epithelial barrier function, innate immune sensing, mucosal glycosylation, and antigen-presentation pathways, thereby altering responsiveness to the same probiotic strain. Age adds a further layer of heterogeneity: in infants, probiotic effects occur during immune education and microbiome assembly, whereas in older adults they occur against a background of immunosenescence, inflammaging, reduced microbial diversity, and altered vaccine responsiveness. These factors help explain why identical strains may produce divergent immune effects across populations [4,14,147,148,163,166,310,339,340,341].

Cancer immunotherapy is currently the clearest showcase for this paradigm. The accumulated literature indicates that microbiome diversity and enrichment of specific taxa—especially *A. muciniphila*, *F. prausnitzii*, and *B. longum*—are associated with superior response to immune checkpoint inhibitors, better survival, and more favorable systemic immune activation [5,314,342,343]. These findings have catalyzed a range of targeted strategies, from responder-derived fecal microbiota transfer to engineered microbial platforms delivering checkpoint-modulating molecules or neoantigens [5,314,342,343,344,345,346]. In preclinical systems, engineered food-borne probiotics and probiotic delivery vectors have been used to produce PD-L1-targeting nanobodies, stimulate type I immune responses, enhance CD8^+^ T-cell infiltration, and improve tumor control, thereby demonstrating how synthetic biology can transform microbes into programmable immune therapeutics [342,344,345,346]. Although these results remain largely preclinical, they move the field well beyond classical probiotic supplementation and into the territory of living precision medicines [342,344,345,346].

In inflammatory bowel disease and autoimmune disorders, precision strategies are evolving around defined bacterial consortia, disease-endotype matching, and microbiome-guided strain selection [4,324,339,340,341]. Review-level evidence suggests that patient stratification based on baseline dysbiosis, inflammatory phenotype, and functional microbial deficits may improve the likelihood of response to targeted microbial interventions, including consortia enriched for anti-inflammatory anaerobes [4,324]. Similar logic is being extended to rheumatoid arthritis, where microbiome-driven shifts in the Th17/Treg axis and mucosal barrier status are increasingly viewed as actionable targets for tailored probiotic or metabolite-based therapy [4]. The central implication is that future efficacy may depend as much on selecting the right patient as on selecting the right strain [4,339,340,341].

Despite this promise, precision probiotics remain more conceptually advanced than clinically validated. Most of the evidence still comes from observational associations, review-level syntheses, small early-phase studies, or preclinical models, and there are few large superiority trials demonstrating that microbiome-guided selection materially outperforms well-chosen empirical supplementation [4,339,340,341,342,343,344,345,346]. Additional barriers include the cost of profiling, lack of standardized response algorithms, population-specific microbiome variation, and major regulatory challenges for engineered live therapeutics [4,339,340,341,342,343,344,345,346]. Even so, the overall direction of travel is clear: precision probiotics are redefining the field from a supplement-centered model to a systems-medicine model in which microbial therapeutics are matched to host ecology, immune context, and treatment goal [4,339,340,341,342,343,344,345,346].

Taken together, emerging immune-modulating probiotic strategies indicate that the field is moving beyond conventional *Lactobacillus*/*Bifidobacterium* paradigms toward a broader therapeutic spectrum that includes next-generation anaerobes, yeast-based delivery platforms, metabolite-centered postbiotics, and microbiome-guided precision interventions [311,312,313,314,315,326,328,339]. Their shared strength lies in targeting barrier integrity, metabolite-receptor signaling, and Treg/Th17 balance with greater mechanistic specificity than traditional formulations, but this same sophistication brings new translational challenges, including strain-level heterogeneity, manufacturing constraints, safety monitoring, and the need for validated patient-selection frameworks [4,314,315,316,324,328,329,330,339,340,341,342,343,344,345,346]. As a result, these approaches should currently be viewed not as replacements for conventional probiotics, but as a rapidly expanding immunological toolkit whose clinical value will depend on rigorous trial design and increasingly precise deployment [4,314,315,316,328,339,340,341,342,343,344,345,346].

## 7. Safety and Immunological Risks

Safety in probiotic interventions should be interpreted as an immunological and host-contextual variable rather than a purely toxicological endpoint, because probiotic effects are demonstrably strain-specific, compartment-dependent, and methodologically heterogeneous across the available literature [3,5,7,14]. The current evidence base spans infancy, healthy adulthood, obesity-related inflammation, oncology settings, and experimentally immunosuppressed models, but these populations are biologically non-equivalent and cannot support unrestricted cross-population extrapolation [1,5,7,347,348]. Accordingly, the assumption that probiotics constitute a uniformly safe class is not supported at the level of systemic immunity, where dose, viability, formulation, host age, baseline microbiota, and immune status remain major effect modifiers [3,7,13,14].

This limitation is particularly consequential in immunocompromised patients, for whom direct safety evidence remains sparse and uneven [3,5,7,347]. In oncology, most probiotic-associated immune benefits are derived from observational datasets, mechanistic inference, or preclinical augmentation of antitumor responses rather than from adequately powered, safety-oriented randomized trials [5]. Likewise, cyclophosphamide-immunosuppressed animal models show that selected strains can restore NK-cell activity, immunoglobulin production, and cytokine balance, but these findings demonstrate immune potentiation rather than validated clinical safety in immunologically fragile humans [347]. More broadly, the field is weakened by high risk of bias in many primary studies, incomplete methodological robustness, sparse human intervention data on systemic immune modulation, and non-standardized products and dosing regimens, all of which reduce confidence in safety extrapolation to clinically vulnerable hosts [3,7,13,14].

Bloodstream complications require equally cautious interpretation [3,5,7,13]. Within the present evidence synthesis, fungemia and bacteremia were not consistently captured as dedicated, strain-resolved safety endpoints, whereas efficacy and mechanistic immune readouts were much more prominently reported [3,5,7,13]. Their relative underrepresentation should therefore be read as incomplete surveillance rather than proof of absent bloodstream risk, especially in hosts with impaired barrier integrity, altered immune competence, or complex comorbidity profiles [3,5,13]. Future trials should consequently treat fungemia and bacteremia as prespecified safety outcomes and report them at the strain and formulation level rather than subsuming them under generic statements of probiotic tolerability [5,7,13].

A further safety issue is the potential for horizontal gene transfer. Although clinically documented transfer events from administered probiotic strains appear uncommon, probiotic candidates may carry plasmids, transposons, or other mobile genetic elements that could theoretically disseminate antimicrobial-resistance or virulence-associated determinants within dense microbial communities [117,237]. This risk is particularly relevant in vulnerable hosts, in whom prolonged colonization, antibiotic exposure, impaired barrier integrity, or indwelling devices may increase ecological opportunities for gene exchange. For this reason, strain-level safety assessment should include whole-genome screening for transferable antimicrobial-resistance genes, virulence-associated loci, and mobile genetic elements, rather than relying only on historical species-level safety status [3,13].

A second and conceptually distinct risk concerns immunological miscalibration, namely whether a probiotic induces appropriate immunoregulation or instead drives an excessive, mistimed, or tissue-inappropriate immune signal [3,9,11,14,79,348]. In the same broader evidence base, some strains promote IL-10- and TGF-β-associated tolerance, enhance regulatory T-cell programs, or support early-life immune imprinting [1,9,11]. Other strains or formulations, however, can bias immunity toward Th1-, Th2-, or Th17-associated states, and these effects may diverge substantially between the liver, spleen, peripheral blood, and mucosal compartments [11,79,348]. Fong et al. demonstrated that single strains and multi-strain mixtures generate distinct and non-additive immune profiles across tissues, indicating that biological behavior cannot be inferred from product composition alone [348]. The vaccine literature provides a clinically relevant parallel, because beneficial effects are inconsistent overall and maternal probiotic exposure during pregnancy has in at least one context been associated with reduced infant vaccine responses, underscoring the importance of timing, route of exposure, and developmental stage [14]. Thus, the principal immunological hazard is not simply lack of efficacy, but the possibility that a probiotic may elicit the wrong immune program in the wrong host at the wrong time [1,9,11,14,79,348].

Taken together, the available literature supports a strain-resolved safety framework rather than a class-based presumption of harmlessness [3,13]. For immunocompromised hosts in particular, probiotic use should be grounded in population-specific validation, formulation fidelity, and active surveillance for systemic infectious and immunological adverse events [3,5,7,13]. In this context, the concept of targeted probiotics—defined strains with explicit mechanistic rationale, reproducible manufacture, and validation in the intended host population—appears to be as critical for safety as it is for efficacy [13].

## 8. Limitations of Current Evidence

Although the available synthesis focuses primarily on safety and immunological risk rather than direct immune-efficacy endpoints, it still highlights several methodological weaknesses that substantially limit the interpretation of probiotics in relation to immune function.

1. **Small sample sizes and predominance of low-level evidence.** Much of the current literature on clinically relevant immune-related probiotic effects is derived from single case reports, small case series, and case-based systematic reviews, whereas robust randomized evidence remains limited. The strongest controlled evidence cited in the available synthesis is a single large multicenter ICU trial, while pediatric and neonatal reviews summarize relatively small numbers of confirmed invasive cases rather than large, standardized cohorts [15,18,19,20]. This limits statistical power, weakens causal inference, and makes it difficult to determine the true frequency and magnitude of clinically meaningful immune-related benefits or harms [15,16,18,19].
2. **Marked heterogeneity across studies.** The evidence base is highly heterogeneous with respect to study design, patient population, clinical setting, probiotic organism, and outcome definition. Available data combine preterm neonates, critically ill adults, immunocompromised patients, and otherwise healthy children, even though these groups differ substantially in baseline immune status and risk of microbial translocation. Outcomes also vary widely, ranging from mild gastrointestinal symptoms to bacteremia, fungemia, sepsis, and sterile-site recovery of probiotic organisms [15,16,17,18,19,21]. This heterogeneity limits comparability between studies and makes broad clinical generalization inappropriate, because apparent safety or harm may depend more on host context than on the probiotic itself [16,17,19].
3. **Insufficient strain-specific analysis.** A major limitation of the field is the incomplete separation of strain-level effects. Although some reports used molecular typing to confirm probiotic-derived infection, broader reviews often pool different genera, species, commercial products, and formulations into a single analytic category [16,18,19]. This is methodologically problematic because probiotic effects on immune regulation are not class effects. Clinically, it means that findings observed for one strain cannot be reliably extrapolated to another, even within the same species, which weakens both efficacy interpretation and safety assessment [15,16,18,19].
4. **Substantial variability in dose, formulation, and duration.** The published evidence includes major differences in administered dose, formulation, route, and exposure time. For example, *Lacticaseibacillus rhamnosus* GG was given at 1 × 10^10^ CFU twice daily in critically ill adults [15], whereas *Saccharomyces boulardii* was reported at 5 × 10^9^ CFU/day [22], in lyophilized 250 mg three-times-daily regimens [23], and *L. rhamnosus* GG at 3 × 10^9^ CFU/day for up to 6 weeks in very-low-birth-weight infants [21]. Time to adverse-event onset also varied substantially, with *Saccharomyces* fungemia occurring after a median of 10 days but across a wide range of 4–300 days [20]. Such variability obscures dose–response relationships and prevents clinically meaningful conclusions about optimal dosing, treatment duration, or thresholds of risk [15,20,21,22,23].
5. **Likely publication and reporting bias.** The field is also vulnerable to publication bias, because rare and dramatic complications are more likely to be reported than uneventful exposures. At the same time, randomized trials conducted in lower-risk populations may be underpowered to detect rare immune-related harms or may not document adverse events with sufficient granularity. Importantly, Hempel et al. concluded that probiotic interventions and adverse events were poorly documented, while other reviews reported no increase in serious adverse events in healthier populations [16,17]. As a result, the literature may simultaneously overemphasize unusual severe complications and underestimate clinically important risks in vulnerable patients, thereby complicating bedside interpretation [15,16,17,19,20].

Overall, these limitations mean that the current evidence base does not support broad, class-level conclusions regarding probiotics and immune function. Clinical interpretation should therefore remain strain-specific, population-specific, and cautious, particularly in immunocompromised, critically ill, or mucosal-barrier-disrupted patients [15,16,18,19,20,21]. These limitations apply not only to safety assessment but also to efficacy interpretation, because the same weaknesses that obscure rare harms also limit confidence in causal claims about immune benefit (Table 3).

## 9. Future Perspectives

The future of probiotic immune modulation will likely be defined not by the simple expansion of probiotic strain catalogues, but by a transition from empirical supplementation toward precision immunomodulation. Current evidence increasingly supports the view that probiotic efficacy is context-dependent, varying with baseline microbiome composition, immune phenotype, disease subtype, and host-specific ecological constraints, which makes one-size-fits-all strategies progressively less defensible [339,349,350]. In this emerging framework, conventional probiotics, next-generation strains, engineered microbes, postbiotics, and defined microbial metabolites should be viewed as a continuum of microbiome-based immunotherapeutic platforms rather than as isolated product categories [351,352,353,354,355]. This conceptual shift is important because it reframes probiotics from broadly “beneficial” adjuncts into potentially selectable tools for targeted immune regulation.

A major near-term impact of this shift will be the development of precision immunomodulation strategies based on mechanistically informed patient stratification. Multi-omics profiling, metaproteomics, metabolomics, and AI-assisted analytics are beginning to define microbial–immune signatures that can predict treatment responsiveness and guide pathway-specific intervention [339,349,356,357,358]. Rather than prescribing probiotics generically, future approaches may aim to restore discrete immune functions—such as Treg induction, epithelial barrier reinforcement, HDAC inhibition, Th1 polarization, or metabolite-mediated checkpoint sensitization—according to the dominant biological defect in a given patient [316,355,359]. This logic is already being explored in inflammatory bowel disease, cancer immunotherapy, and psychobiotic research, where pre-treatment microbiome structure appears to influence clinical efficacy and may explain a substantial proportion of the heterogeneity observed across current trials [349,356,358,360].

Within this model, personalized probiotics represent the most direct clinical translation. In practice, this will likely require selecting strains or rational microbial consortia according to baseline dysbiosis patterns, immune tone, diet, medication exposure, and disease-specific pathophysiology rather than taxonomic familiarity alone [339,349,350]. The candidate repertoire is also expected to expand beyond conventional lactobacilli and bifidobacteria toward next-generation probiotics such as *Akkermansia muciniphila* and *Faecalibacterium prausnitzii*, which offer more focused mechanisms, including mucin-interface signaling, SCFA production, HDAC inhibition, Treg induction, and epithelial barrier support [316,352,361]. In parallel, synthetic biology is opening the possibility of programmable probiotics capable of sensing local pathological cues and delivering IL-10, IL-27, anti-TNF molecules, antioxidant enzymes, or immunoregulatory metabolites such as inosine directly at sites of disease [353,362,363,364]. This strategy may be particularly valuable in IBD and oncology, where spatially precise immune modulation could be more clinically useful than broad systemic immune stimulation [353,363,364,365].

At a broader systems level, microbiome-guided therapy is likely to become the operational framework through which these advances are implemented. In such a model, baseline microbial profiling would not simply describe dysbiosis, but actively guide therapeutic choice among conventional probiotics, next-generation strains, engineered live biotherapeutics, synbiotics, postbiotics, extracellular vesicles, and metabolite-based interventions [351,353,354,355,366]. This approach is especially attractive in cancer immunotherapy and inflammatory bowel disease, where microbial signatures are increasingly linked to treatment responsiveness, mucosal immune tone, and disease trajectory [349,358,360,365,366]. Integration of machine learning with genomic, transcriptomic, metabolomic, and immune datasets may further enable identification of responder phenotypes, longitudinal monitoring of therapeutic effects, and adaptive treatment adjustment over time [356,357,367,368]. In that sense, microbiome-guided therapy moves the field beyond descriptive microbiome science toward clinically actionable immune stratification.

An equally important future direction is the strategic diversification of microbiome-derived therapeutics. Live microbes are unlikely to remain the only—or even the optimal—format in every indication. Postbiotics, extracellular vesicles, and defined microbial metabolites offer important advantages in stability, dose precision, standardization, and safety, particularly in immunocompromised patients or in settings where microbial viability and colonization are difficult to control [351,354,365,369]. These cell-free approaches can nevertheless retain meaningful immunomodulatory activity, including M2 macrophage polarization, NF-κB modulation, epithelial barrier repair, and enhancement of vaccine or checkpoint responses [351,354,365,369]. The future therapeutic landscape will therefore likely be plural rather than exclusively strain-centric: some conditions may favor self-renewing engineered microbes capable of in situ sensing and delivery, whereas others may be better served by standardized postbiotic or metabolite-based formulations with greater pharmacologic predictability [351,353,354,355,364,369].

Despite this promise, the translational impact of these advances will depend on solving several unresolved challenges. Standardized frameworks for strain selection, validated biomarkers for patient stratification, scalable manufacturing of engineered organisms, robust biocontainment systems, and harmonized regulatory pathways for live biotherapeutic products remain major unmet needs [339,353,354,368,370]. Comparable issues apply to postbiotics, where compositional consistency, quality control, and dose–response validation are still incompletely established [354,369]. Future clinical development will therefore require multicenter trials with standardized endpoints, longitudinal microbiome and immune sampling, and explicit responder analyses rather than simple average-effect comparisons [350,360,365,366]. In practical terms, profiling-guided use of existing strains and some postbiotic platforms may reach clinical implementation sooner, whereas engineered live biotherapeutics will probably advance more slowly because of biosafety, manufacturing, and regulatory constraints [339,353,354,369,370].

Several research priorities follow from these limitations. First, mechanistic studies should identify strain-specific molecular determinants of immune activity, including surface structures, secreted metabolites, extracellular vesicles, receptor targets, and epigenetic effects. Second, experimental studies should use standardized strain authentication, genome-level safety screening, defined culture conditions, dose verification, and harmonized immune endpoints to improve reproducibility. Third, clinical trials should be adequately powered, prospectively registered, strain-specific, and designed with predefined responder analyses based on age, diet, baseline microbiota, immune phenotype, medication exposure, and disease subtype. Finally, safety monitoring should include not only conventional adverse events but also bloodstream infection, transferable antimicrobial-resistance determinants, and inappropriate immune activation in vulnerable populations.

Operationally, this agenda could be translated into three near-term study designs: (i) head-to-head trials of authenticated strains within the same indication to separate strain-specific effects from generic probiotic effects; (ii) mechanism-embedded trials that pair clinical endpoints with prespecified barrier, metabolomic, cytokine, and microbiome readouts; and (iii) stratified trials in predefined responder groups, including infants, older adults, immunocompromised patients, and individuals with defined baseline dysbiosis or inflammatory phenotypes. Such designs would help distinguish true non-response from inadequate dose, formulation, or patient selection [350,360,365,366,370].

Taken together, the future of probiotic immune modulation lies in moving from empirical supplementation to precision microbiome therapeutics. The highest-impact strategies will likely be those that combine mechanistic immune targeting, individualized product selection, and microbiome-guided monitoring within a single clinical framework [339,349,350,353,357,366,367,368]. If these scientific, technological, and regulatory barriers can be addressed, microbiome-based interventions may, for selected indications and responder groups, evolve from supportive adjuncts into components of precision medicine for immune-mediated disease.

## 10. Discussion

Overall, the evidence synthesized in this review supports a biologically plausible, multilayered model in which the gut microbiota shapes systemic immunity through microbial community structure, epithelial barrier function, and metabolite-driven signaling. Across these layers, short-chain fatty acids, bile-acid derivatives, and tryptophan metabolites repeatedly emerge as central mediators linking microbial ecology to regulatory T-cell induction, restraint of Th17-associated inflammation, myeloid-cell programming, and the maintenance of immune tolerance. Conversely, disruption of epithelial integrity converts dysbiosis into systemic inflammatory signaling by facilitating translocation of microbial products and amplification of TLR- and NF-κB-dependent pathways. This mechanistic framework provides the biological rationale for considering probiotics as immune-modulating interventions rather than merely microbiota-supportive supplements [15,16,17,24,25,26,27,28,29,30,31,32,34,35,36,37,38,39].

A second key message of the present review is that immune modulation cannot be inferred from species identity alone. Even within closely related taxa, strains differ substantially in cytokine induction profiles, regulation of NF-κB and TLR signaling, support of regulatory T-cell responses, epithelial effects, and the ability to influence clinical endpoints. This principle was consistent across lactic acid bacteria, bifidobacteria, probiotic yeasts, and non-traditional candidates such as *Escherichia coli* Nissle 1917, Akkermansia muciniphila, and Clostridium butyricum MIYAIRI 588. Accordingly, taxonomy provides only a partial guide to function, whereas strain-level characterization remains essential for both mechanistic interpretation and rational clinical use [13,41,42,43,44,45,46,47,48,49,65,66,67,68,69,70,71,72,73,74,75,76,79,156,157,158,185,187,248,263,269].

From a translational perspective, the current clinical evidence is best interpreted as selective rather than uniformly supportive. However, the strength of clinical inference remains uneven because many immune readouts are associative, trial designs are heterogeneous, and strain-level resolution is frequently incomplete. The most reproducible benefits were observed in antibiotic-associated and acute infectious diarrhea, selected upper respiratory infections, ulcerative colitis maintenance, pouchitis, and some allergic phenotypes such as atopic dermatitis. By contrast, evidence remains inconsistent in Crohn’s disease, broad asthma prevention, generalized immune enhancement, and several metabolic or systemic inflammatory indications. These differences likely reflect not only strain biology, but also host age, baseline microbiota composition, disease phenotype, dose, formulation, timing, and treatment duration. Clinical heterogeneity should therefore not be interpreted simply as failure of the probiotic concept, but as evidence that immunological benefit depends on correct alignment between strain mechanism and host context [158,274,275,276,277,278,279,280,281,282,283,284,285,286,287,288,289,290,291,292,293,294,295,296,297,298,299,300,301,302,303,304,305,306,307,308,309,310]. This reinforces the need to treat diet, host genetic background, developmental stage, and immunosenescence as modifiers of probiotic response rather than peripheral variables. Thus, even favorable clinical signals are best framed as conditional and implementation-oriented, with the strongest claims reserved for replicated strain–indication pairs.

Finally, the review indicates that efficacy cannot be separated from safety or methodological rigor. Immune modulation is clinically valuable only when it is appropriately matched to the host, which is especially relevant in immunocompromised or barrier-disrupted patients, where bloodstream infection risk, product quality, and the possibility of tissue-inappropriate immune activation remain important concerns. Many inconsistencies in the literature are also likely amplified by small trials, heterogeneous endpoints, variable dosing, inconsistent strain identification, and limited use of mechanistic immune readouts. These constraints help explain why the most promising future directions are strain-resolved and precision-oriented, including next-generation probiotics, postbiotics, and microbiome-guided interventions selected according to disease context, immune phenotype, and safety profile [3,4,5,7,13,14,311,312,313,314,315,316,317,318,319,320,321,322,323,324,325,326,327,328,329,330,339,340,341,342,343,344,345,346,347,348,349,350,351,352,353,354,355,356,357,358,359,360,361,362,363,364,365,366,367,368,369,370,371,372,373,374,375,376,377,378].

## 11. Conclusions

In conclusion, selected probiotic strains can influence systemic immunity through the gut microbiota-immune axis, but their effects are not generic properties of probiotic species. Rather, defined strains act through epithelial, metabolic, innate, and adaptive pathways in a context-dependent manner, with the clearest evidence for benefit limited to selected strains, indications, and host contexts [13,15,16,17,24,41,42,43,44,45,46,47,48,49,158,301]. At present, the field does not support class-level conclusions about probiotics as generic immune modulators.

Accordingly, the future of immune-modulating probiotics lies in mechanism-based, strain-specific, and increasingly personalized interventions. Progress will depend on rigorous trials with transparent strain identification, standardized formulations, integrated microbiome-immune readouts, and careful safety surveillance, while next-generation probiotics and postbiotics may offer more precise tools for selected indications [3,4,7,13,14,311,312,313,314,315,316,328,329,330,339,340,341,342,343,344,345,346,347,348,350,351,352,353,354,355,356,357,358,359,360,361,362,363,364,365,366,367,368,369,370].

## Figures and Tables

**Table 1:** Representative immune-modulating mechanisms are synthesized from Section 2 and Section 3.

Mechanistic Layer	Key Mediators/Signals	Main Immune Consequences	Representative Examples in the Review
Community structure and diversity	SCFA-producing anaerobes; bifidobacteria; ecological redundancy	Maintains immune homeostasis and restrains inflammatory tone	*Faecalibacterium/Roseburia-rich* communities; early-life bifidobacteria
Epithelial barrier control	Tight junctions; mucus; colonocyte metabolism; IgA-dependent containment	Limits microbial translocation and systemic immune activation	Butyrate-supported barrier integrity; B-cell/IgA control of epithelial programs
SCFA signaling	Acetate, propionate, butyrate; GPR41/43/109A; HDAC inhibition	Treg expansion; Foxp3 stabilization; Th17 restraint; antibody support	Th17/Treg rebalancing; SCFA-fueled B-cell responses
Bile-acid remodeling	Secondary bile acids; FXR/TGR5; 3-oxoLCA and isoLCA	Suppresses Th17 differentiation and tunes myeloid/liver immune programs	RORγt inhibition; CXCL16/NKT axis
Tryptophan-derived metabolites	Indole derivatives; indole-3-lactic acid; AhR signaling	Supports mucosal tolerance and restrains inflammatory monocyte/macrophage programs	*B. infantis* EVC001-associated early-life immune imprinting
Strain-level innate/adaptive reprogramming	TLR2/TLR4/NOD2; NF-κB/AP-1; IL-10/Treg axes	Redirects cytokine balance, antiviral readiness, and regulatory-cell induction	*L. paracasei*, LGG, *B. infantis* 35624, *B. breve*, EcN, CBM588

**Table 2:** Clinical translation across major disease domains is discussed in the review.

Disease Domain	Most Reproducible Signal	Examples Emphasized in the Review	Main Caveats
AAD/acute infectious diarrhea	Reduced incidence or shorter duration	*S. boulardii* CNCM I-745; selected Lactobacillus strains	Effects are strongly strain-specific
Clostridioides difficile prevention	Best support for primary prevention, not recurrence prevention	*S. boulardii* shows the clearest signal	AAD efficacy does not automatically translate here
URTI/recurrent respiratory infections	Modest reduction in incidence or duration, especially in children	Selected *B. animalis* subsp. *lactis* and multi-strain pediatric formulations	Older-adult evidence is less consistent
Atopic dermatitis/allergic disease	Severity reduction and early-life preventive signal	*L. fermentum*, *L. salivarius*, *L. acidophilus*; LGG in prevention	Marker changes are inconsistent and duration matters
Ulcerative colitis/pouchitis	Improved maintenance outcomes; very strong pouchitis signal	VSL#3-type multi-strain formulations; EcN	Benefit does not generalize to all IBD phenotypes
Crohn’s disease	No consistent remission or relapse benefit	—	Clear disease-specific divergence from ulcerative colitis
Vaccine response/recurrent infection states	Selected gains in seroconversion or recurrent infection reduction	Older-adult vaccine studies; pediatric recurrent-infection trials	Timing, dose, and strain choice remain decisive
Other systemic/metabolic indications	Mechanistic promise with uneven clinical translation	Metabolic, inflammatory, and immunotherapy-related settings	Small heterogeneous trials limit generalization

**Table 3:** Key safety and evidence-limiting considerations relevant to immune-modulating probiotics.

Risk or Limitation	Why It Matters	Highest-Concern Contexts	Practical Implication
Host vulnerability/barrier disruption	Translocation risk and altered immune handling increase	ICU, preterm neonates, severe comorbidity, catheterized or immunocompromised hosts	Require population-specific validation and surveillance
Bloodstream complications may be underdetected	Fungemia and bacteremia are often not prespecified endpoints	Severe illness, central lines, yeast use	Report dedicated, strain-resolved infectious safety outcomes
Immune miscalibration/wrong context	The same pathway can be protective in one host and harmful in another	Pregnancy/infancy, inflammatory endotypes, tissue-specific settings	Match mechanism to host context rather than using class assumptions
Dose/formulation/viability heterogeneity	Exposure level and product composition shape both efficacy and risk	Multi-strain products and poorly standardized formulations	Use transparent strain IDs, dose, matrix, and manufacturing fidelity
Small trials/heterogeneous endpoints	Weakens causal inference and can mask responder groups	Most translational immune studies	Prefer multicenter trials with standardized immune and clinical readouts
Limited strain resolution and mechanistic depth	Species labels obscure divergent biological behavior	Products described only at species level	Prioritize genome-resolved, mechanism-based trial design

## Data Availability

No new data were created or analyzed in this study. Data sharing is not applicable to this article.

## References

[1] Henrick B.M., Rodriguez L., Lakshmikanth T., Pou C., Henckel E., Arzoomand A., Olin A., Wang J., Mikes J., Tan Z. (2021). Bifidobacteria-mediated immune system imprinting early in life. Cell.

[2] Bengsch B. (2022). Günstiger Einfluss von Bifidobakterien auf die Reifung des Immunsystems beim Neugeborenen. Kompass Autoimmun.

[3] Frei R., Akdis M., O’mAhony L. (2015). Prebiotics, probiotics, synbiotics, and the immune system. Curr. Opin. Gastroenterol..

[4] Hao Y. (2025). Gut microbiota-immune axis in the regulation of rheumatoid arthritis: From mechanism to precision probiotic strategies. Mod. Rheumatol..

[5] Jani C.T., Edwards K., Bhanushali C., Zheng X., Salazar A.S., Lopes G., Watson D.C. (2025). Leveraging beneficial microbiome-immune interactions via probiotic use in cancer immunotherapy. Front. Immunol..

[6] Colletti A., Pellizzato M., Cicero A.F. (2023). The Possible Role of Probiotic Supplementation in Inflammation: A Narrative Review. Microorganisms.

[7] Gomes N.D., Sulzbach L.C., Biz G.P., Daleffe A.C.M., Tessing N., Palangana M.L., Vonsowski M., de Lima F.X., Cechini N.R., Costa J.P.R. (2024). Relationship between gut microbiota, probiotics, and obesity in the cellular and molecular mechanisms for the activation of regulatory t cells and control of inflammatory processes: A systematic review. Int. J. Nutrology.

[8] Plaza-Diaz J., Gomez-Llorente C., Fontana L., Gil A. (2014). Modulation of immunity and inflammatory gene expression in the gut, in inflammatory diseases of the gut and in the liver by probiotics. World J. Gastroenterol..

[9] Mohammed Z.S., Hasan A.D. (2025). Immunomodulatory Effects of Bifidobacterium Breve: Mechanism and Therapeutic Potential. Trends Pharm. Biotechnol..

[10] Miková E., Krčmářová E., Černý V., Sklenářová L., Avramová P., Protivová E., Slunéčková B., Akhtar M., Věcek J., Procházka J. (2025). The Ability of Probiotic Strain *Escherichia coli* O83:K24:H31 to Modulate Gut Homeostasis and Immune Function After Antibiotic-Induced Dysbiosis. Probiotics Antimicrob. Proteins.

[11] Dongarrà M.L., Rizzello V., Muccio L., Fries W., Cascio A., Bonaccorsi I., Ferlazzo G. (2012). Mucosal Immunology and Probiotics. Curr. Allergy Asthma Rep..

[12] Vieira A.T., Teixeira M.M., Martins F.S. (2013). The Role of Probiotics and Prebiotics in Inducing Gut Immunity. Front. Immunol..

[13] Pechkurov D.V., Sitkin S.I. (2023). The effect of targeted probiotics on the microbiota–gut–immune system axis. Clin. Pract. Pediatr..

[14] Zimmermann P., Curtis N. (2018). The influence of probiotics on vaccine responses—A systematic review. Vaccine.

[15] Mann E.R., Lam Y.K., Uhlig H.H. (2024). Short-chain fatty acids: Linking diet, the microbiome and immunity. Nat. Rev. Immunol..

[16] Siracusa F., Machicote A., Huber S., Gagliani N. (2025). Diet-derived microbial metabolites as regulators of immune function. Curr. Opin. Immunol..

[17] Mousa W.K., Chehadeh F., Husband S. (2022). Microbial dysbiosis in the gut drives systemic autoimmune diseases. Front. Immunol..

[18] Muneer S.S., Subramani Y. (2019). Dysregulation of gut microbiota composition and function in autoimmune diseases: A meta-analysis. Int. J. Adv. Res. Med..

[19] Cox L.M., Kuchroo V.K. (2024). Autoimmunity and the microbiome. Immunol. Rev..

[20] Quintana F.J., Prinz M. (2017). A gut feeling about multiple sclerosis. Proc. Natl. Acad. Sci. USA.

[21] Geva-Zatorsky N., Sefik E., Kua L., Pasman L., Tan T.G., Ortiz-Lopez A., Yanortsang T.B., Yang L., Jupp R., Mathis D. (2017). Mining the Human Gut Microbiota for Immunomodulatory Organisms. Cell.

[22] Schluter J., Peled J., Taylor B.P., Markey K.A., Smith J.A., Taur Y., Niehus R., Staffas A., Dai A., Fontana E. (2020). The gut microbiota is associated with immune cell dynamics in humans. Nature.

[23] Petersen C., Bell R., Klag K.A., Lee S.H., Soto R., Ghazaryan A., Buhrke K., Atakan Ekiz H., Ost K.S., Boudina S. (2019). T cell–mediated regulation of the microbiota protects against obesity. Science.

[24] Kim C.H. (2023). Complex regulatory effects of gut microbial short-chain fatty acids on immune tolerance and autoimmunity. Cell. Mol. Immunol..

[25] Xie L., Alam J., Marques F.Z., Mackay C.R. (2023). A major mechanism for immunomodulation: Dietary fibres and acid metabolites. Semin. Immunol..

[26] Wang Q., Yi S., Su G., Du Z., Pan S., Huang X., Cao Q., Yuan G., Kijlstra A., Yang P. (2021). Changes in the Gut Microbiome Contribute to the Development of Behcet’s Disease via Adjuvant Effects. Front. Cell Dev. Biol..

[27] Shulzhenko N., Morgun A., Hsiao W., Battle M., Yao M., Gavrilova O., Orandle M., Mayer L., Macpherson A.J., McCoy K.D. (2011). Crosstalk between B lymphocytes, microbiota and the intestinal epithelium governs immunity versus metabolism in the gut. Nat. Med..

[28] Arpaia N. (2014). Keeping peace with the microbiome: Acetate dampens inflammatory cytokine production in intestinal epithelial cells. Immunol. Cell Biol..

[29] Mortha A., Chudnovskiy A., Hashimoto D., Bogunovic M., Spencer S.P., Belkaid Y., Merad M. (2014). Microbiota-Dependent Crosstalk Between Macrophages and ILC3 Promotes Intestinal Homeostasis. Science.

[30] Wang Q., Mackay C.R. (2023). High metabolite concentrations in portal venous blood as a possible mechanism for microbiota effects on the immune system and Western diseases. J. Allergy Clin. Immunol..

[31] Lv J., Hao P., Zhou Y., Liu T., Wang L., Song C., Wang Z., Liu Z., Liu Y. (2025). Role of the intestinal flora-immunity axis in the pathogenesis of rheumatoid arthritis-mechanisms regulating short-chain fatty acids and Th17/Treg homeostasis. Mol. Biol. Rep..

[32] Pang A., Pu S., Pan Y., Huang N., Li D. (2025). Short-chain fatty acids from gut microbiota restore Th17/Treg balance in rheumatoid arthritis: Mechanisms and therapeutic potential. J. Transl. Autoimmun..

[33] Paciolla C., Manganelli M., Di Chiano M., Montenegro F., Gallone A., Sallustio F., Guida G. (2025). Valeric Acid: A Gut-Derived Metabolite as a Potential Epigenetic Modulator of Neuroinflammation in the Gut–Brain Axis. Cells.

[34] Kim M., Qie Y., Park J., Kim C.H. (2016). Gut Microbial Metabolites Fuel Host Antibody Responses. Cell Host Microbe.

[35] Wine E. (2022). Expanding Links Between Gut Microbiota and Bowel Inflammation. Gastroenterology.

[36] Luo W., Li R., Pan C., Luo C. (2025). Gut microbiota–derived metabolites in immunomodulation and gastrointestinal cancer immunotherapy. Front. Immunol..

[37] Ma C., Han M., Heinrich B., Fu Q., Zhang Q., Sandhu M., Agdashian D., Terabe M., Berzofsky J.A., Fako V. (2018). Gut microbiome–mediated bile acid metabolism regulates liver cancer via NKT cells. Science.

[38] Liu H., Xiong X., Zhu W., Wang S., Huang W., Zhu G., Xu H., Yang L. (2025). Gut microbial metabolites in cancer immunomodulation. Mol. Cancer.

[39] Aghamajidi A., Vareki S.M. (2022). The Effect of the Gut Microbiota on Systemic and Anti-Tumor Immunity and Response to Systemic Therapy against Cancer. Cancers.

[40] Zeng M.Y., Cisalpino D., Varadarajan S., Hellman J., Warren H.S., Cascalho M., Inohara N., Núñez G. (2016). Gut Microbiota-Induced Immunoglobulin G Controls Systemic Infection by Symbiotic Bacteria and Pathogens. Immunity.

[41] van Hemert S., Meijerink M., Molenaar D., A Bron P., de Vos P., Kleerebezem M., Wells J.M., Marco M.L. (2010). Identification of Lactobacillus plantarum genes modulating the cytokine response of human peripheral blood mononuclear cells. BMC Microbiol..

[42] Hart A.L., Lammers K., Brigidi P., Vitali B., Rizzello F., Gionchetti P., Campieri M., A Kamm M., Knight S.C., Stagg A.J. (2004). Modulation of human dendritic cell phenotype and function by probiotic bacteria. Gut.

[43] Dong H., Rowland I., Yaqoob P. (2011). Comparative effects of six probiotic strains on immune function *in vitro*. Br. J. Nutr..

[44] Mileti E., Matteoli G., Iliev I.D., Rescigno M. (2009). Comparison of the Immunomodulatory Properties of Three Probiotic Strains of Lactobacilli Using Complex Culture Systems: Prediction for In Vivo Efficacy. PLoS ONE.

[45] Mohamadzadeh M., Pfeiler E.A., Brown J.B., Zadeh M., Gramarossa M., Managlia E., Bere P., Sarraj B., Khan M.W., Pakanati K.C. (2011). Regulation of induced colonic inflammation by *Lactobacillus acidophilus* deficient in lipoteichoic acid. Proc. Natl. Acad. Sci. USA.

[46] Fernandez E.M., Valenti V., Rockel C., Hermann C., Pot B., Boneca I.G., Grangette C. (2011). Anti-inflammatory capacity of selected lactobacilli in experimental colitis is driven by NOD2-mediated recognition of a specific peptidoglycan-derived muropeptide. Gut.

[47] Groeger D., Yao L., Collins F., Larsen I.S., Tan H.-T.T., Healy S., Ambrogi V., Tykwinska K., Schmidt M., Golletz P. (2025). Complementary immunoregulatory effects of Bifidobacterium longum 1714TM associated exopolysaccharide and tryptophan metabolism. Curr. Res. Microb. Sci..

[48] Ghadimi D., Helwig U., Schrezenmeir J., Heller K.J., de Vrese M. (2012). Epigenetic imprinting by commensal probiotics inhibits the IL-23/IL-17 axis in an in vitro model of the intestinal mucosal immune system. J. Leukoc. Biol..

[49] Hayashi A., Sato T., Kamada N., Mikami Y., Matsuoka K., Hisamatsu T., Hibi T., Roers A., Yagita H., Ohteki T. (2013). A Single Strain of Clostridium butyricum Induces Intestinal IL-10-Producing Macrophages to Suppress Acute Experimental Colitis in Mice. Cell Host Microbe.

[50] Sun K.-Y., Xu D.-H., Xie C., Plummer S., Tang J., Yang X.F., Ji X.H. (2017). Lactobacillus paracasei modulates LPS-induced inflammatory cytokine release by monocyte-macrophages via the up-regulation of negative regulators of NF-kappaB signaling in a TLR2-dependent manner. Cytokine.

[51] Lin Y.P., Thibodeaux C.H., Peña J.A., Ferry G.D., Versalovic J. (2008). Probiotic Lactobacillus reuteri suppress proinflammatory cytokines via c-Jun. Inflamm. Bowel Dis..

[52] Dong H., Rowland I., Tuohy K.M., Thomas L.V., Yaqoob P. (2010). Selective effects of *Lactobacillus casei* Shirota on T cell activation, natural killer cell activity and cytokine production. Clin. Exp. Immunol..

[53] Johansson M.A., Björkander S., Mata Forsberg M., Qazi K.R., Celades M.S., Bittmann J., Eberl M., Sverremark-Ekström E. (2016). Probiotic Lactobacilli Modulate Staphylococcus aureus-Induced Activation of Conventional and Unconventional T cells and NK Cells. Front. Immunol..

[54] Christensen H.R., Frøkiær H., Pestka J.J. (2002). Lactobacilli Differentially Modulate Expression of Cytokines and Maturation Surface Markers in Murine Dendritic Cells. J. Immunol..

[55] Kim J.Y., Park M.S., Ji G.E. (2012). Probiotic modulation of dendritic cells co-cultured with intestinal epithelial cells. World J. Gastroenterol..

[56] O’Mahony C., Scully P., O’Mahony D., Murphy S., O’Brien F., Lyons A., Sherlock G., MacSharry J., Kiely B., Shanahan F. (2008). Commensal-Induced Regulatory T Cells Mediate Protection against Pathogen-Stimulated NF-κB Activation. PLoS Pathog..

[57] O’mAhony L., McCarthy J., Kelly P., Hurley G., Luo F., Chen K., O’sUllivan G.C., Kiely B., Collins J.K., Shanahan F. (2005). Lactobacillus and bifidobacterium in irritable bowel syndrome: Symptom responses and relationship to cytokine profiles. Gastroenterology.

[58] Smelt M.J., De Haan B.J., Bron P.A., Van Swam I., Meijerink M., Wells J.M., Faas M.M., De Vos P. (2012). *L. plantarum*, *L. salivarius*, and *L. lactis* Attenuate Th2 Responses and Increase Treg Frequencies in Healthy Mice in a Strain Dependent Manner. PLoS ONE.

[59] Thakur B.K., Saha P., Banik G., Saha D.R., Grover S., Batish V.K., Das S. (2016). Live and heat-killed probiotic Lactobacillus casei Lbs2 protects from experimental colitis through Toll-like receptor 2-dependent induction of T-regulatory response. Int. Immunopharmacol..

[60] Jeon S.G., Kayama H., Ueda Y., Takahashi T., Asahara T., Tsuji H., Tsuji N.M., Kiyono H., Ma J.S., Kusu T. (2012). Probiotic Bifidobacterium breve Induces IL-10-Producing Tr1 Cells in the Colon. PLoS Pathog..

[61] Fujiwara D., Inoue S., Wakabayashi H., Fujii T. (2004). The Anti-Allergic Effects of Lactic Acid Bacteria Are Strain Dependent and Mediated by Effects on both Th1/Th2 Cytokine Expression and Balance. Int. Arch. Allergy Immunol..

[62] Torii A., Torii S., Fujiwara S., Tanaka H., Inagaki N., Nagai H. (2007). Lactobacillus Acidophilus Strain L-92 Regulates the Production of Th1 Cytokine as well as Th2 Cytokines. Allergol. Int..

[63] Meijerink M., Wells J.M., Taverne N., Brouwer M.-L.d.Z., Hilhorst B., Venema K., van Bilsen J. (2012). Immunomodulatory effects of potential probiotics in a mouse peanut sensitization model. FEMS Immunol. Med. Microbiol..

[64] Curciarello R., Canziani K.E., Salto I., Romero E.B., Rocca A., Doldan I., Peton E., Brayer S., Sambuelli A.M., Goncalves S. (2021). Probiotic Lactobacilli Isolated from Kefir Promote Down-Regulation of Inflammatory Lamina Propria T Cells from Patients with Active IBD. Front. Pharmacol..

[65] Kaji R., Kiyoshima-Shibata J., Nagaoka M., Nanno M., Shida K. (2010). Bacterial Teichoic Acids Reverse Predominant IL-12 Production Induced by Certain *Lactobacillus* Strains into Predominant IL-10 Production via TLR2-Dependent ERK Activation in Macrophages. J. Immunol..

[66] Yu R., Zuo F., Ma H., Chen S. (2019). Exopolysaccharide-Producing *Bifidobacterium adolescentis* Strains with Similar Adhesion Property Induce Differential Regulation of Inflammatory Immune Response in Treg/Th17 Axis of DSS-Colitis Mice. Nutrients.

[67] Kil Lee S., Yang K.M., Cheon J.H., Kim T.I., Kim W.H. (2012). Anti-inflammatory Mechanism of *Lactobacillus rhamnosus GG* in Lipopolysaccharide-stimulated HT-29 Cell. Korean J. Gastroenterol..

[68] Haque M., Kaminsky L., Abdulqadir R., Engers J., Kovtunov E., Rawat M., Al-Sadi R., Ma T.Y. (2024). Lactobacillus acidophilus inhibits the TNF-α-induced increase in intestinal epithelial tight junction permeability via a TLR-2 and PI3K-dependent inhibition of NF-κB activation. Front. Immunol..

[69] Mizuno H., Arce L., Tomotsune K., Albarracin L., Funabashi R., Vera D., Islam A., Vizoso-Pinto M.G., Takahashi H., Sasaki Y. (2020). Lipoteichoic Acid Is Involved in the Ability of the Immunobiotic Strain Lactobacillus plantarum CRL1506 to Modulate the Intestinal Antiviral Innate Immunity Triggered by TLR3 Activation. Front. Immunol..

[70] Weiss G., Rasmussen S., Zeuthen L.H., Nielsen B.N., Jarmer H., Jespersen L., Frøkiær H. (2010). *Lactobacillus acidophilus* induces virus immune defence genes in murine dendritic cells by a Toll-like receptor-2-dependent mechanism. Immunology.

[71] Pinto M.G.V., Gómez M.R., Seifert S., Watzl B., Holzapfel W.H., Franz C.M. (2009). Lactobacilli stimulate the innate immune response and modulate the TLR expression of HT29 intestinal epithelial cells in vitro. Int. J. Food Microbiol..

[72] Tomosada Y., Villena J., Murata K., Chiba E., Shimazu T., Aso H., Iwabuchi N., Xiao J.-Z., Saito T., Kitazawa H. (2013). Immunoregulatory Effect of Bifidobacteria Strains in Porcine Intestinal Epithelial Cells Through Modulation of Ubiquitin-Editing Enzyme A20 Expression. PLoS ONE.

[73] Mansilla F., Takagi M., Garcia-Castillo V., Aso H., Nader-Macias M., Vignolo G., Kitazawa H., Villena J. (2020). Modulation of Toll-like receptor-mediated innate immunity in bovine intestinal epithelial cells by lactic acid bacteria isolated from feedlot cattle. Benef. Microbes.

[74] Grabig A., Paclik D., Guzy C., Dankof A., Baumgart D.C., Erckenbrecht J., Raupach B., Sonnenborn U., Eckert J., Schumann R.R. (2006). *Escherichia coli* Strain Nissle 1917 Ameliorates Experimental Colitis via Toll-Like Receptor 2- and Toll-Like Receptor 4-Dependent Pathways. Infect. Immun..

[75] Güttsches A.-K., Löseke S., Zähringer U., Sonnenborn U., Enders C., Gatermann S., Bufe A. (2011). Anti-inflammatory modulation of immune response by probiotic *Escherichia coli* Nissle 1917 in human blood mononuclear cells. J. Endotoxin Res..

[76] Leccese G., Bibi A., Mazza S., Facciotti F., Caprioli F., Landini P., Paroni M. (2020). Probiotic Lactobacillus and Bifidobacterium Strains Counteract Adherent-Invasive *Escherichia coli* (AIEC) Virulence and Hamper IL-23/Th17 Axis in Ulcerative Colitis, but Not in Crohn’s Disease. Cells.

[77] Yang Y., Sui J., Liao W., Wang S., Pan D., Sun G., Gao P., Xiang X., Xia H. (2025). Clinical Evidence on the Health Benefits and Safety of Probiotic Lacticaseibacillus rhamnosus: A Systematic Review. Probiotics Antimicrob. Proteins.

[78] Gao H., Li X., Chen X., Hai D., Wei C., Zhang L., Li P. (2022). The Functional Roles of *Lactobacillus acidophilus* in Different Physiological and Pathological Processes. J. Microbiol. Biotechnol..

[79] Lee A.H., Jimenez D.M.R., Meisel M. (2025). *Limosilactobacillus reuteri*—A probiotic gut commensal with contextual impact on immunity. Gut Microbes.

[80] Qi Y., Huang L., Zeng Y., Li W., Zhou D., Xie J., Xie J., Tu Q., Deng D., Yin J. (2021). Pediococcus pentosaceus: Screening and Application as Probiotics in Food Processing. Front. Microbiol..

[81] Papakonstantinou E., Zacharodimos N., Georgiopoulos G., Athanasaki C., Bothou D.-L., Tsitsou S., Lympaki F., Vitsou-Anastasiou S., Papadopoulou O.S., Delialis D. (2024). Two-Month Consumption of Orange Juice Enriched with Vitamin D3 and Probiotics Decreases Body Weight, Insulin Resistance, Blood Lipids, and Arterial Blood Pressure in High-Cardiometabolic-Risk Patients on a Westernized Type Diet: Results from a Randomized Clinical Trial. Nutrients.

[82] Bengoa A.A., Dardis C., Garrote G.L., Abraham A.G. (2021). Health-Promoting Properties of *Lacticaseibacillus paracasei:* A Focus on Kefir Isolates and Exopolysaccharide-Producing Strains. Foods.

[83] Perez M., Armengol E., Del Casale A., Campedelli I., Aldea-Perona A., Otero M.P., Rodriguez-Palmero M., Espadaler-Mazo J., Huedo P. (2025). *Lactobacillus gasseri* CECT 30648 shows probiotic characteristics and colonizes the vagina of healthy women after oral administration. Microbiol. Spectr..

[84] Zuo J., Huang D., Liu J., Wang Z., Ren Y., Su Y., Ma Y. (2025). Effect of Probiotics Containing *Lactobacillus plantarum* on Blood Lipids: Systematic Review, Meta-Analysis, and Network Pharmacological Analysis. Foods.

[85] Yang Y., Song X., Wang G., Xia Y., Xiong Z., Ai L. (2024). Understanding *Ligilactobacillus salivarius* from Probiotic Properties to Omics Technology: A Review. Foods.

[86] Shirani M., Bagherniya M., Sadeghi O., Tehrani H.G., Eskandari M.H., Sharma M., Askari G. (2025). Effects of supplementation with two probiotic strains (*Lactobacillus helveticus* and *Bifidobacterium longum*) on hormonal status, oxidative stress, and clinical symptoms in women with polycystic ovary syndrome: A randomized clinical trial. Nutr. J..

[87] Ozen M., Piloquet H., Schaubeck M. (2023). *Limosilactobacillus fermentum* CECT5716: Clinical Potential of a Probiotic Strain Isolated from Human Milk. Nutrients.

[88] Kiattiweerasak A., Aumpan N., Chonprasertsuk S., Pornthisarn B., Siramolpiwat S., Bhanthumkomol P., Nunanan P., Issariyakulkarn N., Mahachai V., Yamaoka Y. (2023). Efficacy and safety of *Lacticaseibacillus rhamnosus* R0011 and Lactobacillus helveticus R0052 as an adjuvant for Helicobacter pylori eradication: A double-blind, randomized, placebo-controlled study. Front. Gastroenterol..

[89] Zuhair M.N., Wiriansya E.P., Masyhuri I., Fakhri M., Hatta M., Djaharuddin I., Bukhari A. (2025). Role of *Lacticaseibacillus rhamnosus* GG in the Management of Respiratory Diseases: A Systematic Review and Meta-Analysis. Prev. Nutr. Food Sci..

[90] Hidayat K., Zhang L., Wei H., Zhang W., Qin L., Ou Y., Li N. (2025). The effects of *Lacticaseibacillus rhamnosus* GG supplementation on gastrointestinal and respiratory outcomes: A systematic review and meta-analysis of randomized controlled trials. Food Funct..

[91] Al Kassaa I., Fuad M. (2024). Effects of *Lacticaseibacillus rhamnosus* HN001 on Happiness and Mental Well-Being: Findings from a Randomized Controlled Trial. Nutrients.

[92] Yan R., Wang K., Wang Q., Jiang H., Lu Y., Chen X., Zhang H., Su X., Du Y., Chen L. (2021). Probiotic *Lactobacillus casei* Shirota prevents acute liver injury by reshaping the gut microbiota to alleviate excessive inflammation and metabolic disorders. Microb. Biotechnol..

[93] Lv L., Ren S., Jiang H., Yan R., Chen W., Yan R., Dong J., Shao L., Yu Y. (2024). The oral administration of *Lacticaseibacillus casei* Shirota alleviates acetaminophen-induced liver injury through accelerated acetaminophen metabolism via the liver-gut axis in mice. mSphere.

[94] Li X., Liu Y., Guo X., Ma Y., Zhang H., Liang H. (2021). Effect of Lactobacillus casei on lipid metabolism and intestinal microflora in patients with alcoholic liver injury. Eur. J. Clin. Nutr..

[95] Li X., Liang H. (2022). Effects of Lactobacillus casei on Iron Metabolism and Intestinal Microflora in Rats Exposed to Alcohol and Iron. Turk. J. Gastroenterol..

[96] Mai T.T., Thu P.T., Hang H.T., Trang T.T.T., Yui S., Shigehisa A., Tien V.T., Dung T.V., Nga P.B., Hung N.T. (2020). Efficacy of probiotics on digestive disorders and acute respiratory infections: A controlled clinical trial in young Vietnamese children. Eur. J. Clin. Nutr..

[97] Kikuchi-Hayakawa H., Ishikawa H., Suda K., Gondo Y., Hirasawa G., Nakamura H., Takada M., Kawai M., Matsuda K. (2023). Effects of *Lacticaseibacillus paracasei* Strain Shirota on Daytime Performance in Healthy Office Workers: A Double-Blind, Randomized, Crossover, Placebo-Controlled Trial. Nutrients.

[98] Otaka M., Kikuchi-Hayakawa H., Ogura J., Ishikawa H., Yomogida Y., Ota M., Hidese S., Ishida I., Aida M., Matsuda K. (2021). Effect of *Lacticaseibacillus paracasei* Strain Shirota on Improvement in Depressive Symptoms, and Its Association with Abundance of Actinobacteria in Gut Microbiota. Microorganisms.

[99] Ahn S.-I., Cho S., Choi N.-J. (2020). Effect of dietary probiotics on colon length in an inflammatory bowel disease–induced murine model: A meta-analysis. J. Dairy Sci..

[100] Andreozzi V., Cuoco S., Balestrieri M., Fierro F., Ferrara N., Erro R., Di Filippo M., Barbella G., Memoli M.C., Silvestri A. (2024). Synbiotic supplementation may globally improve non-motor symptoms in patients with stable Parkinson’s disease: Results from an open label single-arm study. Sci. Rep..

[101] Ou S.J.L., Yusri H., Yang D., Khoo C.M., Liu M.H. (2024). Effects of Moderate Consumption of a Probiotic-Fermented Sour Beer on the Inflammatory, Immunity, Lipid Profile, and Gut Microbiome of Healthy Men in a Participant-Blinded, Randomized-Controlled Within-Subject Crossover Study. Food Sci. Nutr..

[102] Patterson E., Griffin S.M., Ibarra A., Ellsiepen E., Hellhammer J. (2020). *Lacticaseibacillus paracasei* Lpc-37^®^ improves psychological and physiological markers of stress and anxiety in healthy adults: A randomized, double-blind, placebo-controlled and parallel clinical trial (the Sisu study). Neurobiol. Stress.

[103] Mäkelä S.M., Griffin S.M., Reimari J., Evans K.C., Hibberd A.A., Yeung N., Ibarra A., Junnila J., Turunen J., Beboso R. (2023). Efficacy and safety of *Lacticaseibacillus paracasei* Lpc-37^®^ in students facing examination stress: A randomized, triple-blind, placebo-controlled clinical trial (the ChillEx study). Brain Behav. Immun. Health.

[104] Roos S., Dahlgren A., Mao Y., Pallin A., Stanisz A.M., Forsythe P., Kunze W., Hellström P.M. (2025). Therapeutic Value of *Lactobacillus gasseri* 345A in Chronic Constipation. Neurogastroenterol. Motil..

[105] Qi F., Fan S., Fang C., Ge L., Lyu J., Huang Z., Zhao S., Zou Y., Huang L., Liu X. (2023). Orally administrated Lactobacillus gasseri TM13 and *Lactobacillus crispatus* LG55 can restore the vaginal health of patients recovering from bacterial vaginosis. Front. Immunol..

[106] Ohtsu T., Haruma K., Ide Y., Takagi A. (2021). The Effect of Continuous Intake of *Lactobacillus gasseri* OLL2716 on Mild to Moderate Delayed Gastric Emptying: A Randomized Controlled Study. Nutrients.

[107] Sawada D., Sugawara T., Hirota T., Nakamura Y. (2022). Effects of *Lactobacillus gasseri* CP2305 on Mild Menopausal Symptoms in Middle-Aged Women. Nutrients.

[108] Mändar R., Sõerunurk G., Štšepetova J., Smidt I., Rööp T., Kõljalg S., Saare M., Ausmees K., Le D., Jaagura M. (2023). Impact of *Lactobacillus crispatus*-containing oral and vaginal probiotics on vaginal health: A randomised double-blind placebo controlled clinical trial. Benef. Microbes.

[109] He X., Chen W., Zhou X., Hu G., Wei J., Liu Y., Cai L., Zhang Z., Chen T. (2024). The Therapeutic Potential of Lactobacillus crispatus for Chronic Endometritis: A Comprehensive Clinical Trial and Experimental Investigation. Probiotics Antimicrob. Proteins.

[110] Hemmerling A., Mitchell C.M., Demby S., Ghebremichael M., Elsherbini J., Xu J., Xulu N., Shih J., Dong K., Govender V. (2025). Effect of the vaginal live biotherapeutic LACTIN-V (*Lactobacillus crispatus* CTV-05) on vaginal microbiota and genital tract inflammation among women at high risk of HIV acquisition in South Africa: A phase 2, randomised, placebo-controlled trial. Lancet Microbe.

[111] Di Pierro F., Sampugnaro E.G., Lomeo G.E., Guarneri M.F., Cusenza S., Pivetti A., Khan A., Rabbani F., Memon N.M., Cazzaniga M. (2025). Effect of orally administered *L. crispatus* M247 in favoring HR-HPV clearance and CST shift: Results from a randomized, multi-center, placebo-controlled trial. Sci. Rep..

[112] Hong Q., Wang J., Zhang H., Liu X., Liu Z. (2023). Study of the effect of *Lactobacillus crispatus* FSCDJY67L3 on Helicobacter Pylori eradication: A double-blind randomized controlled clinical trial. Front. Immunol..

[113] Watanabe I., Suzuki N., Takara T. (2024). Supplementation with heat-sterilized *Lactobacillus crispatus* strain KT-11 stimulates the T cell–related immune function of healthy Japanese adults: A pilot randomized, placebo-controlled, double-blind, parallel-group study. Nutr. Res..

[114] Bi Z., Wang Q., Yang T., Liu Y., Yuan J., Li L., Guo Y. (2023). Effect of *Lactobacillus delbrueckii* subsp. *lactis* on vaginal radiotherapy for gynecological cancer. Sci. Rep..

[115] Olorocisimo J.P., Diaz L.A., Co D.E., Carag H.M., Ibana J.A., Velarde M.C. (2022). *Lactobacillus delbrueckii* reduces anxiety-like behavior in zebrafish through a gut microbiome—Brain crosstalk. Neuropharmacology.

[116] Baek H.-I., Kwon S.-Y., Noh H.-J., Son S.Y., Joo J.C., Park S.J. (2025). Efficacy and Safety of *Lactobacillus delbrueckii* subsp. *lactis* CKDB001 Supplementation on Cognitive Function in Mild Cognitive Impairment: A Randomized, Double-Blind, Placebo-Controlled Clinical Trial. Nutrients.

[117] Quaresma L.S., Santos R.C.V., Gomes G.C., Américo M.F., Campos G.M., Laguna J.G., Barroso F.A.L., Azevedo V., de Jesus L.C.L. (2024). Multidrug resistance profile in *Lactobacillus delbrueckii*: A food industry species with probiotic properties. World J. Microbiol. Biotechnol..

[118] Bui L.T.K., Alam Bushra F., Rattananon P., Rimi A.A., Lee C., Tahmid S.M., Tisha S.A., Jisan I.F., Das R., Sneha J.I. (2025). Strategic antagonism: How Lactobacillus plantarum counters Staphylococcus aureus pathogenicity. Front. Microbiol..

[119] Chen W.-L., Deng F.-S., Tsai Y.-C. (2025). *Lactiplantibacillus plantarum* as a Psychobiotic Strategy Targeting Parkinson’s Disease: A Review and Mechanistic Insights. Nutrients.

[120] Sabatini G., Boccadoro I., Prete R., Battista N., Corsetti A. (2025). Autism Spectrum Disorder: From Experimental Models to Probiotic Application with a Special Focus on *Lactiplantibacillus plantarum*. Nutrients.

[121] Saeed M., Al-Khalaifah H., Al-Nasser A., Al-Surrayai T. (2025). Feeding the future: A new potential nutritional impact of *Lactiplantibacillus plantarum* and its promising interventions in future for poultry industry. Poult. Sci..

[122] Krivec J.L., Bratina P., Valcl A., Manfreda K.L., Petrovčič A., Benedik E., Obermajer T., Matijašić B.B., ŠeTina U., Rupnik M. (2024). Effects of *Limosilactobacillus reuteri* DSM 17938 in neonates exposed to antibiotics: A randomised controlled trial. Benef. Microbes.

[123] Dinleyici E.C., Ozen M., Guven S., Dalgic N., Karbuz A., Sutcu M., Turel O., Oz F.N., Kirli U., Durmus S.Y. (2025). Effect of *Limosilactobacillus reuteri* DSM17938 to prevent antibiotic-associated diarrhea in children: Prospective, multi-center, randomized, placebo-controlled clinical trial (PEARL Study). Eur. J. Pediatr..

[124] Wen X., Wang J., Sun J., Jin Z., Li Q., Wang G., Zhao J., Wang Z., Tian P. (2025). *Limosilactobacillus reuteri* CCFM1388 Enhances Exercise Endurance by Modulating Intestinal Bile Acid Metabolism and Cholesterol Absorption. Mol. Nutr. Food Res..

[125] Ivashkin V., Maev I., Poluektova E., Sinitsa A., Avalueva E., Mnatsakanyan M., Simanenkov V., Karpeeva J., Kopylova D., Kuprina I. (2024). Efficacy and Safety of Postbiotic Contained Inactivated *Lactobacillus reuteri* (*Limosilactobacillus reuteri*) DSM17648 as Adjuvant Therapy in the Eradication of Helicobacter pylori in Adults with Functional Dyspepsia: A Randomized Double-Blind Placebo-Controlled Trial. Clin. Transl. Gastroenterol..

[126] Kim Y.K., Kim H.J., Yun M.-K., Lee S., Kang D.-J. (2025). Effects of Paraprobiotic *Limosilactobacillus fermentum* HDB1098 on Hangover Improvement in Humans: A Randomized Double-Blind Placebo-Controlled Crossover Clinical Trial. J. Microbiol. Biotechnol..

[127] Zhao Y., Niu X., Zhang Y., Zhao L., Zhang L., He J., Zhang Q., Mao Y., Wang F., Zhao X. (2025). Impact of supplementing *Limosilactobacillus fermentum* MN–LF23 on the eradication of Helicobacter pylori with 14–day standard quadruple therapy: A randomized, double–blind, placebo–controlled trial. Nutr. J..

[128] Bae W., Jung W., Shin S.L., Kim T., Sohn M., Suk J., Jung I., Lee Y.I., Lee J.H. (2024). Heat-treated *Limosilactobacillus fermentum* LM1020 with menthol, salicylic acid, and panthenol promotes hair growth and regulates hair scalp microbiome balance in androgenetic alopecia: A double-blind, randomized and placebo-controlled clinical trial. J. Cosmet. Dermatol..

[129] Lacerda D.C., da Costa P.C.T., Pontes P.B., dos Santos L.A.C., Neto J.P.R.C., Luis C.C.S., Brito V.P.d.S., Alves J.L.d.B. (2022). Potential role of *Limosilactobacillus fermentum* as a probiotic with anti-diabetic properties: A review. World J. Diabetes.

[130] Nascimento L.C.P.D., Lacerda D.C., Ferreira D.J.S., de Souza E.L., Alves J.L.d.B. (2022). *Limosilactobacillus fermentum*, Current Evidence on the Antioxidant Properties and Opportunities to be Exploited as a Probiotic Microorganism. Probiotics Antimicrob. Proteins.

[131] Satomi S., Waki N., Arakawa C., Fujisawa K., Suzuki S., Suganuma H. (2021). Effects of Heat-Killed *Levilactobacillus brevis* KB290 in Combination with β-Carotene on Influenza Virus Infection in Healthy Adults: A Randomized Controlled Trial. Nutrients.

[132] Wang Q., Zhang W., Liu J., Qin W., Cai J. (2025). Exopolysaccharide of *Levilactobacillus brevis* M-10 Improved Physiological and Biochemical Indicators and Gut Microbiota in DSS-Induced Colitis Mice. Curr. Microbiol..

[133] Altamura S., Lombardi F., Augello F.R., Barone A., Giannoni M., Cinque B., Pietropaoli D. (2025). *Levilactobacillus brevis* CD2 as a multifaceted probiotic to preserve oral health: Results of a double-blind, randomized, placebo-controlled trial in healthy adults. J. Transl. Med..

[134] Sanchez M.G., Passot S., Campoy S., Olivares M., Fonseca F. (2021). *Ligilactobacillus salivarius* functionalities, applications, and manufacturing challenges. Appl. Microbiol. Biotechnol..

[135] López C.H., Martín M.J.S., Moreta A.H., Urrutia M.C., García I.C., Morillo C.D., Blanco-Rojo R., Sáez M.E., Olivares M., Arroyo R. (2025). *Ligilactobacillus salivarius* CECT5713 Increases Term Pregnancies in Women with Infertility of Unknown Origin: A Randomized, Triple-Blind, Placebo-Controlled Trial. Nutrients.

[136] Ding L., Wang Y., Jiang Z., Tang X., Mao B., Zhao J., Chen W., Zhang Q., Cui S. (2024). Effects of *Lactiplantibacillus plantarum* CCFM1214 and *Ligilactobacillus salivarius* CCFM1215 on halitosis: A double-blind, randomized controlled trial. Food Funct..

[137] GálVez A., de Terán E.D., Espinosa J., Pérez-Pedregosa J., Bartha-Rasero J., del Valle J., Cuerva M., JiménEz E., Badiola C. (2024). *Ligilactobacillus salivarius* V4II-90 eradicates Group B Streptococcus colonisation during pregnancy: A randomised, double-blind, placebo-controlled trial. Benef. Microbes.

[138] Wiącek J., Skonieczna-Żydecka K., Łoniewski I., Styburski D., Kaczmarczyk M., Karolkiewicz J. (2025). A Randomized Controlled Trial Evaluating the Effects of a Probiotic Containing Lactobacillus helveticus R0052 and Bifidobacterium longum R0175 on Gastrointestinal Symptoms and Metabolomic Profiles in Female Dancers. Int. J. Mol. Sci..

[139] Wada H., Mawatari T., Saito Y., Azuma N., Iwama Y. (2024). *Lactobacillus helveticus* Induces Two Types of Dendritic Cell Activation and Effectively Suppresses Onset of the Common Cold: A Randomized, Double-Blind, Placebo-Controlled Trial. Nutrients.

[140] Tanihiro R., Yuki M., Sakano K., Sasai M., Sawada D., Ebihara S., Hirota T. (2024). Effects of Heat-Treated *Lactobacillus helveticus* CP790-Fermented Milk on Gastrointestinal Health in Healthy Adults: A Randomized Double-Blind Placebo-Controlled Trial. Nutrients.

[141] Mutoh N., Kakiuchi I., Hiraku A., Iwabuchi N., Kiyosawa K., Igarashi K., Tanaka M., Nakamura M., Miyasaka M. (2023). Heat-killed *Lactobacillus helveticus* improves mood states: A randomised, double-blind, placebo-controlled study. Benef. Microbes.

[142] Carlman H.M.T.E., Rode J., König J., Repsilber D., Hutchinson A.N., Thunberg P., Persson J., Kiselev A., Pruessner J.C., Brummer R.J. (2022). Probiotic Mixture Containing *Lactobacillus helveticus*, *Bifidobacterium longum* and *Lactiplantibacillus plantarum* Affects Brain Responses to an Arithmetic Stress Task in Healthy Subjects: A Randomised Clinical Trial and Proof-of-Concept Study. Nutrients.

[143] Jiang S., Cai L., Lv L., Li L. (2021). Pediococcus pentosaceus, a future additive or probiotic candidate. Microb. Cell Factories.

[144] Takeshita K., Hishiki H., Takei H., Ikari N., Tanaka S., Iijima Y., Ogata H., Fujishiro K., Tominaga T., Konno Y. (2024). The Effectiveness of Heat-Killed *Pediococcus acidilactici* K15 in Preventing Respiratory Tract Infections in Preterm Infants: A Pilot Double-Blind, Randomized, Placebo-Controlled Study. Nutrients.

[145] Li D.-X., Hu Q.-M., Xu C.-C., Yang H.-Y., Liu J.-K., Sun Y.-F., Wang G., Wang J., Zhou Z.-H. (2025). Efficacy of *Pediococcus acidilactici* CCFM6432 in alleviating anhedonia in major depressive disorder: A randomized controlled trial. World J. Psychiatry.

[146] Feng P., Yang J., Zhao S., Ling Z., Han R., Wu Y., Salama E.-S., Kakade A., Khan A., Jin W. (2022). Human supplementation with *Pediococcus acidilactici* GR-1 decreases heavy metals levels through modifying the gut microbiota and metabolome. npj Biofilms Microbiomes.

[147] Hagen P.C., Skelley J.W. (2019). Efficacy of Bifidobacterium Species in Prevention of Necrotizing Enterocolitis in Very-Low Birth Weight Infants. A Systematic Review. J. Pediatr. Pharmacol. Ther..

[148] Holscher H.D., Czerkies L.A., Cekola P., Litov R., Benbow M., Santema S., Alexander D.D., Perez V., Sun S., Saavedra J.M. (2012). *Bifidobacterium lactis* Bb12 Enhances Intestinal Antibody Response in Formula-Fed Infants. J. Parenter. Enter. Nutr..

[149] Quigley E., Groeger D., O’mahony L., Bourke J., Scully P., Dinan T., Shanahan F., Kiely B. (2011). Oral Administration of the Probiotic Bifidobacterium Infantis 35624 to Humans Induces Immunoregulatory Responses In Vivo. Am. J. Gastroenterol..

[150] Saturio S., Nogacka A.M., Alvarado-Jasso G.M., Salazar N., Reyes-Gavilán C.G.d.L., Gueimonde M., Arboleya S. (2021). Role of Bifidobacteria on Infant Health. Microorganisms.

[151] Ku S., Park M.S., Ji G.E., You H.J. (2016). Review on *Bifidobacterium bifidum* BGN4: Functionality and Nutraceutical Applications as a Probiotic Microorganism. Int. J. Mol. Sci..

[152] Nocerino R., De Filippis F., Cecere G., Marino A., Micillo M., Di Scala C., De Caro C., Calignano A., Bruno C., Paparo L. (2020). The therapeutic efficacy of *Bifidobacterium animalis* subsp. *lactis* BB-12^®^ in infant colic: A randomised, double blind, placebo-controlled trial. Aliment. Pharmacol. Ther..

[153] Uusitupa H.-M., Rasinkangas P., Lehtinen M.J., Mäkelä S.M., Airaksinen K., Anglenius H., Ouwehand A.C., Maukonen J. (2020). Bifidobacterium animalis subsp. lactis 420 for Metabolic Health: Review of the Research. Nutrients.

[154] Lau A.-Y., Yanagisawa N., Hor Y.-Y., Lew L.-C., Ong J.-S., Chuah L.-O., Lee Y.-Y., Choi S.-B., Rashid F., Wahid N. (2018). Bifidobacterium longum BB536 alleviated upper respiratory illnesses and modulated gut microbiota profiles in Malaysian pre-school children. Benef. Microbes.

[155] Pinto-Sanchez M.I., Hall G.B., Ghajar K., Nardelli A., Bolino C., Lau J.T., Martin F.-P., Cominetti O., Welsh C., Rieder A. (2017). Probiotic *Bifidobacterium longum* NCC3001 Reduces Depression Scores and Alters Brain Activity: A Pilot Study in Patients with Irritable Bowel Syndrome. Gastroenterology.

[156] Bocchio F., Mancabelli L., Milani C., Lugli G.A., Tarracchini C., Longhi G., De Conto F., Turroni F., Ventura M. (2024). Compendium of *Bifidobacterium*-based probiotics: Characteristics and therapeutic impact on human diseases. Microbiome Res. Rep..

[157] Plaza-Diaz J., Ruiz-Ojeda F.J., Gil-Campos M., Gil A. (2019). Mechanisms of Action of Probiotics. Adv. Nutr..

[158] McFarland L.V., Evans C.T., Goldstein E.J.C. (2018). Strain-Specificity and Disease-Specificity of Probiotic Efficacy: A Systematic Review and Meta-Analysis. Front. Med..

[159] Collins F.W.J., Vera-Jiménez N.I., Wellejus A. (2025). Understanding the probiotic health benefits of Bifidobacterium animalis subsp. lactis, BB-12™. Front. Microbiol..

[160] Dimidi E., Zdanaviciene A., Christodoulides S., Taheri S., Louis P., Duncan P.I., Emami N., Crabbé R., De Castro C.A., McLean P. (2018). Randomised clinical trial: *Bifidobacterium lactis* NCC2818 probiotic vs placebo, and impact on gut transit time, symptoms, and gut microbiology in chronic constipation. Aliment. Pharmacol. Ther..

[161] Jungersen M., Wind A., Johansen E., Christensen J.E., Stuer-Lauridsen B., Eskesen D. (2014). The Science behind the Probiotic Strain *Bifidobacterium animalis* subsp. *lactis* BB-12^®^. Microorganisms.

[162] Tremblay A., Bronner S., Binda S. (2023). Review and Perspectives on *Bifidobacterium lactis* for Infants’ and Children’s Health. Microorganisms.

[163] Ouwehand A.C., Bergsma N., Parhiala R., Lahtinen S., Gueimonde M., Finne-Soveri H., Strandberg T., Pitkã¤Lã¤ K., Salminen S. (2008). *Bifidobacterium* microbiota and parameters of immune function in elderly subjects. FEMS Immunol. Med. Microbiol..

[164] Wu B.-B., Yang Y., Xu X., Wang W.-P. (2015). Effects of Bifidobacterium supplementation on intestinal microbiota composition and the immune response in healthy infants. World J. Pediatr..

[165] Li Y., Arai S., Kato K., Iwabuchi S., Iwabuchi N., Muto N., Motobayashi H., Ebihara S., Tanaka M., Hashimoto S. (2023). The Potential Immunomodulatory Effect of *Bifidobacterium longum* subsp. *longum* BB536 on Healthy Adults through Plasmacytoid Dendritic Cell Activation in the Peripheral Blood. Nutrients.

[166] Akatsu H., Iwabuchi N., Xiao J., Matsuyama Z., Kurihara R., Okuda K., Yamamoto T., Maruyama M. (2012). Clinical Effects of Probiotic *Bifidobacterium longum* BB536 on Immune Function and Intestinal Microbiota in Elderly Patients Receiving Enteral Tube Feeding. J. Parenter. Enter. Nutr..

[167] Tojo R., Suárez A., Clemente M.G., de los Reyes-Gavilán C.G., Margolles A., Gueimonde M., Ruas-Madiedo P. (2014). Intestinal microbiota in health and disease: Role of bifidobacteria in gut homeostasis. World J. Gastroenterol..

[168] Baratte-Euloge P. (1992). Action Comparée Sur La Flore Intestinale De Trois Laits Fermentés Au Bifidobacterium: Évaluation De Propriétés Probiotiques et Du Comportement De La Souche BB 536 De *Bifidobacterium longum* Chez L’homme. Ph.D. Thesis.

[169] Allen A.P., Clarke G., Cryan J.F., Quigley E.M.M., Dinan T.G. (2017). *Bifidobacterium infantis 35624* and other probiotics in the management of irritable bowel syndrome. Strain specificity, symptoms, and mechanisms. Curr. Med. Res. Opin..

[170] Chen J., Chen X., Ho C.L. (2021). Recent Development of Probiotic Bifidobacteria for Treating Human Diseases. Front. Bioeng. Biotechnol..

[171] Marras L., Caputo M., Bisicchia S., Soato M., Bertolino G., Vaccaro S., Inturri R. (2021). The Role of Bifidobacteria in Predictive and Preventive Medicine: A Focus on Eczema and Hypercholesterolemia. Microorganisms.

[172] Groeger D., O’mAhony L., Murphy E.F., Bourke J.F., Dinan T.G., Kiely B., Shanahan F., Quigley E.M. (2013). *Bifidobacterium infantis* 35624 modulates host inflammatory processes beyond the gut. Gut Microbes.

[173] McFarland L.V. (2020). Efficacy of Single-Strain Probiotics Versus Multi-Strain Mixtures: Systematic Review of Strain and Disease Specificity. Digestive Diseases and Sciences.

[174] Krumbeck J.A., Rasmussen H.E., Hutkins R.W., Clarke J., Shawron K., Keshavarzian A., Walter J. (2018). Probiotic Bifidobacterium strains and galactooligosaccharides improve intestinal barrier function in obese adults but show no synergism when used together as synbiotics. Microbiome.

[175] Cukrowska B., Bierła J.B., Zakrzewska M., Klukowski M., Maciorkowska E. (2020). The Relationship between the Infant Gut Microbiota and Allergy. The Role of *Bifidobacterium breve* and Prebiotic Oligosaccharides in the Activation of Anti-Allergic Mechanisms in Early Life. Nutrients.

[176] Sun S., Luo L., Liang W., Yin Q., Guo J., Rush A.M., Lv Z., Liang Q., Fischbach M.A., Sonnenburg J.L. (2020). *Bifidobacterium* alters the gut microbiota and modulates the functional metabolism of T regulatory cells in the context of immune checkpoint blockade. Proc. Natl. Acad. Sci. USA.

[177] Pysariev A. (2025). The application of bifidobacteria strains in the practice of pediatricians and general practitioners of family medicine (literature review). Mod. Pediatr. Ukr..

[178] Gargari G., Taverniti V., Balzaretti S., Ferrario C., Gardana C., Simonetti P., Guglielmetti S. (2016). Consumption of a Bifidobacterium bifidum Strain for 4 Weeks Modulates Dominant Intestinal Bacterial Taxa and Fecal Butyrate in Healthy Adults. Appl. Environ. Microbiol..

[179] Al-Sadi R., Dharmaprakash V., Nighot P., Guo S., Nighot M., Do T., Ma T.Y. (2021). Bifidobacterium bifidum Enhances the Intestinal Epithelial Tight Junction Barrier and Protects against Intestinal Inflammation by Targeting the Toll-like Receptor-2 Pathway in an NF-κB-Independent Manner. Int. J. Mol. Sci..

[180] Schiffrin E.J., Rochat F., Link-Amster H., Aeschlimann J.M., Donnet-Hughes A. (1995). Immunomodulation of Human Blood Cells Following the Ingestion of Lactic Acid Bacteria. J. Dairy Sci..

[181] Amin A., Patel D., Shah N., Phatak A., Nimbalkar S. (2015). Report on Kangaroo Care Practices in a Tertiary Level NICU in Western India—Scope for Improvement. Pediatrics.

[182] A Sjælland M., Philipsen M.T., Henriksen T.B., Skipper J., Rubak S. (2025). Probiotics in Term Infants: Clinical Impact of Infant-Type Bifidobacteria: A Systematic Review and Meta-analyses. J. Nutr..

[183] Turroni F., Duranti S., Milani C., Lugli G.A., van Sinderen D., Ventura M. (2019). *Bifidobacterium bifidum*: A Key Member of the Early Human Gut Microbiota. Microorganisms.

[184] Dinleyici E.C., Kara A., Ozen M., Vandenplas Y. (2014). *Saccharomyces boulardii* CNCM I-745 in different clinical conditions. Expert Opin. Biol. Ther..

[185] Gopalan S., Ganapathy S., Mitra M., Joshi D.K., Veligandla K.C., Rathod R., Kotak B.P. (2023). Neha Unique Properties of Yeast Probiotic Saccharomyces boulardii CNCM I-745: A Narrative Review. Cureus.

[186] Han S.Y., Kim G.H. (2016). Clinical Manifestations of Laryngopharyngeal Reflux. J. Neurogastroenterol. Motil..

[187] McFarland L.V. (2010). Systematic review and meta-analysis of *Saccharomyces boulardii* in adult patients. World J. Gastroenterol..

[188] McFarland L.V., Li T. (2024). *Saccharomyces boulardii* CNCM I-745 for Prevention of Antibiotic-Associated Diarrhea and Clostridioides difficile in China: Systematic Review and Meta-Analysis. J. Dig. Dis. Hepatol..

[189] Szajewska H., Skórka A. (2009). *Saccharomyces boulardii* for treating acute gastroenteritis in children: Updated meta-analysis of randomized controlled trials. Aliment. Pharmacol. Ther..

[190] Altcheh J., Carosella M.V., Ceballos A., D’aNdrea U., Jofre S.M., Marotta C., Mugeri D., Sabbaj L., Soto A., Josse C.M. (2022). Randomized, direct comparison study of *Saccharomyces boulardii* CNCM I-745 versus multi-strained Bacillus clausii probiotics for the treatment of pediatric acute gastroenteritis. Medicine.

[191] Xu L., Wang Y., Wang Y., Fu J., Sun M., Mao Z., Vandenplas Y. (2016). A double-blinded randomized trial on growth and feeding tolerance with *Saccharomyces boulardii* CNCM I-745 in formula-fed preterm infants. J. Pediatr..

[192] Moré M.I., Swidsinski A. (2015). Saccharomyces boulardii CNCM I-745 supports regeneration of the intestinal microbiota after diarrheic dysbiosis—A review. Clin. Exp. Gastroenterol..

[193] Kelly C.P., Nguyen C.C., Palmieri L.J., Pallav K., Dowd S.E., Humbert L., Seksik P., Bado A., Coffin B., Rainteau D. (2019). Saccharomyces boulardii CNCM I-745 Modulates the Fecal Bile Acids Metabolism During Antimicrobial Therapy in Healthy Volunteers. Front. Microbiol..

[194] O’bRien E.S., Robert A., Gauthier D., Le Cavorzin A., Planchais J., Roux X., Verleye M., Castagné V. (2023). Protective effects of Saccharomyces boulardii CNCM I-745 in an experimental model of NSAID-induced enteropathy. Benef. Microbes.

[195] I Moré M., Vandenplas Y. (2018). *Saccharomyces boulardii* CNCM I-745 Improves Intestinal Enzyme Function: A Trophic Effects Review. Clin. Med. Insights Gastroenterol..

[196] Gao H., Li Y., Sun J., Xu H., Wang M., Zuo X., Fu Q., Guo Y., Chen Z., Zhang P. (2021). *Saccharomyces boulardii* Ameliorates Dextran Sulfate Sodium-Induced Ulcerative Colitis in Mice by Regulating NF-*κ*B and Nrf2 Signaling Pathways. Oxidative Med. Cell. Longev..

[197] Everard A., Matamoros S., Geurts L., Delzenne N.M., Cani P.D. (2014). Saccharomyces boulardii Administration Changes Gut Microbiota and Reduces Hepatic Steatosis, Low—Grade Inflammation, and Fat Mass in Obese and Type 2 Diabetic db/db Mice. mBio.

[198] Waitzberg D., Guarner F., Hojsak I., Ianiro G., Polk D.B., Sokol H. (2024). Can the Evidence-Based Use of Probiotics (Notably Saccharomyces boulardii CNCM I-745 and Lactobacillus rhamnosus GG) Mitigate the Clinical Effects of Antibiotic-Associated Dysbiosis?. Adv. Ther..

[199] Vinayagamoorthy K., Pentapati K.C., Prakash H. (2023). Epidemiology of *Saccharomyces* fungemia: A systematic review. Med. Mycol..

[200] Bourreille A., Cadiot G., Le Dreau G., Laharie D., Beaugerie L., Dupas J.-L., Marteau P., Rampal P., Moyse D., Saleh A. (2013). Saccharomyces boulardii Does Not Prevent Relapse of Crohn’s Disease. Clin. Gastroenterol. Hepatol..

[201] Mourey F., Decherf A., Jeanne J.-F., Clément-Ziza M., Grisoni M.-L., Machuron F., Legrain-Raspaud S., Bourreille A., Desreumaux P. (2022). *Saccharomyces cerevisiae* I-3856 in irritable bowel syndrome with predominant constipation. World J. Gastroenterol..

[202] Cayzeele-Decherf A., Pélerin F., Leuillet S., Douillard B., Housez B., Cazaubiel M., Jacobson G.K., Jüsten P., Desreumaux P. (2017). *Saccharomyces cerevisiae* CNCM I-3856 in irritable bowel syndrome: An individual subject meta-analysis. World J. Gastroenterol..

[203] Spiller R., Pélerin F., Decherf A.C., Maudet C., Housez B., Cazaubiel M., Jüsten P. (2015). Randomized double blind placebo-controlled trial of *Saccharomyces cerevisiae* CNCM I-3856 in irritable bowel syndrome: Improvement in abdominal pain and bloating in those with predominant constipation. United Eur. Gastroenterol. J..

[204] Gayathri R., Aruna T., Malar S., Shilpa B., Dhanasekar K.R. (2019). Efficacy of Saccharomyces cerevisiae CNCM I-3856 as an add-on therapy for irritable bowel syndrome. Int. J. Color. Dis..

[205] Roussel C., Sivignon A., de Vallée A., Garrait G., Denis S., Tsilia V., Ballet N., Vandekerckove P., Van de Wiele T., Barnich N. (2018). Anti-infectious properties of the probiotic Saccharomyces cerevisiae CNCM I-3856 on enterotoxigenic *E. coli* (ETEC) strain H10407. Appl. Microbiol. Biotechnol..

[206] Decherf A., Dehay E., Boyer M., Clément-Ziza M., Rodriguez B., Legrain-Raspaud S. (2020). Recovery of *Saccharomyces cerevisiae* CNCM I-3856 in Vaginal Samples of Healthy Women after Oral Administration. Nutrients.

[207] Mourey F., Sureja V.M., Kheni D.M., Shah P., Parikh D., Upadhyay U., Satia M., Shah D.M., Troise C., Decherf A. (2020). A Multicenter, Randomized, Double-blind, Placebo-controlled Trial of *Saccharomyces boulardii* in Infants and Children with Acute Diarrhea. Pediatr. Infect. Dis. J..

[208] Karbownik M.S., Kręczyńska J., Kwarta P., Cybula M., Wiktorowska-Owczarek A., Kowalczyk E., Pietras T., Szemraj J. (2020). Effect of Supplementation with *Saccharomyces Boulardii* on Academic Examination Performance and Related Stress in Healthy Medical Students: A Randomized, Double-Blind, Placebo-Controlled Trial. Nutrients.

[209] Karbownik M.S., Kręczyńska J., Wiktorowska-Owczarek A., Kwarta P., Cybula M., Stilinović N., Pietras T., Kowalczyk E. (2022). Decrease in Salivary Serotonin in Response to Probiotic Supplementation with *Saccharomyces boulardii* in Healthy Volunteers Under Psychological Stress: Secondary Analysis of a Randomized, Double-Blind, Placebo-Controlled Trial. Front. Endocrinol..

[210] Ganesh B., Richter J., Blaut M., Loh G. (2012). Enterococcus faecium NCIMB 10415 does not protect interleukin-10 knock-out mice from chronic gut inflammation. Benef. Microbes.

[211] Sudha M.R. (2011). Safety assessment studies of probiotic Saccharomyces boulardii strain Unique 28 in Sprague-Dawley rats. Benef. Microbes.

[212] Wang M., Gao C., Lessing D.J., Chu W. (2024). Saccharomyces cerevisiae SC-2201 Attenuates AOM/DSS-Induced Colorectal Cancer by Modulating the Gut Microbiome and Blocking Proinflammatory Mediators. Probiotics Antimicrob. Proteins.

[213] Hyink O., Wescombe P.A., Upton M., Ragland N., Burton J.P., Tagg J.R. (2007). Salivaricin A2 and the Novel Lantibiotic Salivaricin B Are Encoded at Adjacent Loci on a 190-Kilobase Transmissible Megaplasmid in the Oral Probiotic Strain *Streptococcus salivarius* K12. Appl. Environ. Microbiol..

[214] Do H., Li Z.-R., Tripathi P.K., Mitra S., Guerra S., Dash A., Weerasekera D., Makthal N., Shams S., Aggarwal S. (2024). Engineered probiotic overcomes pathogen defences using signal interference and antibiotic production to treat infection in mice. Nat. Microbiol..

[215] Cosseau C., Devine D.A., Dullaghan E., Gardy J.L., Chikatamarla A., Gellatly S., Yu L.L., Pistolic J., Falsafi R., Tagg J. (2008). The Commensal *Streptococcus salivarius* K12 Downregulates the Innate Immune Responses of Human Epithelial Cells and Promotes Host-Microbe Homeostasis. Infect. Immun..

[216] MacDonald K.W., Chanyi R.M., Macklaim J.M., Cadieux P.A., Reid G., Burton J.P. (2021). *Streptococcus salivarius* inhibits immune activation by periodontal disease pathogens. BMC Oral Heath.

[217] Laws G.L., Hale J.D.F., Kemp R.A. (2021). Human Systemic Immune Response to Ingestion of the Oral Probiotic *Streptococcus salivarius* BLIS K12. Probiotics Antimicrob. Proteins.

[218] Laws G.A., Harold L.K., Tagg J.R., Hale J.D.F. (2022). Interferon Gamma Response in Human Saliva Following Exposure to the Oral Probiotic *Streptococcus salivarius* BLIS K12. Probiotics Antimicrob. Proteins.

[219] Bertuccioli A., Gervasi M., Annibalini G., Binato B., Perroni F., Rocchi M.B.L., Sisti D., Amatori S. (2023). Use of *Streptococcus salivarius* K12 in supporting the mucosal immune function of active young subjects: A randomised double-blind study. Front. Immunol..

[220] Di Pierro F., Colombo M., Zanvit A., Risso P., Rottoli A.S. (2014). Use of *Streptococcus salivarius* K12 in the prevention of streptococcal and viral pharyngotonsillitis in children. Drug Health Patient Saf..

[221] Di Pierro F., Colombo M., Giuliani M.G., Danza M.L., Basile I., Bollani T., Conti A.M., Zanvit A., Rottoli A.S. (2016). Effect of administration of *Streptococcus salivarius* K12 on the occurrence of streptococcal pharyngo-tonsillitis, scarlet fever and acute otitis media in 3 years old children. Eur. Rev. Med. Pharmacol. Sci..

[222] Di Pierro F., Risso P., Poggi E., Timitilli A., Bolloli S., Bruno M., Caneva E., Campus R., Giannattasio A. (2018). Use of *Streptococcus salivarius* K12 to reduce the incidence of pharyngo-tonsillitis and acute otitis media in children: A retrospective analysis in not-recurrent pediatric subjects. Minerva Pediatr..

[223] Doyle H., Pierse N., Tiatia R., Williamson D., Baker M., Crane J. (2018). Effect of Oral Probiotic *Streptococcus salivarius* K12 on Group A Streptococcus Pharyngitis: A Pragmatic Trial in Schools. Pediatr. Infect. Dis. J..

[224] Wilcox C., Stuart B., Leaver H., Lown M., Willcox M., Moore M., Little P. (2019). Effectiveness of the probiotic *Streptococcus salivarius* K12 for the treatment and/or prevention of sore throat: A systematic review. Clin. Microbiol. Infect..

[225] Peng X., Li Z., Pei Y., Zheng S., Liu J., Wang J., Li R., Xu X. (2024). *Streptococcus salivarius* K12 Alleviates Oral Mucositis in Patients Undergoing Radiotherapy for Malignant Head and Neck Tumors: A Randomized Controlled Trial. J. Clin. Oncol..

[226] Hu L., Mao Q., Zhou P., Lv X., Hua H., Yan Z. (2019). Effects of *Streptococcus salivarius* K12 with nystatin on oral candidiasis—RCT. Oral Dis..

[227] Passariello C., Di Nardo F., Polimeni A., Di Nardo D., Testarelli L. (2020). Probiotic *Streptococcus salivarius* Reduces Symptoms of Denture Stomatitis and Oral Colonization by *Candida albicans*. Appl. Sci..

[228] Horz H., Meinelt A., Houben B., Conrads G. (2007). Distribution and persistence of probiotic *Streptococcus salivarius* K12 in the human oral cavity as determined by real-time quantitative polymerase chain reaction. Oral Microbiol. Immunol..

[229] Cernioglo K., Kalanetra K.M., Meier A., Lewis Z.T., Underwood M.A., Mills D.A., Smilowitz J.T. (2021). Multi-Strain Probiotic Supplementation with a Product Containing Human-Native *S. salivarius* K12 in Healthy Adults Increases Oral *S. salivarius*. Nutrients.

[230] Burton J.P., Chilcott C.N., Wescombe P.A., Tagg J.R. (2010). Extended Safety Data for the Oral Cavity Probiotic *Streptococcus salivarius* K12. Probiotics Antimicrob. Proteins.

[231] Burton J.P., Wescombe P.A., Moore C.J., Chilcott C.N., Tagg J.R. (2006). Safety Assessment of the Oral Cavity Probiotic *Streptococcus salivarius* K12. Appl. Environ. Microbiol..

[232] Burton J.P., Drummond B.K., Chilcott C.N., Tagg J.R., Thomson W.M., Hale J.D.F., Wescombe P.A. (2013). Influence of the probiotic *Streptococcus salivarius* strain M18 on indices of dental health in children: A randomized double-blind, placebo-controlled trial. J. Med. Microbiol..

[233] Kumar P., Choudhary A., Patnana A.K., Pathak K., Shekh T.M., Gosai P. (2021). Comparative Analysis of Shear Bond Strength of Composites to the Sodium Ascorbate Hydrogel-treated Bleached Enamel Surfaces: An In Vitro Analysis. Int. J. Clin. Pediatr. Dent..

[234] Jansen P.M., Abdelbary M.M.H., Conrads G. (2021). A concerted probiotic activity to inhibit periodontitis-associated bacteria. PLoS ONE.

[235] Mallikarjun S.B., Salim H.P., Raju S., Surendranath A.R. (2023). Randomized Clinical Trial of Oral Probiotic *Streptococcus salivarius* M18 on Salivary Streptococcus mutans in Preprimary Children. Int. J. Clin. Pediatr. Dent..

[236] Chen W.-J., Sharma L.A., Shao P., Griffith T., Love R., Jain R., Hale J., Sharma A. (2025). Adjunctive use of *Streptococcus salivarius* M18 probiotic in the treatment of periodontitis: A randomized controlled trial. 3 Biotech.

[237] Burton J.P., Wescombe P.A., Macklaim J.M., Chai M.H.C., MacDonald K., Hale J.D.F., Tagg J., Reid G., Gloor G.B., Cadieux P.A. (2013). Persistence of the Oral Probiotic *Streptococcus salivarius* M18 Is Dose Dependent and Megaplasmid Transfer Can Augment Their Bacteriocin Production and Adhesion Characteristics. PLoS ONE.

[238] Kolling G.L., Wu M., Warren C.A., Durmaz E., Klaenhammer T.R., Guerrant R.L. (2012). Lactic acid production by Streptococcus thermophilus alters Clostridium difficile infection and in vitro Toxin A production. Gut Microbes.

[239] Khalil R. (2009). Evidence for probiotic potential of a capsular-producing Streptococcus thermophilus CHCC 3534 strain. Pol. J. Microbiol..

[240] Nam E.H., Lee M., Kim H., Kim D., Lee Y., Jung Y.H., Yang J., Shin M. (2025). The impact of Streptococcus thermophilus IDCC 2201 on gut microbiota and its potential as a prophylactic agent for colorectal cancer. Sci. Rep..

[241] Li Q., Hu W., Liu W.-X., Zhao L.-Y., Huang D., Liu X.-D., Chan H., Zhang Y., Zeng J.-D., Coker O.O. (2021). Streptococcus thermophilus Inhibits Colorectal Tumorigenesis Through Secreting β-Galactosidase. Gastroenterology.

[242] Fernandez N., Wrzosek L., Radziwill-Bienkowska J.M., Ringot-Destrez B., Duviau M.-P., Noordine M.-L., Laroute V., Robert V., Cherbuy C., Daveran-Mingot M.-L. (2018). Characterization of Mucus-Related Properties of Streptococcus thermophilus: From Adhesion to Induction. Front. Physiol..

[243] Dargahi N., Johnson J.C., Apostolopoulos V. (2021). Immune Modulatory Effects of Probiotic *Streptococcus thermophilus* on Human Monocytes. Biologics.

[244] Kang X., Liang H., Luo Y., Li Z., He F., Han X., Zhang L. (2021). Streptococcus thermophilus MN-ZLW-002 Can Inhibit Pre-adipocyte Differentiation through Macrophage Activation. Biol. Pharm. Bull..

[245] Martinović A., Chittaro M., Mora D., Arioli S. (2023). The Ability of *Streptococcus thermophilus* BT01 to Modulate Urease Activity in Healthy Subjects’ Fecal Samples Depends on the Biomass Production Process. Mol. Nutr. Food Res..

[246] EL Hadad S., Alsolami M., Aldahlawi A., Alrahimi J., Basingab F., Hassoubah S., Alothaid H. (2021). In Vivo evidence: Repression of mucosal immune responses in mice with colon cancer following sustained administration of Streptococcus thermophiles. Saudi J. Biol. Sci..

[247] Kapse N., Pisu V., Dhakephalkar T., Margale P., Shetty D., Wagh S., Dagar S., Dhakephalkar P.K. (2024). Unveiling the Probiotic Potential of *Streptococcus thermophilus* MCC0200: Insights from In Vitro Studies Corroborated with Genome Analysis. Microorganisms.

[248] Kruis W., Frič P., Pokrotnieks J., Lukáš M., Fixa B., Kaščák M., A Kamm M., Weismueller J., Beglinger C., Stolte M. (2004). Maintaining remission of ulcerative colitis with the probiotic *Escherichia coli* Nissle 1917 is as effective as with standard mesalazine. Gut.

[249] Losurdo G., Iannone A., Contaldo A., Ierardi E., Di Leo A., Principi M. (2015). *Escherichia coli* Nissle 1917 in Ulcerative Colitis Treatment: Systematic Review and Meta-analysis. J. Gastrointest. Liver Dis..

[250] Zyrek A.A., Cichon C., Helms S., Enders C., Sonnenborn U., Schmidt M.A. (2006). Molecular mechanisms underlying the probiotic effects of *Escherichia coli* Nissle 1917 involve ZO-2 and PKC? redistribution resulting in tight junction and epithelial barrier repair. Cell. Microbiol..

[251] Ukena S.N., Singh A., Dringenberg U., Engelhardt R., Seidler U., Hansen W., Bleich A., Bruder D., Franzke A., Rogler G. (2007). Probiotic *Escherichia coli* Nissle 1917 Inhibits Leaky Gut by Enhancing Mucosal Integrity. PLoS ONE.

[252] Sturm A., Rilling K., Baumgart D.C., Gargas K., Abou-Ghazalé T., Raupach B., Eckert J., Schumann R.R., Enders C., Sonnenborn U. (2005). *Escherichia coli* Nissle 1917 Distinctively Modulates T-Cell Cycling and Expansion via Toll-Like Receptor 2 Signaling. Infect. Immun..

[253] Wehkamp J., Harder J., Wehkamp K., Meissner B.W.-V., Schlee M., Enders C., Sonnenborn U., Nuding S., Bengmark S., Fellermann K. (2004). NF-κB- and AP-1-Mediated Induction of Human Beta Defensin-2 in Intestinal Epithelial Cells by *Escherichia coli* Nissle 1917: A Novel Effect of a Probiotic Bacterium. Infect. Immun..

[254] Ma Y., Fu W., Hong B., Wang X., Jiang S., Wang J. (2023). Antibacterial MccM as the Major Microcin in *Escherichia coli* Nissle 1917 Against Pathogenic Enterobacteria. Int. J. Mol. Sci..

[255] Altenhoefer A., Oswald S., Sonnenborn U., Enders C., Schulze J., Hacker J., A Oelschlaeger T. (2004). The probiotic *Escherichia coli* strain Nissle 1917 interferes with invasion of human intestinal epithelial cells by different enteroinvasive bacterial pathogens. FEMS Immunol. Med. Microbiol..

[256] Kamada N., Maeda K., Inoue N., Hisamatsu T., Okamoto S., Hong K.S., Yamada T., Watanabe N., Tsuchimoto K., Ogata H. (2008). Nonpathogenic *Escherichia coli* Strain Nissle 1917 Inhibits Signal Transduction in Intestinal Epithelial Cells. Infect. Immun..

[257] Wang Y., Deng H., Xiao L., Pan Y. (2024). *Escherichia coli* Nissle 1917 Protects against Sepsis-Induced Intestinal Damage by Regulating the SCFA/GPRs Signaling Pathway. Microorganisms.

[258] Secher T., Kassem S., Benamar M., Bernard I., Boury M., Barreau F., Oswald E., Saoudi A. (2017). Oral Administration of the Probiotic Strain *Escherichia coli* Nissle 1917 Reduces Susceptibility to Neuroinflammation and Repairs Experimental Autoimmune Encephalomyelitis-Induced Intestinal Barrier Dysfunction. Front. Immunol..

[259] Olier M., Marcq I., Salvador-Cartier C., Secher T., Dobrindt U., Boury M., Bacquié V., Penary M., Gaultier E., Nougayrède J.-P. (2012). Genotoxicity of *Escherichia coli* Nissle 1917 strain cannot be dissociated from its probiotic activity. Gut Microbes.

[260] Massip C., Branchu P., Bossuet-Greif N., Chagneau C.V., Gaillard D., Martin P., Boury M., Sécher T., Dubois D., Nougayrède J.-P. (2019). Deciphering the interplay between the genotoxic and probiotic activities of *Escherichia coli* Nissle 1917. PLoS Pathog..

[261] Sha S., Xu B., Kong X., Wei N., Liu J., Wu K. (2014). Preventive effects of *Escherichia coli* strain Nissle 1917 with different courses and different doses on intestinal inflammation in murine model of colitis. Inflamm. Res..

[262] Gronbach K., Eberle U., MülLer M., ÖlSchlägEr T.A., Dobrindt U., LeithäuSer F., Niess J.H., DörIng G., Reimann J., Autenrieth I.B. (2010). Safety of Probiotic *Escherichia coli* Strain Nissle 1917 Depends on Intestinal Microbiota and Adaptive Immunity of the Host. Infect. Immun..

[263] Depommier C., Everard A., Druart C., Plovier H., Van Hul M., Vieira-Silva S., Falony G., Raes J., Maiter D., Delzenne N.M. (2019). Supplementation with *Akkermansia muciniphila* in overweight and obese human volunteers: A proof-of-concept exploratory study. Nat. Med..

[264] Liu Q., Lu W., Tian F., Zhao J., Zhang H., Hong K., Yu L. (2021). *Akkermansia muciniphila* Exerts Strain-Specific Effects on DSS-Induced Ulcerative Colitis in Mice. Front. Cell. Infect. Microbiol..

[265] Bian X., Wu W., Yang L., Lv L., Wang Q., Li Y., Ye J., Fang D., Wu J., Jiang X. (2019). Administration of *Akkermansia muciniphila* Ameliorates Dextran Sulfate Sodium-Induced Ulcerative Colitis in Mice. Front. Microbiol..

[266] Qu S., Fan L., Qi Y., Xu C., Hu Y., Chen S., Liu W., Liu W., Si J. (2021). *Akkermansia muciniphila* Alleviates Dextran Sulfate Sodium (DSS)-Induced Acute Colitis by NLRP3 Activation. Microbiol. Spectr..

[267] Liu H., Huang R., Shen B., Huang C., Zhou Q., Xu J., Chen S., Lin X., Wang J., Zhao X. (2024). Live *Akkermansia muciniphila* boosts dendritic cell retinoic acid synthesis to modulate IL-22 activity and mitigate colitis in mice. Microbiome.

[268] Soheilipour M., Noursina A., Nekookhoo M., Malekpour E., Mirzaei S.A., Shafieyoun M.H., Rouzbahani H., Chitsazi M.R., Safari F. (2025). The pathobiont role of *Akkermansia muciniphila* in colorectal cancer: A systematic review. BMC Gastroenterol..

[269] Yasueda A., Mizushima T., Nezu R., Sumi R., Tanaka M., Nishimura J., Kai Y., Hirota M., Osawa H., Nakajima K. (2015). The effect of *Clostridium butyricum* MIYAIRI on the prevention of pouchitis and alteration of the microbiota profile in patients with ulcerative colitis. Surg. Today.

[270] Hagihara M., Yamashita R., Matsumoto A., Mori T., Kuroki Y., Kudo H., Oka K., Takahashi M., Nonogaki T., Yamagishi Y. (2018). The impact of *Clostridium butyricum* MIYAIRI 588 on the murine gut microbiome and colonic tissue. Anaerobe.

[271] Hagihara M., Kuroki Y., Ariyoshi T., Higashi S., Fukuda K., Yamashita R., Matsumoto A., Mori T., Mimura K., Yamaguchi N. (2020). *Clostridium butyricum* Modulates the Microbiome to Protect Intestinal Barrier Function in Mice with Antibiotic-Induced Dysbiosis. iScience.

[272] Hagihara M., Yamashita R., Matsumoto A., Mori T., Inagaki T., Nonogaki T., Kuroki Y., Higashi S., Oka K., Takahashi M. (2019). The impact of probiotic *Clostridium butyricum* MIYAIRI 588 on murine gut metabolic alterations. J. Infect. Chemother..

[273] Seo M., Inoue I., Tanaka M., Matsuda N., Nakano T., Awata T., Katayama S., Alpers D.H., Komoda T. (2013). *Clostridium butyricum* MIYAIRI 588 Improves High-Fat Diet-Induced Non-Alcoholic Fatty Liver Disease in Rats. Dig. Dis. Sci..

[274] Maia L.P., Levi Y.L.d.A.S., Prado R.L.D., Santinoni C.d.S., Marsicano J.A. (2019). Effects of probiotic therapy on serum inflammatory markers: A systematic review and meta-analysis. J. Funct. Foods.

[275] Goldenberg J.Z., Lytvyn L., Steurich J., Parkin P., Mahant S., Johnston B.C. (2015). Probiotics for the prevention of pediatric antibiotic-associated diarrhea. Cochrane Database Syst. Rev..

[276] Newberry S.J., Hempel S., Maher A.R., Wang Z., Miles J.N.V., Shanman R., Johnsen B., Shekelle P.G. (2012). Probiotics for the Prevention and Treatment of Antibiotic-Associated Diarrhea. JAMA.

[277] Storr M., Stengel A. (2021). Klinische Evidenz zu Probiotika in der Prävention einer Antibiotika-assoziierten Diarrhö. MMW Fortschritte Med..

[278] McFarland L.V. (2006). Meta-Analysis of Probiotics for the Prevention of Antibiotic Associated Diarrhea and the Treatment of Clostridium difficile Disease. Am. J. Gastroenterol..

[279] Sandradevi I.D.A.M.P. (2025). A Rigorous Systematic Review of Probiotics for the Prevention of Antibiotic-Associated Diarrhea: Efficacy, Safety, Strain Specificity, and Risk Stratification in Adult and Populations. Int. J. Med. Sci. Health Res..

[280] McFarland L.V. (2015). Probiotics for the Primary and Secondary Prevention of *C. difficile* Infections: A Meta-analysis and Systematic Review. Antibiotics.

[281] Hao Q., Lu Z., Dong B.R., Huang C.Q., Wu T. (2015). Probiotics for preventing acute upper respiratory tract infections. Cochrane Database Syst. Rev..

[282] Chen K., Ma W., Zhong J., Yang P., He N., Li X., Zhai Y., Yuan J., Liong M.-T., Liu C. (2026). Probiotic Supplementation Reduces RRTIs and Enhances Gut Microbial and Immunity in Children: A Randomized Controlled Trial. J. Microbiol. Biotechnol..

[283] Marushko Y., Hyshchak T., Todyka Y. (2021). Prophylactic Effect of a Probiotic Intervention in Children Prone to Acute Upper Respiratory Tract Infections: A Randomized Controlled Trial. Med. Sci. Ukr..

[284] Strauss M., Mičetić-Turk D., Pogačar M.Š., Fijan S. (2021). Probiotics for the Prevention of Acute Respiratory-Tract Infections in Older People: Systematic Review. Healthcare.

[285] Nelwan E.J., Herdiman A., Kalaij A.G.I., Lauditta R.K., Yusuf S.M., Suarthana E. (2024). Role of probiotic as adjuvant in treating various infections: A systematic review and meta-analysis. BMC Infect. Dis..

[286] Grin P.M., Kowalewska P.M., Alhazzani W., Fox-Robichaud A.E. (2013). Lactobacillus for preventing recurrent urinary tract infections in women: Meta-analysis. Can J Urol..

[287] Lou L., Liang T., Lv M. (2026). Probiotics in the ICU: A scoping review of evidence for infection prevention. J. Transl. Med..

[288] Zhao M., Shen C., Ma L. (2018). Treatment efficacy of probiotics on atopic dermatitis, zooming in on infants: A systematic review and meta-analysis. Int. J. Dermatol..

[289] Husein-ElAhmed H., Steinhoff M. (2023). Effects of probiotic supplementation in adult with atopic dermatitis: A systematic review with meta-analysis. Clin. Exp. Dermatol..

[290] Agamy A.M., Chen H.-H. (2025). Effects of Probiotic Supplementation in Adults with Atopic Dermatitis: A Meta-Analysis. J. Dermatol. Res..

[291] Fijan S., Kolč N., Hrašovec M., Jamtvedt G., Pogačar M.Š., Turk D.M., Maver U. (2023). Single-Strain Probiotic Lactobacilli for the Treatment of Atopic Dermatitis in Children: A Systematic Review and Meta-Analysis. Pharmaceutics.

[292] Kim K., Lee E., Kim M., Lee K.S., Sol I.S., Min T.K., Yang H.-J., Hong S.-J. (2025). Therapeutic effectiveness of probiotics for atopic dermatitis: A systematic review and meta-analysis of randomized controlled trials with subgroup analysis. Asian Pac. J. Allergy Immunol..

[293] Pelucchi C., Chatenoud L., Turati F., Galeone C., Moja L., Bach J.-F., La Vecchia C. (2012). Probiotics Supplementation During Pregnancy or Infancy for the Prevention of Atopic Dermatitis. Epidemiology.

[294] Kalliomäki M., Salminen S., Arvilommi H., Kero P., Koskinen P., Isolauri E. (2001). Probiotics in primary prevention of atopic disease: A randomised placebo-controlled trial. Lancet.

[295] Tang L.-J., Chen J., Shen Y. (2012). Meta-analysis of probiotics preventing allergic diseases in infants. Chin. J. Pediatr..

[296] Mansfield J.A., Bergin S.W., Cooper J.R., Olsen C.H. (2014). Comparative Probiotic Strain Efficacy in the Prevention of Eczema in Infants and Children: A Systematic Review and Meta-Analysis. Mil. Med..

[297] Du X., Wang L., Wu S., Yuan L., Tang S., Xiang Y., Qu X., Liu H., Qin X., Liu C. (2019). Efficacy of probiotic supplementary therapy for asthma, allergic rhinitis, and wheeze: A meta-analysis of randomized controlled trials. Allergy Asthma Proc..

[298] Elazab N., Mendy A., Gasana J., Vieira E.R., Quizon A., Forno E. (2013). Probiotic Administration in Early Life, Atopy, and Asthma: A Meta-analysis of Clinical Trials. Pediatrics.

[299] Güvenç I.A., Muluk N.B., Mutlu F.Ş., Eşki E., Altıntoprak N., Oktemer T., Cingi C. (2016). Do Probiotics have a role in the Treatment of Allergic Rhinitis? A Comprehensive Systematic Review and Metaanalysis. Am. J. Rhinol. Allergy.

[300] Lu C., Gao Y., Dong S., Sun Y., Sun M., Han X., Li B., Li C., Zhang Y., Li M. (2025). Efficacy of different probiotic regimens for allergic rhinitis: A network meta-analysis. Complement. Ther. Clin. Pract..

[301] Estevinho M.M., Yuan Y., Rodríguez-Lago I., Sousa-Pimenta M., Dias C.C., Acosta M.B., Jairath V., Magro F. (2024). Efficacy and safety of probiotics in IBD: An overview of systematic reviews and updated meta-analysis of randomized controlled trials. United Eur. Gastroenterol. J..

[302] Shen J., Zuo Z.-X., Mao A.-P. (2014). Effect of Probiotics on Inducing Remission and Maintaining Therapy in Ulcerative Colitis, Crohn’s Disease, and Pouchitis. Inflamm. Bowel Dis..

[303] Estevinho M.M., Rodriguez-Lago I., Dias C.C., Acosta M.B.-D., Magro F. (2024). P793 Efficacy and safety of probiotics in inflammatory bowel disease: An umbrella review and updated meta-analysis of randomized controlled trials. J. Crohn’s Colitis.

[304] Jonkers D., Penders J., Masclee A., Pierik M. (2012). Probiotics in the Management of Inflammatory Bowel Disease. Drugs.

[305] Chen M., Feng Y., Liu W. (2021). Efficacy and safety of probiotics in the induction and maintenance of inflammatory bowel disease remission: A systematic review and meta-analysis. Ann. Palliat. Med..

[306] Mozafarybazargany M., Khonsari M., Sokoty L., Ejtahed H.-S., Qorbani M. (2023). The effects of probiotics on gastrointestinal symptoms and microbiota in patients with celiac disease: A systematic review and meta-analysis on clinical trials. Clin. Exp. Med..

[307] Poto R., Laniro G., de Paulis A., Spadaro G., Marone G., Gasbarrini A., Varricchi G. (2023). Is there a role for microbiome-based approach in common variable immunodeficiency?. Clin. Exp. Med..

[308] Tunc H.A., E Childs C., Swann J.R., Calder P.C. (2024). The effect of oral probiotics on response to vaccination in older adults: A systematic review of randomised controlled trials. Age Ageing.

[309] Blázquez-Bondia C., Parera M., Català-Moll F., Casadellà M., Elizalde-Torrent A., Aguiló M., Espadaler-Mazo J., Santos J.R., Paredes R., Noguera-Julian M. (2022). Probiotic effects on immunity and microbiome in HIV-1 discordant patients. Front. Immunol..

[310] Akker C.H.v.D., van Goudoever J.B., Szajewska H., Embleton N.D., Hojsak I., Reid D., Shamir R. (2018). Probiotics for Preterm Infants. J. Pediatr. Gastroenterol. Nutr..

[311] Ye W.-Y., Cai Y. (2025). Akkermansia muciniphila: A microbial guardian against oxidative stress–gut microbiota crosstalk and clinical prospects. J. Transl. Med..

[312] Jian H., Liu Y., Wang X., Dong X., Zou X. (2023). *Akkermansia muciniphila* as a Next-Generation Probiotic in Modulating Human Metabolic Homeostasis and Disease Progression: A Role Mediated by Gut–Liver–Brain Axes?. Int. J. Mol. Sci..

[313] Cheng D., Xie M. (2020). A review of a potential and promising probiotic candidate—*Akkermansia muciniphila*. J. Appl. Microbiol..

[314] Ni Z., Ye D. (2026). The impact of gut microbiota modulation on responses to immune checkpoint inhibitors in cancer. Acta Microbiol. Immunol. Hung..

[315] Effendi R.M.R.A., Anshory M., Kalim H., Dwiyana R.F., Suwarsa O., Pardo L.M., Nijsten T.E.C., Thio H.B. (2022). *Akkermansia muciniphila* and *Faecalibacterium prausnitzii* in Immune-Related Diseases. Microorganisms.

[316] Han E.J., Ahn J.-S., Chae Y.J., Chung H.-J. (2025). Immunomodulatory Roles of *Faecalibacterium prausnitzii* and *Akkermansia muciniphila* in Autoimmune Diseases: Mechanistic Insights and Therapeutic Potential. Clin. Rev. Allergy Immunol..

[317] Segers A., de Vos W.M. (2023). Mode of action of *Akkermansia muciniphila* in the intestinal dialogue: Role of extracellular proteins, metabolites and cell envelope components. Microbiome Res. Rep..

[318] Shaheen N., Khursheed W., Gurung B., Wang S. (2025). *Akkermansia muciniphila*: A key player in gut microbiota-based disease modulation. Microbiol. Res..

[319] Li L., Li M., Chen Y., Yu Z., Cheng P., Yu Z., Cheng W., Zhang W., Wang Z., Gao X. (2024). Function and therapeutic prospects of next-generation probiotic *Akkermansia muciniphila* in infectious diseases. Front. Microbiol..

[320] Cani P.D., Depommier C., Derrien M., Everard A., de Vos W.M. (2022). *Akkermansia muciniphila*: Paradigm for next-generation beneficial microorganisms. Nat. Rev. Gastroenterol. Hepatol..

[321] Bae M., Cassilly C.D., Liu X., Park S.-M., Tusi B.K., Chen X., Kwon J., Filipčík P., Bolze A.S., Liu Z. (2022). *Akkermansia muciniphila* phospholipid induces homeostatic immune responses. Nature.

[322] Wu X., Yu D., Ma Y., Fang X., Sun P. (2025). Function and therapeutic potential of Amuc_1100, an outer membrane protein of Akkermansia muciniphila: A review. Int. J. Biol. Macromol..

[323] Ostrowski B., Krawczyk B. (2025). *Akkermansia Muciniphila* is Associated with Human Health: What Should we Know?. Adv. Microbiol..

[324] Rolhion N., Sokol H. (2025). Targeting the gut microbiome in inflammatory bowel disease: From concept to clinical reality. Intest. Res..

[325] Loo K.-Y., Thong J.Y.H., Tan L.T.-H., Letchumanan V., Chan K.-G., Lee L.-H., Law J.W.-F. (2024). A Current Overview of Next-Generation Probiotics and Their Prospects in Health and Disease Management. Prog. Microbes Mol. Biol..

[326] E Hudson L., McDermott C.D., Stewart T.P., Hudson W.H., Rios D., Fasken M.B., Corbett A.H., Lamb T.J. (2016). Characterization of the Probiotic Yeast *Saccharomyces boulardii* in the Healthy Mucosal Immune System. PLoS ONE.

[327] Rebeck O.N., Wallace M.J., Prusa J., Ning J., Evbuomwan E.M., Rengarajan S., Habimana-Griffin L., Kwak S., Zahrah D., Tung J. (2024). A yeast-based oral therapeutic delivers immune checkpoint inhibitors to reduce intestinal tumor burden. Cell Chem. Biol..

[328] Sulaiman N.N.Y., Noor N.A.M., Aazmi M.S., Ismail M.I., Jasni A.H., Wu Y.S., Alabsi A.M., Lim S.M., Ramasamy K., Ismail M.F. (2025). Unveiling Postbiotics: Advancing Definitions, Mechanisms and Their Impact on Health and Other Functional Applications. Trends Sci..

[329] Farahani H.E., Pourhajibagher M., Asgharzadeh S., Bahador A. (2025). Postbiotics: Novel Modulators of Gut Health, Metabolism, and Their Mechanisms of Action. Probiotics Antimicrob. Proteins.

[330] Amobonye A., Pillay B., Hlope F., Asong S.T., Pillai S. (2025). Postbiotics: An insightful review of the latest category in functional biotics. World J. Microbiol. Biotechnol..

[331] Wang Y.-F., Li Z.-R., Gopinath D., Wang Q., Liu Y.-F., Luo P., Su X.-S. (2025). Short-chain fatty acids: Microbial metabolites bridging tumor immunity and cancer therapy through microenvironment modulation. Cancer Adv..

[332] Delgado S., Sánchez B., Margolles A., Ruas-Madiedo P., Ruiz L. (2020). Molecules Produced by Probiotics and Intestinal Microorganisms with Immunomodulatory Activity. Nutrients.

[333] Gao Y., Liu Y., Ma T., Liang Q., Sun J., Wu X., Song Y., Nie H., Huang J., Mu G. (2025). Fermented Dairy Products as Precision Modulators of Gut Microbiota and Host Health: Mechanistic Insights, Clinical Evidence, and Future Directions. Foods.

[334] Hamid K.H., Mahamat A.A. (2025). Socio-Economic Study of Duck Farming in the Peri-Urban Area of N’Djamena. J. Anim. Sci. Livest. Prod..

[335] Orberg E.T., Meedt E., Hiergeist A., Xue J., Heinrich P., Ghimire S., Miltiadous O., Lindner S., Schwarz A., Janssen K.-P. (2023). Bacterial and Bacteriophage Consortia Are Associated with Protective Intestinal Immuno-Modulatory Metabolites in Allogeneic Stem Cell Transplantation Patients. Blood.

[336] Pavithra N., Devi M., Nirenjen S., Keerthana B., Kumar V.K.G., Yogalakshmi R., Priyadharshni M.G., Harikrishnan N. (2025). Exploring the microbial basis of postbiotic and paraprobiotic therapy in ulcerative colitis. Arch. Microbiol..

[337] Yan R., Zeng X., Shen J., Wu Z., Guo Y., Du Q., Tu M., Pan D. (2024). New clues for postbiotics to improve host health: A review from the perspective of function and mechanisms. J. Sci. Food Agric..

[338] Perl M., Fante M.A., Herfeld K., Scherer J.N., Poeck H., Orberg E.T. (2025). Microbiota-derived metabolites: Key modulators of cancer immunotherapies. Med.

[339] Montazeri-Najafabady N. (2025). From One-Size-Fits-All to Precision Medicine: The Promise of Personalized Probiotics. Probiotics Antimicrob. Proteins.

[340] Manan M.A. (2025). The role of probiotics in personalized therapeutics: Advances in gut microbe-driven interventions. Microbe.

[341] Tiwari G., Narayanasamy A.B., Jothinathan M.K.D., Gurumurthy H., Kareemulla S., Prasad P.D., Mohan V.K. (2025). The Future of Biotics: Individualized Probiotic, Prebiotic, and Postbiotic Solutions. Curr. Pharmacogenom. Pers. Med..

[342] Mehmood M.S., Masood M., Danaf N. (2025). Engineered food-borne probiotics delivering checkpoint-inhibitor modulators. Ann. Med. Surg..

[343] Seyyedi Z., Kashani H.H., Parchebafi A., Ghayoumi R., Lari M.M.H., Hosseini E.S. (2025). Integrating probiotics and microbiome-derived metabolites into cancer therapy: Mechanistic insights, multi-omics strategies, and clinical potential. Curr. Res. Biotechnol..

[344] Redenti A., Im J., Redenti B., Li F., Rouanne M., Sheng Z., Sun W., Gurbatri C.R., Huang S., Komaranchath M. (2024). Probiotic neoantigen delivery vectors for precision cancer immunotherapy. Nature.

[345] Li N., Yin L., Wang J., Zhang J., Tong Y. (2025). Programmable probiotics as next-generation living therapeutics: Bridging synthetic biology and precision medicine. Curr. Opin. Biotechnol..

[346] Cecil D.L., Rodmaker E., Strenk S., Corulli L., Fredricks D.N., Disis M.L. (2023). Abstract 6434: A precision probiotic therapeutic promotes a more efficacious type I immune response and limits breast cancer growth in mice. Cancer Res..

[347] Park E.-J., Lee Y.-S., Jun E.-M., Lee B.W., Park S.M., Lee H.-J. (2025). Immune-Enhancing Effects of Two Potential Probiotic Strains *Latilactobacillus curvatus* WiKim0169 and *Pediococcus acidilactici* WiKim0170 in a Cyclophosphamide-Induced Immunosuppression Rat Model. Probiotics Antimicrob. Proteins.

[348] Fong F.L.Y., El-Nezami H., Mykkänen O., Kirjavainen P.V. (2022). The Effects of Single Strains and Mixtures of Probiotic Bacteria on Immune Profile in Liver, Spleen, and Peripheral Blood. Front. Nutr..

[349] Mousa W.K., Al Ali A. (2024). The Gut Microbiome Advances Precision Medicine and Diagnostics for Inflammatory Bowel Diseases. Int. J. Mol. Sci..

[350] Ashaolu T.J., Suttikhana I. (2026). Probiotic therapeutics: A critical review of mechanisms, clinical efficacy, and the frontier of precision microbiome modulation. Int. Immunopharmacol..

[351] Abraham L., Raise A., Beney L., Lapaquette P., Rieu A. (2025). Membrane vesicles produced by next-generation probiotics from the gut as innovative tools for human health. Gut Microbes.

[352] Jadhav N.K., Magdum A.B., Shinde K.V., Nimbalkar M.S. (2025). Next-Generation Probiotics: From Traditional Strains to Personalized Therapeutics. Mol. Nutr. Food Res..

[353] Wang X., Cheng Y., Huang J., Xu F., Jiang J., Nalinratana N., Jin L., Xue Y. (2026). Engineered probiotics for inflammatory bowel disease therapy: Mechanisms, delivery strategies, and precision medicine. Front. Microbiol..

[354] Balakrishna K., Naveena G., Kingston J.J. (2026). Postbiotics at the interface of microbial biotechnology and therapeutics: Industrial production, functional mechanisms, and clinical potentials. Arch. Microbiol..

[355] Zeng L., Qian Y., Cui X., Zhao J., Ning Z., Cha J., Wang K., Ge C., Jia J., Dou T. (2025). Immunomodulatory role of gut microbial metabolites: Mechanistic insights and therapeutic frontiers. Front. Microbiol..

[356] Rosas-Sánchez G.U., Muñoz-Carrillo J.L., Soria-Fregozo C. (2025). Transforming mental health: The future of personalized psychobiotics in anxiety and depression therapy. Front. Neurosci..

[357] Das A., Behera R.N., Kapoor A., Ambatipudi K. (2023). The Potential of Meta-Proteomics and Artificial Intelligence to Establish the Next Generation of Probiotics for Personalized Healthcare. J. Agric. Food Chem..

[358] D’amico F., Barone M., Tavella T., Rampelli S., Brigidi P., Turroni S. (2022). Host Microbiomes in Tumor Precision Medicine: How far are we?. Curr. Med. Chem..

[359] Ajibola F.O., Nwojiji E.C., Lawal M., Barakat S.T., Temitayo I.A., Ali V.E., Bakare-Abidola T. (2025). The role of the gut microbiome in immune modulation: Implications for autoimmune diseases and cancer therapy. World J. Biol. Pharm. Health Sci..

[360] Wei D., Sun Y., Han J., Liu J. (2025). Microbiota and cancer immunotherapy: Mechanisms, clinical implications, and precision therapeutics. Semin. Cancer Biol..

[361] Gupta M.K., Srivastava R. (2025). Gut Microbiome Interventions: From Dysbiosis to Next-Generation Probiotics (NGPs) for Disease Management. Probiotics Antimicrob. Proteins.

[362] Farizaldi R.I., Asmoro V.S. (2026). CRISPR-engineered probiotics for targeted production of inosine as an immunomodulatory metabolite to enhance cancer immunotherapy response: A review. World J. Adv. Res. Rev..

[363] Xue Y., Hu M., Cha S., Xue C., Dong N. (2025). Precision therapeutics for inflammatory bowel disease using engineered probiotics: Strategies and optimization. Acta Biomater..

[364] Patra D. (2024). Synthetic Biology-Enabled Engineering of Probiotics for Precision and Targeted Therapeutic Delivery Applications. Exon.

[365] Tegegne B.A., Abebaw D., Teffera Z.H., Fenta A., Belew H., Belayneh M., Jemal M., Getinet M., Baylie T., Tamene F.B. (2025). Microbial Therapeutics in Cancer: Translating Probiotics, Prebiotics, Synbiotics, and Postbiotics From Mechanistic Insights to Clinical Applications: A Topical Review. FASEB J..

[366] Quiroga-Centeno A.C., Atanasova K., Ebert M.P., Thomann A.K., Reindl W. (2025). Emerging microbiome-directed therapies in inflammatory bowel disease: Beyond diet modification and FMT. Semin. Immunopathol..

[367] Ma Y., Wang L., Hu H., Shieh A.R.-E., Li E., He D., He L., Liu Z., Paing T.M., Chen X. (2026). Composition and Function of Gut Microbiome: From Basic Omics to Precision Medicine. Genes.

[368] Duhan P., Gupta B., Ahmed M., Bansal P. (2025). Gut microbiome engineering with probiotics: Current trends and future directions. Discov. Appl. Sci..

[369] Mafe A.N., Sharifi-Rad J., Calina D., Ogunyemi A.J., Tubi A.O. (2025). Postbiotics in Functional Foods: Microbial Derivatives Shaping Health, Immunity and Next-Generation Nutrition. Food Front..

[370] Verma J., Paul D. (2026). Advancements in technology for developing recombinant live biotherapeutics. Prog. Mol. Biol. Transl. Sci..

[371] Johnstone J., Meade M., Lauzier F., Marshall J., Duan E., Dionne J., Arabi Y.M., Heels-Ansdell D., Thabane L., Lamarche D. (2021). Effect of Probiotics on Incident Ventilator-Associated Pneumonia in Critically Ill Patients. JAMA.

[372] Hempel S., Newberry S., Ruelaz A., Wang Z., Miles J.N.V., Suttorp M.J., Johnsen B., Shanman R., Slusser W., Fu N. (2011). Safety of probiotics used to reduce risk and prevent or treat disease. Evid. Rep. Technol. Assess..

[373] Kulkarni T., Majarikar S., Deshmukh M., Ananthan A., Balasubramanian H., Keil A., Patole S. (2022). Probiotic sepsis in preterm neonates—A systematic review. Eur. J. Pediatr..

[374] D’agostin M., Squillaci D., Lazzerini M., Barbi E., Wijers L., Da Lozzo P. (2021). Invasive Infections Associated with the Use of Probiotics in Children: A Systematic Review. Children.

[375] Muñoz P., Bouza E., Cuenca-Estrella M., Eiros J.M., Pérez M.J., Sánchez-Somolinos M., Rincón C., Hortal J., Peláez T. (2005). Saccharomyces cerevisiae Fungemia: An Emerging Infectious Disease. Clin. Infect. Dis..

[376] Manzoni P., Lista G., Gallo E., Marangione P., Priolo C., Fontana P., Guardione R., Farina D. (2011). Routine Lactobacillus rhamnosus GG administration in VLBW infants: A retrospective, 6-year cohort study. Early Hum. Dev..

[377] Thygesen J.B., Glerup H., Tarp B. (2012). *Saccharomyces boulardii* fungemia caused by treatment with a probioticum. BMJ Case Rep..

[378] Santino I., Alari A., Bono S., Teti E., Marangi M., Bernardini A., Magrini L., Di Somma S., Teggi A. (2014). *Saccharomyces Cerevisiae* Fungemia, a Possible Consequence of the Treatment of *Clostridium Difficile* Colitis with a Probioticum. Int. J. Immunopathol. Pharmacol..

